# Vaccine-boosted CAR T crosstalk with host immunity to reject tumors with antigen heterogeneity

**DOI:** 10.1016/j.cell.2023.06.002

**Published:** 2023-07-05

**Authors:** Leyuan Ma, Alexander Hostetler, Duncan M. Morgan, Laura Maiorino, Ina Sulkaj, Charles A. Whittaker, Alexandra Neeser, Ivan Susin Pires, Parisa Yousefpour, Justin Gregory, Kashif Qureshi, Jonathan Dye, Wuhbet Abraham, Heikyung Suh, Na Li, J. Christopher Love, Darrell J. Irvine

**Affiliations:** 1 David H. Koch Institute for Integrative Cancer Research, MIT, Cambridge, MA, 02139, United States; 2 Department of Materials Science and Engineering, MIT, Cambridge, MA, 02139, United States; 3 Department of Biological Engineering, MIT, Cambridge, MA, 02139, United States; 4 Department of Chemical Engineering, MIT, Cambridge, MA, USA; 5 Ragon Institute of Massachusetts General Hospital, Cambridge, MA, 02139, United States; 6 Broad Institute of MIT and Harvard, Cambridge, MA, 02142, United States; 7 Howard Hughes Medical Institute, Chevy Chase, MD, 20815, United States; 8 Department of Pathology and Laboratory Medicine, Perelman School of Medicine, University of Pennsylvania, Philadelphia, PA 19104, USA; 9 The Raymond G. Perelman Center for Cellular and Molecular Therapeutics, Children’s Hospital of Philadelphia, Philadelphia, PA 19104, USA; 10 Department of Bioengineering, School of Engineering and Applied Science, University of Pennsylvania, Philadelphia, PA 19104, USA; 11 Lead contact

## Abstract

Chimeric Antigen Receptor (CAR) T-cell therapy effectively treats human cancer, but loss of the antigen recognized by the CAR poses a major obstacle. We found that in vivo vaccine boosting of CAR T-cells triggers engagement of the endogenous immune system to circumvent antigen-negative tumor escape. Vaccine-boosted CAR-T promoted dendritic cell (DC) recruitment to tumors, increased tumor antigen uptake by DCs, and elicited priming of endogenous anti-tumor T-cells. This process was accompanied by shifts in CAR-T metabolism toward oxidative phosphorylation and was critically dependent on CAR T-derived IFN-γ. Antigen spreading induced by vaccine-boosted CAR T enabled a proportion of complete responses even when the initial tumor was 50% CAR-antigen-negative, and heterogenous tumor control was further enhanced by genetically amplifying CAR T IFN-γ expression. Thus, CAR T-cell-derived IFN-γ plays a critical role in promoting antigen spreading, and vaccine boosting provides a clinically-translatable strategy to drive such responses against solid tumors.

## In-brief

Vaccine boosting modifies CAR T cell metabolism and promotes crosstalk between CAR T cells and endogenous immunity to elicit and sustain antigen spreading, thereby effectively treating tumors with antigen heterogeneity.

## Graphical Abstract

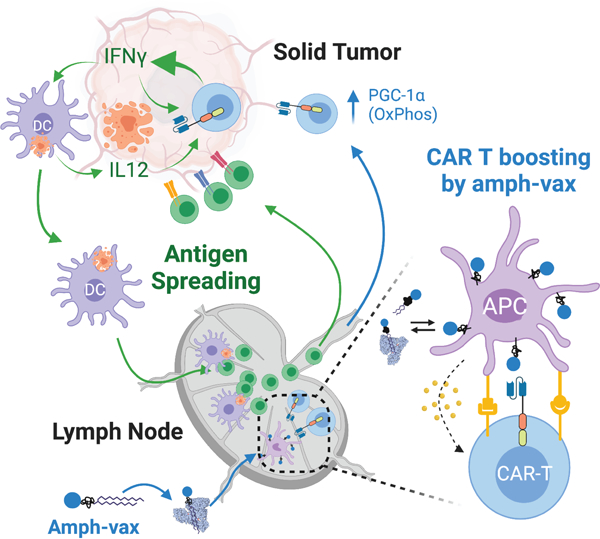

## INTRODUCTION

Adoptive cell therapy (ACT) using chimeric antigen receptor (CAR) T-cells has revolutionized the treatment of relapsed/refractory CD19^+^ B-cell acute lymphoblastic leukemia and lymphomas^1–5^. In the setting of solid tumors, CAR T therapy has been less successful so far, though progress is being made to address issues such as limited tumor infiltration, poor CAR T functionality and persistence^1,6–8^. However, two key challenges in the treatment of tumors with CAR T-cells are pre-existing antigenic heterogeneity, where not all tumor cells express the antigen targeted by the CAR, and antigen loss occurs during treatment. For example, a recent first-in-human clinical trial assessing CAR T-cells targeting mutant EGFRvIII in glioblastoma resulted in the emergence of EGFRvIII^null^ tumors^9^. Even in leukemia patients initially responding to CD19 CAR T therapy, loss or downregulation of the CD19 antigen has been frequently observed and often results in disease relapse^10^. An additional mechanism of antigen loss is via inflammation-induced dedifferentiation in melanomas^11^. These observations highlight the need for novel approaches to address antigen-loss-mediated tumor escape.

Antigen spreading (AS) is the induction and amplification of immune responses to secondary antigens distinct from the original therapeutic target^12^. In the setting of adoptive cell therapy, strategies to target one surface-expressed antigen using CAR T-cells while inducing endogenous T-cell responses against additional tumor antigens would be an attractive approach to overcome tumor heterogeneity and antigen loss-mediated escape. Accumulating evidence suggests that AS can be elicited and may contribute to the overall therapeutic outcome during cancer immunotherapy. For example, recruitment and expansion of tumor-specific T-cells that were undetectable prior to therapy was found in patients receiving Ipilimumab^13^. Some cancer patients treated with neoantigen vaccines also exhibited AS towards shared neoantigens or cancer testis antigens^14,15^. In addition, increased anti-tumor antibody responses or weak T-cell responses were documented in a few cases of pre-clinical and clinical CAR T-cell therapy^16–18^. Nonetheless, to date there is limited evidence of CAR T-cell therapy itself inducing therapeutically meaningful AS. Preclinically, a majority of CAR-T studies employ immunodeficient mice that by definition exclude endogenous T-cell responses. In immunocompetent mouse models, CAR T therapy itself seems to have limited ability to trigger AS especially in solid tumors^21^. By contrast, CAR T-cells engineered with additional immune response-provoking molecules, including FLT3L^22^, CD40L^23^, IL-12^24,25^, IL-18^26^, IL-7/CCL19^27^, or when used in combination with oncolytic viruses^28,29^, have been reported to exhibit increased anti-tumor activity as well as evidence for AS. However, introduction of such additional effector functions to CAR T-cells with uniform activity across patients can be challenging and lead to new safety risks^30,31^. More importantly, irrespective of the CAR T-cell modality, mechanisms by which AS is promoted during adoptive cell therapy remain poorly understood.

We recently described an approach to amplify CAR-T activity in solid tumors by vaccine-like boosting of CAR T-cells via their chimeric antigen receptor in lymph nodes^32^. This was accomplished by the synthesis of CAR ligands conjugated to an amphiphilic polymer-lipid tail, which following parenteral injection, efficiently traffic to draining lymph nodes and decorate the surfaces of macrophages and dendritic cells (DCs) with CAR-T ligands. CAR T-cells encountering ligand-decorated DCs in the lymph node receive stimulation through the CAR in tandem with native costimulatory receptor signals and cytokine stimulation from the ligand-presenting cell, leading to CAR T-cell expansion and enhanced functionality. Vaccine boosting of CAR T-cells via administration of these “amph-ligands” together with vaccine adjuvants substantially enhanced tumor rejection by CAR T-cell therapy, and unexpectedly, was accompanied by the development of endogenous anti-tumor T-cell responses^32^.

Here we used this approach of CAR-T therapy in tandem with vaccine boosting as a model setting to understand the role of antigen spreading in the clearance of antigenically heterogenous solid tumors, and to define mechanisms underlying AS. In multiple murine syngeneic tumor models, we found that AS elicited by CAR T-cell therapy using second-generation CARs was negligible. However, endogenous T-cell priming could be markedly induced by vaccine boosting of CAR T-cells, even in the context of lymphodepletion preconditioning. This process was critically dependent on IFN-γ, and enhanced IFN-γ expression induced either by vaccine boosting or genetic engineering enabled CAR T-cells to control solid tumors with preexisting antigen heterogeneity.

## RESULTS

### Vaccine boosting enables CAR T-cells to elicit endogenous CD4^+^ and CD8^+^ T-cell responses in multiple tumor models

**Figure 1.**
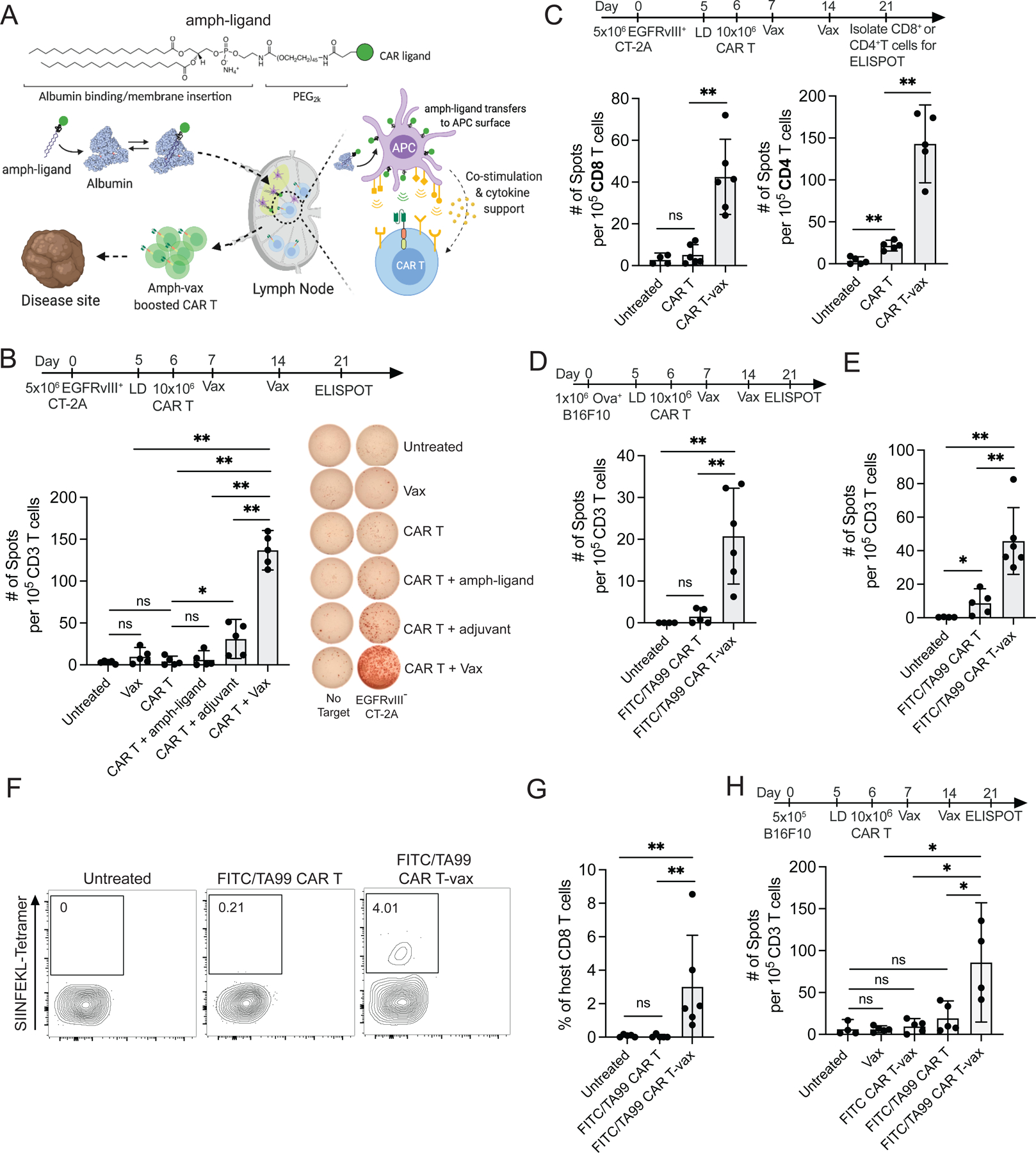
Vaccine boosting enables CAR T-cells to elicit endogenous T-cell responses in multiple tumor models. (A) Schematic of CAR T-vax therapy. Created with BioRender.com. (B) IFN-γ ELISPOT. Mice bearing EGFRvIII^+^CT-2A tumors (n=5) treated with or without CAR T + various combinations of vaccine components. (C) Priming of endogenous CD8^+^ and CD4^+^ T-cells in EGFRvIII^+^CT-2A tumor-bearing mice (n=5–6) following CAR-T ± vax as measured by IFN-γ ELISPOT. (D-G) Mice (n=5–6) bearing OVA^+^ B16F10 tumors received FITC/TA99 CAR T-vax. (D) IFN-γ ELISPOT measuring OVA-specific endogenous T-cell responses. (F) IFN-γ ELISPOT measuring endogenous T-cell responses against Trp1^−/−^ B16F10 cells. (F-G)Tetramer-staining showing representative flow cytometry staining (F) and mean percentages of SIINFEKL tetramer^+^ endogenous T cells (G). (H) IFN-γ ELISPOT. Mice (n=4–5) bearing B16F10 tumors were treated with vax only, FITC-CAR T, or FITC/TA99 CAR T ± vax. Error bars show mean ± 95% CI. ***, p<0.0001; **, p<0.01; *, p<0.05; n.s., not significant by one-way ANOVA with Tukey’s post-test.

The amph-ligand-based vaccine boosting approach is illustrated schematically in Figure 1A: Amph-ligands are comprised of a ligand for a selected CAR linked to a hydrophobic phospholipid tail via a poly(ethylene glycol) (PEG) spacer. Upon co-injection with a suitable vaccine adjuvant at a site distal from the tumor, amph-ligands bind to albumin present in the interstitial fluid and are efficiently transported to the downstream draining lymph nodes (dLNs)^33^. Within the densely packed LN parenchyma, the amph-ligand transfers into cell membranes, decorating primarily the surface of macrophages and dendritic cells that line the subcapsular sinus and collagen conduits carrying lymph into the T-cell paracortex^32^. The co-administered adjuvant simultaneously activates DCs in the dLN to upregulate expression of costimulatory receptors and produce cytokines. CAR T-cells encountering ligand-decorated, activated DCs are stimulated in a manner mimicking natural T-cell priming, leading to CAR T-cell expansion and enhanced effector functions. Unexpectedly, we found that vaccine-boosted CAR T-cells also induce the expansion of endogenous anti-tumor T-cell responses^32^.

We first assessed how the composition of the boosting vaccine impacts this antigen spreading response in a syngeneic murine EGFRvIII^+^CT-2A glioblastoma model. In this model, CAR T-cells targeting mutant EGFR (mEGFRvIII) are vaccine boosted using an amph-ligand comprised of an mEGFRvIII-derived peptide epitope recognized by the CAR T-cells^32^ (Figure S1A) combined with the potent STING agonist vaccine adjuvant cyclic di-GMP. Animals received lymphodepletion, followed one day later by s.c. injection of amph-ligand alone, adjuvant alone, or the full vaccine (amph-ligand + adjuvant). Amph-ligand/adjuvant was administered again 7 days later as a second boost, and then splenocytes were isolated at day 21 and co-cultured with irradiated EGFRvIII^−^ CT-2A cells in an IFN-γ ELISPOT assay to detect endogenous T-cell responses against non-CAR T-targeted antigens (Figure 1B). Endogenous lymphocyte and dendritic cell numbers were still recovering across the time course of these experiments following lymphodepletion (Figure S1B), However, their recovery was sufficiently rapid to permit robust *de novo* endogenous T-cell priming, consistent with prior preclinical studies reporting antigen spreading following lymphodepleting therapy^22^. Injection of the amph-ligand alone without adjuvant failed to initiate endogenous T-cell priming, while CAR T treatment in tandem with vaccine adjuvant alone elicited low but detectable endogenous T-cell responses (Figure 1B). However, the full vaccine (amph-ligand + adjuvant) led to 6-fold greater endogenous T-cell priming. This antigen spreading response did not reflect a direct effect of the vaccine on tumors, as inoculating tumors distal from the vaccine injection site did not change the antigen spreading response (Figure S1C). Similar magnitudes of endogenous T-cell priming were also observed with alternative adjuvants (TLR7/8 agonist Resiquimod, or the TLR9 agonist CpG, Figure S1D). Further analysis revealed that while CAR T therapy elicited no statistically significant endogenous anti-tumor CD8^+^ T-cell response and only a weak (but detectable) CD4^+^ T-cell response compared to untreated tumors, CAR-T combined with amph-ligand vaccination (hereafter, CAR T-vax) primed robust responses from both the CD4^+^ and CD8^+^ T-cell compartments (Figure 1C).

To evaluate AS in a tumor model carrying a defined T-cell antigen, we assessed vaccine-boosted CAR-T treatment in a second model of B16F10 murine melanoma expressing the surrogate antigen ovalbumin (OVA), treated with bispecific FITC/TA99 CAR T-cells recognizing FITC and the melanoma-associated antigen Trp1 (Figure S1E). In this model, CAR T-cells are boosted by vaccination with amph-FITC and attack the tumor through Trp1 recognition. By ELISPOT, we observed host T-cell responses to both the model antigen OVA (Figure 1D) and B16F10 neoantigens (Figure 1E), but only when mice received both CAR T-cells and vaccine boosting. As shown in Figure 1F–G, quantifying CD8^+^ T-cells targeting the immunodominant OVA epitope SIINFEKL by peptide-MHC tetramer staining, no OVA-specific T-cells were detected in mice receiving CAR T-cells alone, but CAR T-vax therapy elicited a readily detectable SIINFEKL-specific T-cell response. Finally, to evaluate whether vaccine boosting could promote antigen spreading in a setting of a CAR T-cell targeting an endogenous tumor-associated antigen without the presence of an overexpressed neoantigen, we treated parental B16F10 tumors with FITC/TA99 CAR T-cells (Figure 1H). FITC vaccine alone or FITC/TA99 CAR T-cells alone elicited no AS above baseline. Vaccine boosting of a CAR that cannot recognize the tumor (FITC CAR T-vax) also failed to elicit antigen spreading, but vaccine boosting of FITC/TA99 CAR T-cells led to readily detectable host T-cell responses directed against non-Trp1 tumor antigens (Figure 1H). Thus, in three different tumor models using two different CARs, CAR T-cell treatment combined with amph-vax boosting promoted antigen spreading.

### Vaccine-boosted CAR T-cells drive functional and phenotypic changes in endogenous T-cells

**Figure 2.**
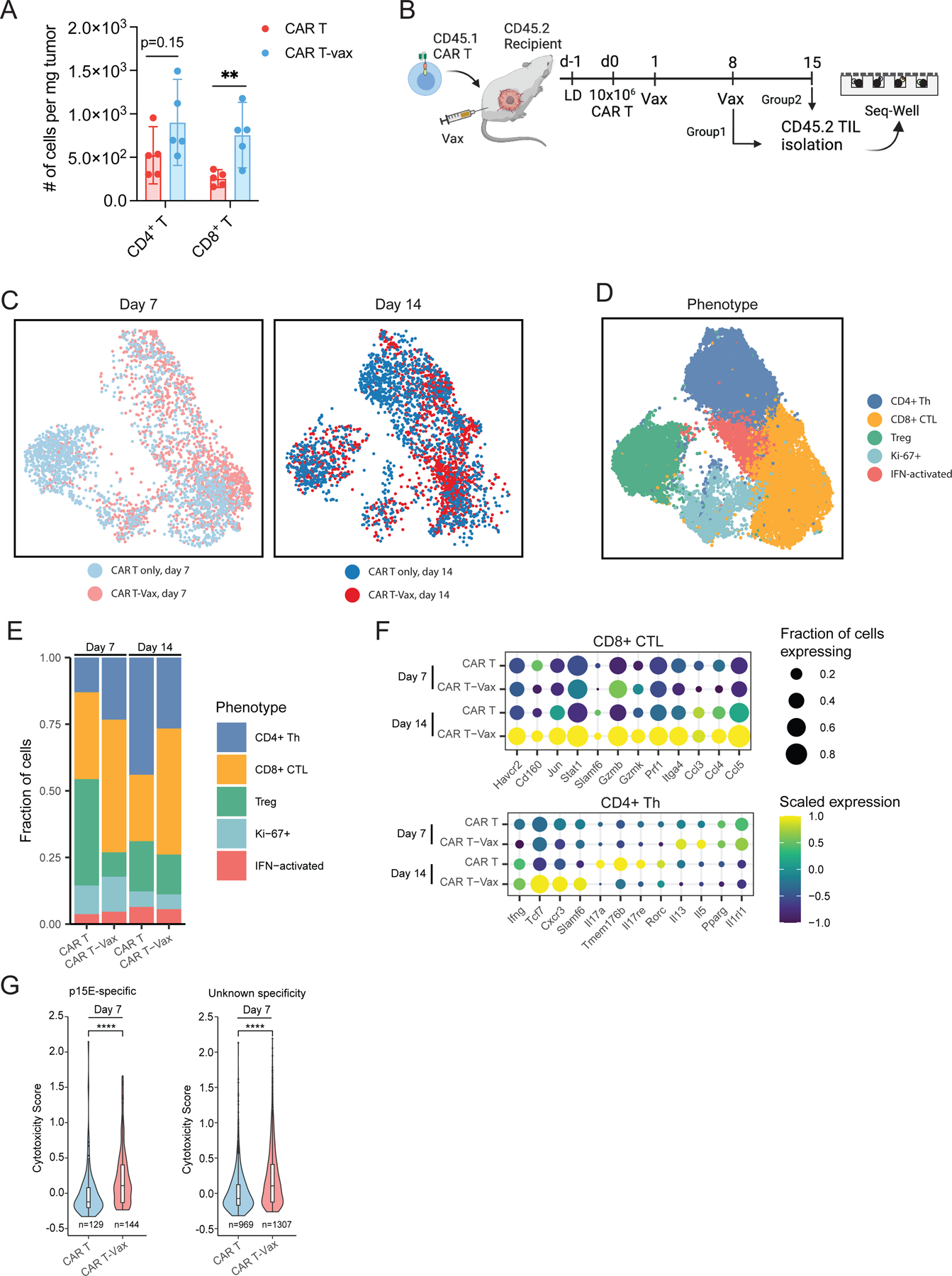
Endogenous tumor-infiltrating T cells show transcriptional changes associated with enhanced anti-tumor activity in response to CAR T-vax therapy. (A) Enumeration of intratumoral host T-cells in tumor-bearing mice (n=5) post CAR T ± vax treatment. (B-G) Tumor-bearing mice were treated with CAR T ± vax, TILs were isolated for scRNA-seq. (B) Experimental setup/timeline. Created with BioRender.com. (C) UMAP of endogenous T-cells obtained from tumors. (D) Curated clusters based on signature gene expression. (E) Stacked charts showing proportions of each T-cell cluster. (F) Dot plots showing differential expression of signature genes in endogenous CD8^+^ CTLs or CD4^+^ Th cells. (G) Cytotoxicity score of endogenous p15E-specific TILs and TILs of unknown specificity. All mice bear EGFRvIII^+^CT-2A tumors. Error bars are mean ± 95% CI, ****p<0.0001; **, p<0.01; n.s., not significant by Student’s t-test for A, by two-sided Wilcoxon rank-sum test for G.

We analyzed tumor-infiltrating lymphocytes (TILs) by flow cytometry on day 7 post CAR T-vax treatment and observed substantially increased endogenous CD8^+^ TILs and a trend toward increased CD4^+^ cells (Figure 2A). A similar increase of host T-cell infiltration was found by adding vaccine boosting to CAR-T therapy treatment of OVA-expressing CT-2A tumors, including a 3-fold increase in *bona fide* tumor-antigen (OVA)-specific TILs (Figure S1F). We isolated host CD4^+^ and CD8^+^ TILs 7 or 14 days after treatment and carried out single-cell RNA-seq and paired α/β TCR sequencing on the recovered host lymphocytes (Figure 2B). Quality single-cell transcriptomes were obtained for 21,835 T-cells (Figure 2C–D). Unsupervised clustering of the transcriptome data revealed five major endogenous T-cell subsets: CD8^+^ cytotoxic T lymphocytes (CTLs, expressing *Cd8a*, *Ccl5*, *Pdcd1*), CD4^+^ T helper cells (*Cd4*, *Cd40lg*), Tregs (*Foxp3, Il2ra, Ikzf2*), a proliferating Ki-67^+^ population that included both CD4^+^ and CD8^+^ cells (*Mki67, Top2a*), and a small population of IFN-stimulated T-cells (characterized by expression of *Ifit1, Ifit3, Isg15*) (Figure 2D–E, S2A, Supplemental Table 1), as has been described previously^34,35^. We observed an increase in the frequency of the CD8^+^ CTL population in mice treated with CAR T-vax at both day 7 and day 14. Interestingly, we also observed a transient decrease in the frequency of Tregs at day 7 in mice treated with CAR T-vax compared to those treated with CAR-T alone (Figure 2E).

We computed differentially expressed genes between CD8^+^ CTLs recovered from mice treated with CAR T-vax vs. CAR T alone. At day 14, CD8^+^ T-cells from CAR T-vax-treated mice upregulated transcripts associated with both cytotoxicity (*Gzmb, Gzmk*) and T-cell activation (*Havcr2*) relative to the CΑR-T alone group (Figure 2F, S2B–C, Supplemental Table 2). We validated these findings at the protein level by carrying out flow cytometry analysis of endogenous TILs. Compared to CAR T only therapy, vaccine boosting did not change the proportion of PD-1^+^TIM-3^+^ or PD-1^+^TIM-3^−^ endogenous TILs (Figure S3A), but did enhance IFN-γ, TNF-α, and granzyme B expression in both populations (Figure S3B–C). Among CD4^+^ cells, we found an elevation of transcripts associated with Th17 function (*Rorc, Il17a, Il17re*) among mice treated with CAR-T alone at day 14 compared to day 7 (Figure 2F, Supplemental Table 2). By contrast, CD4^+^ Th cells from CAR T-vax-treated mice upregulated genes associated with Th1 function (*Ifng*, *Cxcr3*) and self-renewal (*Slamf6*, *Tcf7*) (Figure 2F, S2D–E), suggesting that the vaccine may also promote anti-tumor phenotypes among CD4^+^ TILs. Next, we sought to assess how CAR T-vax affects TILs according to their antigen specificities. Using data generated in a recent study defining TCR sequences specific for a common murine endogenous retroviral antigen p15E (Grace et al., 2022) that is also expressed by CT-2A cells (Figure S3D–E), we assessed the transcriptional state of tumor-specific endogenous TILs. At day 7, both p15E-specific T-cells and TILs of unknown specificity from CAR T-vax-treated mice exhibited significantly higher cytotoxicity than TILs from animals treated with CAR-T alone (Figure 2G, S2F–G, S3F, Supplemental Table 3). Overall, this analysis suggests that the addition of the vaccine to CAR-T therapy increases the anti-tumor potential of tumor-infiltrating CD8^+^ T-cells and skews the differentiation of tumor-infiltrating CD4^+^ T-cells to a Th1 phenotype.

### Vaccine-driven antigen spreading prevents relapse of antigen-loss variants and enables control of antigenically heterogenous tumors

**Figure 3.**
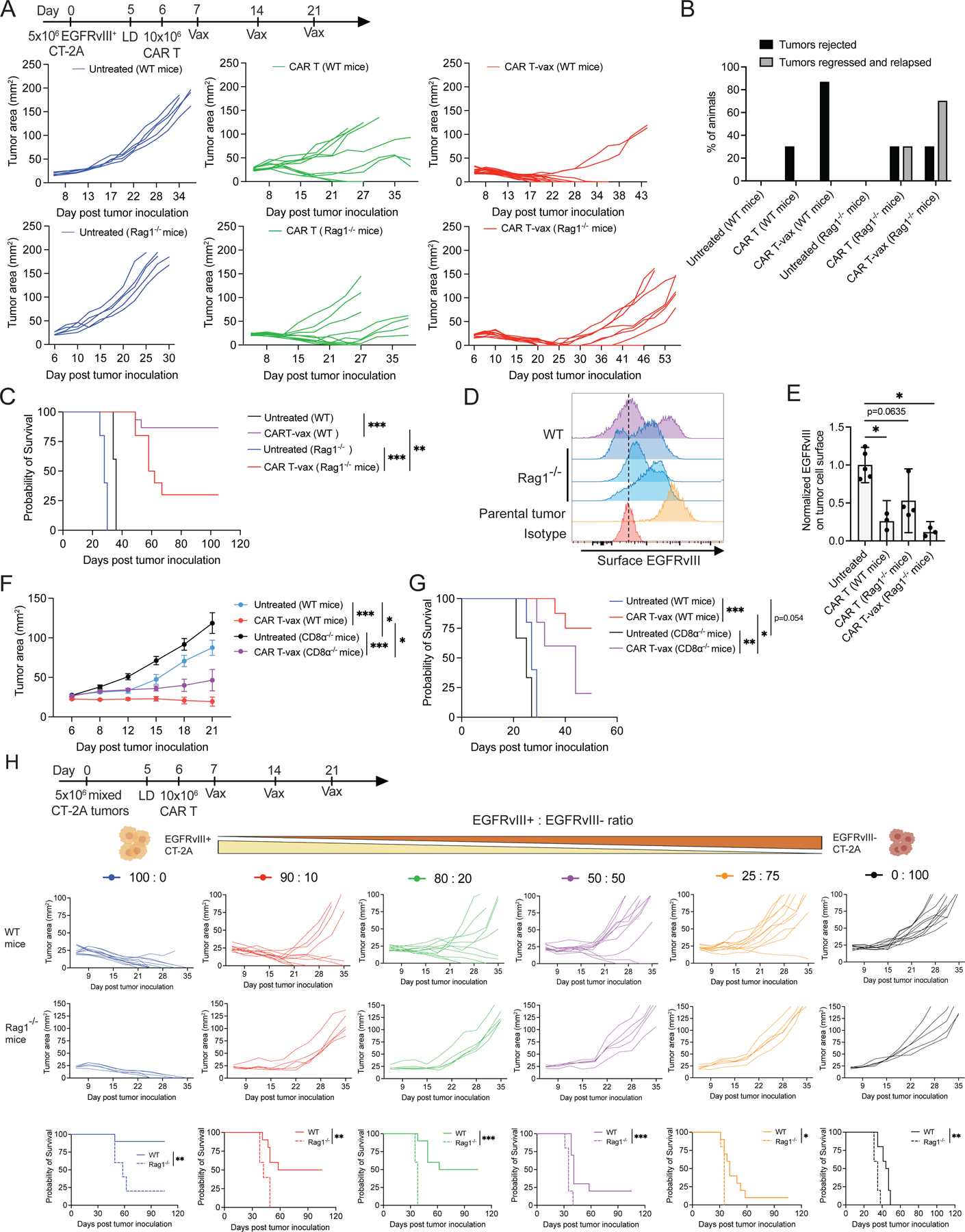
Vaccine-driven antigen spreading is required for long-tumor tumor control in immunocompetent mice. (A-E) Treatment of tumor-bearing WT or Rag1^−/−^ mice with WT CAR-T ± vax. (A) Tumor growth in individual mice. Untreated, *n* = 5; CAR-T in WT mice, *n* = 10; CAR T-vax, *n* = 15 and 10 in WT and Rag1^−/−^ mice, respectively. (B) Percentage of mice that completely rejected tumors or experienced tumor relapse. (C) Overall survival. (D-E) Surface EGFRvIII expression (D) and mean expression normalized to untreated tumors (E) on parental or representative relapsed tumors from WT and Rag1^−/−^ mice following CAR T-vax treatment. (F-G) Tumor-bearing WT or CD8α^−/−^ mice (n=5–8) ± CAR T-vax treatment. (F) Tumor growth. (G) Overall survival. (H) Individual tumor growth and overall survival of WT (*n*=10) or Rag1^−/−^ mice (*n*=5) bearing heterogeneous CT-2A tumors upon CAR T-vax treatment. EGFRvIII^+^:EGFRvIII^−^ cells were pre-mixed at the indicated ratios. All mice in A-G bear EGFRvIII^+^CT-2A tumors. Error bars are mean ± 95% CI, ***, p<0.0001; **, p<0.01; *, p<0.05 by Student’s t-test for E, by Log-rank (Mantel-Cox) test for C,G-H, by two-way ANOVA with Tukey’s post-test for F.

To determine if endogenous T-cells impact the outcome of CAR T-vax treatment, we treated wildtype (WT) or RAG1^−/−^ mice bearing mEGFRvIII^+^CT-2A tumors with CAR T-cells ± vaccine boosting. CAR T-vax therapy in WT mice led to much greater tumor control compared to CAR T-cells alone (Fig. 3A–C). In RAG^−/−^ animals, CAR T-vax treatment also elicited a high frequency of initial tumor regressions, but a majority of tumors relapsed 20–50 days post treatment (Figure 3A–C). Analysis of relapsed tumors revealed that loss or down-regulation of EGFRvIII on tumor cells was a major escape mechanism in the RAG^−/−^ animals (Figure 3D–E). These data suggested that endogenous lymphocytes are critical for the high frequency of complete responses observed in WT animals. Given the substantial effect of CAR T-vax treatment on cytotoxic effector gene expression in endogenous CD8^+^ T cells (Figure 2F), we evaluated the importance of endogenous CD8^+^ T cells in tumor control, by comparing CAR T-vax treatment in WT versus CD8α^−/−^ tumor-bearing mice. Early tumor growth control was only modestly affected in the absence of endogenous CD8 T cells (Figure 3F), but long-term survival was almost completely abolished (Figure 3G).

Encouraged by these findings, we tested whether endogenous T-cell priming could enable CAR T-cells to eliminate tumors with pre-existing antigenic heterogeneity. To this end, we inoculated a mixture of EGFRvIII^+^ CT-2A cells and parental EGFRvIII^−^ CT-2A cells at defined ratios into both WT and RAG1^−/−^ mice (Figure 3H). We previously showed that these two CT-2A variants have similar growth rates in WT mice^32^. When 100% of the tumor cells express EGFRvIII, CAR T-vax therapy elicited comparable initial tumor regressions in both WT and RAG1^−/−^ mice, but long-term remission was only achieved in WT animals (Figure S3G). More strikingly, in heterogeneous tumors comprised of as little as 10% EGFRvIII^−^ cells, CAR T-vax therapy delayed tumor progression but induced no actual regressions in RAG1^−/−^ mice. By contrast, CAR T-vax treatment cured ~50% animals bearing tumors with up to 20% EGFRvIII^−^ cells and could still achieve complete responses in a small proportion of animals when the EGFRvIII^−^ population was 50% of the tumor mass at time zero. To confirm that vaccine boosting of CAR T-cell therapy could augment heterogeneous tumor control in the setting of a non-overexpressed tumor antigen, we also treated melanoma tumors comprised of a mixture of 80% parental and 20% Trp1^−/−^ B16F10 tumor cells with bivalent FITC/TA99 CAR T-cells and amph-FITC vaccine. Treatment of this mixed tumor elicited readily detectable antigen spreading to non-Trp1 antigens (Figure S3H) and controlled tumor growth (Figure S3I). The drastic difference of therapeutic outcome in WT vs RAG1^−/−^ mice demonstrates the pivotal role endogenous T-cells and AS can play in controlling tumors with pre-existing antigenic heterogeneity.

### Vaccine boosting induces cell-intrinsic enhancements in CAR T-cell function

**Figure. 4.**
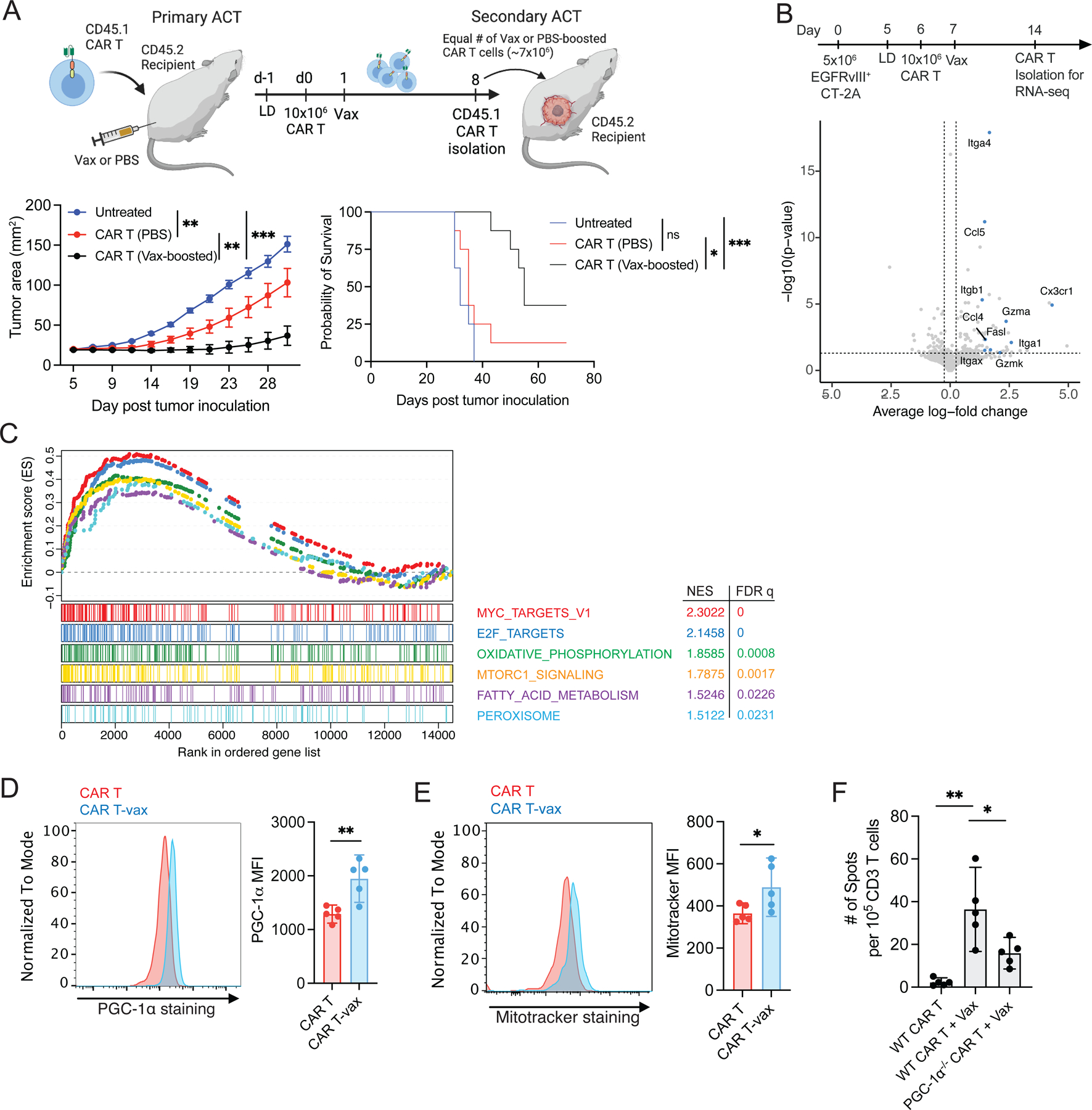
Vaccine boosting induces cell-intrinsic enhancements in CAR T-cell function that include metabolic reprogramming. (A) Tumor growth (left) and overall survival (right) of tumor-bearing mice (*n*=8) after receiving vaccine-boosted or non-boosted CAR T cells. Created with BioRender.com. (B-C) Tumor-bearing mice received WT CAR T ± vax treatment, and CAR T-cells were isolated from spleens and tumors for RNA-seq. (B) Volcano plot showing differential gene expression in splenic CAR T-cells. (C) GSEA showing enriched pathways in intratumoral CAR T-cells. (D-E) Intracellular PGC-1α expression (D) and mitochondrial mass (E) in intratumoral CAR T cells from mice (n=5) 7 days post treatment with WT CAR T ± vax. (F) IFN-γ ELISPOT. Tumor-bearing mice (n=5) treated with WT CAR T ± vax or PGC-1α^−/−^ CAR T-vax. All mice bear EGFRvIII^+^CT-2A tumors. Error bars are mean ± 95% CI, **, p<0.01; *, p<0.05 by Student’s *t*-test for D-E, and one-way ANOVA with Tukey’s post-test for F.

We next sought to understand how amph-vax boosting promotes endogenous T-cell priming. We first tested whether the anti-tumor efficacy of vaccine boosting was simply driven by increased numbers of CAR T-cells, vs. a change in CAR T function. CAR T-cells were transferred into non-tumor bearing mice, vaccine boosted (or not as controls), and then isolated 7 days later from the two groups and transferred at equal numbers into new tumor-bearing recipient mice (Figure 4A). This approach revealed that even when the same number of CAR T-cells were present, vaccine-boosted CAR T still exhibited enhanced tumor control and long-term animal survival, suggesting that vaccine boosting enhances the intrinsic per-cell functionality of CAR T-cells (Figure 4A).

To gain an unbiased view of changes in CAR-T function, we carried out bulk RNA-seq on CAR T-cells 8 days after adoptive transfer, with or without vaccine boosting. Vaccination increased the expression of genes associated with effector function and cell trafficking (e.g, *FasL*, *Gzma*, *Gzmk, Ccl5, Itgb1*) in CAR T-cells recovered from the spleen (Figure 4B, Supplemental Table 4); we confirmed the expression of several of these genes by quantitative PCR (Figure S4A). Gene set enrichment analysis (GSEA) of tumor-infiltrating cells further revealed that vaccine-boosted CAR T-cells maintained a high proliferative potential, as evidenced by elevated Myc and E2F target genes (Figure 4C). Vaccine-boosted cells also showed a significant upregulation of metabolic pathways, including oxidative phosphorylation (OXPHOS), MTORC1 signaling, fatty acid metabolism, and peroxisome signaling (Figure 4C). Prompted by these transcriptional signatures, we analyzed the intracellular expression of PGC-1α, a master transcription factor controlling many genes and pathways involved in OXPHOS^36^, and found that vaccine boosting increased PGC-1α levels in CAR T-cells (Figure 4D). PGC-1α is involved in mitochondria generation and maintenance^37^, and we noted increased mitochondria levels in vaccine-boosted CAR T-cells (Figure 4E). Notably, endogenous T-cell priming was significantly reduced ~50% following CAR T-vax treatment with PGC-1α^−/−^ CAR T-cells compared to WT CAR T (Figure 4F). Hence, metabolic reprogramming in vaccine-boosted CAR T-cells is one factor promoting antigen spreading.

### Enhanced IFN-γ production by vaccine-boosted CAR T-cells is critical for induction of antigen spreading

**Figure. 5.**
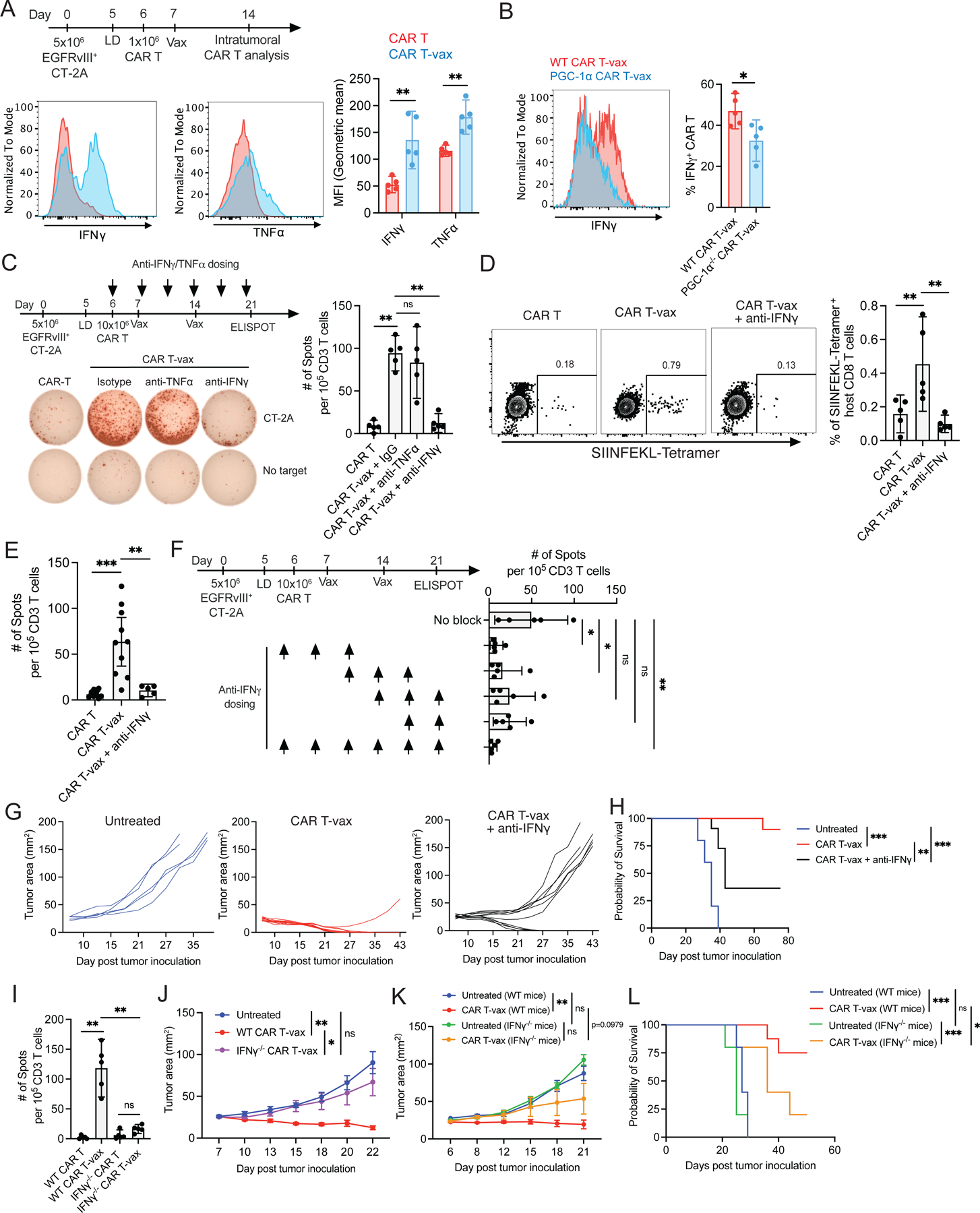
Enhanced IFN-γ production by vaccine-boosted CAR T-cells is critical for antigen spreading. (A) IFN-γ and TNF-α expression in intratumoral CAR T-cells from mice (n=5) treated with WT CAR T ± vax. (B) IFN-γ expression in intratumoral CAR T-cells from mice (*n*=5) 7 days post treatment with WT or PGC-1α^−/−^ CAR T-vax. (C) IFN-γ ELISPOT. Tumor-bearing mice (*n*=5) treated with WT CAR T or WT CAR T-vax + isotype control antibody (IgG), anti-TNF-α or anti-IFN-γ. (D-E) OVA^+^EGFRvIII^+^CT-2A tumor-bearing mice(*n*=5–10) treated by WT CAR T or WT CAR T-vax ± anti-IFN-γ. Endogenous OVA-specific T-cell responses detected by SIINFEKL-tetramer staining (D) and IFN-γ ELISPOT (E). (F) IFN-γ ELISPOT. Tumor-bearing mice (*n*=5) treated with WT CAR T-vax ± anti-IFN-γ at indicated time points. (G-H) Tumor growth (G) and overall survival (H) of mice left untreated (n=5) or treated (n=10) with WT CAR T-vax ± anti-IFN-γ. (I) IFN-γ ELISPOT. Tumor-bearing mice (n=5) treated with WT or IFN-γ^−/−^ CAR T ± vax. (J) Tumor growth in mice (*n*=5) left untreated or treated with WT or IFN-γ^−/−^ CAR T-vax. (K-L) Tumor growth (K) and overall survival (L) of WT or IFN-γ^−/−^ mice (n=5–8) treated with or without WT CAR T-vax therapy. All mice bear EGFRvIII^+^CT-2A tumors. Error bars are mean ± 95% CI, ***, p<0.0001; **p<0.01; *, p<0.05, ns, not significant by Student’s *t*-test for A-B, by one-way ANOVA with Tukey’s post-test for C-F, I, by two-way ANOVA with Tukey’s post-test for J-K, and by Log-rank (Mantel-Cox) test for H and L.

OXPHOS has been shown to be critical for maintaining the polyfunctionality of T-cells within the TME^38^, and we previously observed that vaccine-boosted CAR T-cells recovered from the peripheral blood showed increased cytokine production^32^. To determine if this enhanced effector function was maintained in tumors and impacted antigen spreading, we analyzed IFN-γ and TNF-α expression in TILs and found that both cytokines were markedly upregulated in vaccine-boosted CAR T-cells (Figure 5A). This enhanced cytokine production is partially linked to vaccine-induced metabolic changes, because IFN-γ expression was reduced in PGC-1α-deficient CAR T-cells (Figure 5B). Interestingly, although CAR T-cells ± vaccine exhibited comparable levels of PD-1 and Tim-3 expression, high-level cytokine production was maintained in both PD-1^+^Tim-3^−^ and PD-1^+^Tim-3^+^ CAR T-cells that received vaccine boosting (Figure S4B–D). To assess the role of these cytokines in AS, we treated tumor-bearing mice with CAR T-vax therapy in the presence of neutralizing antibodies against IFN-γ or TNF-α. Therapy in the presence of isotype control or TNF-α-blocking antibodies had no impact on endogenous T-cell priming, but IFN-γ blockade completely abrogated AS, including both CD4^+^ and CD8^+^ T cell responses (Figure 5C, Figure S4E–F). To confirm this result, we repeated IFN-γ blockade experiments in a second model of OVA^+^EGFRvIII^+^CT-2A cells. CAR T-vax treatment expanded OVA-specific T-cells and induced IFN-γ-producing T-cells recognizing SIINFEKL, as determined by peptide-MHC tetramer staining and ELISPOT, respectively (Figure 5D–E). However, IFN-γ neutralization during treatment eliminated the OVA-specific T-cell response (Figure 5D–E). Further, endogenous T-cell infiltration and functional enhancement were also repressed by IFN-γ blockade (Figure S5, Supplemental Table 5). Administration of blocking antibodies at different time points during therapy revealed that IFN-γ was most critical for promoting AS during the first week of treatment (Figure 5F). Blockade of IFN-γ using neutralizing antibodies also greatly reduced the efficacy of the treatment (Figure 5G–H).

To determine what cells were the key producers of IFN-γ, we tested CAR T-vax therapy employing IFN-γ-deficient CAR T-cells; this treatment elicited no antigen spreading (Figure 5I) and tumor control was lost, demonstrating an important role for CAR T-derived cytokine (Figure 5J). Early tumor control trended toward lower efficacy when CAR T-vax therapy was applied to tumor-bearing IFN-γ-deficient hosts, but this did not reach statistical significance (Figure 5K). However, long-term tumor control and overall survival was strongly reduced in IFN-γ^−/−^ mice (Figure 5L). Thus, CAR T-vax therapy amplifies CAR T-cell-derived IFN-γ that is critical for initial tumor control and antigen spreading, but also requires host-derived IFN-γ at later time points in the treatment, consistent with the important role for endogenous T cells in preventing tumor relapse.

### IFN-γ sustains vaccine-boosted CAR T effector functions, promotes DC recruitment and antigen uptake, and triggers IL-12-mediated CAR T-DC crosstalk

**Figure. 6.**
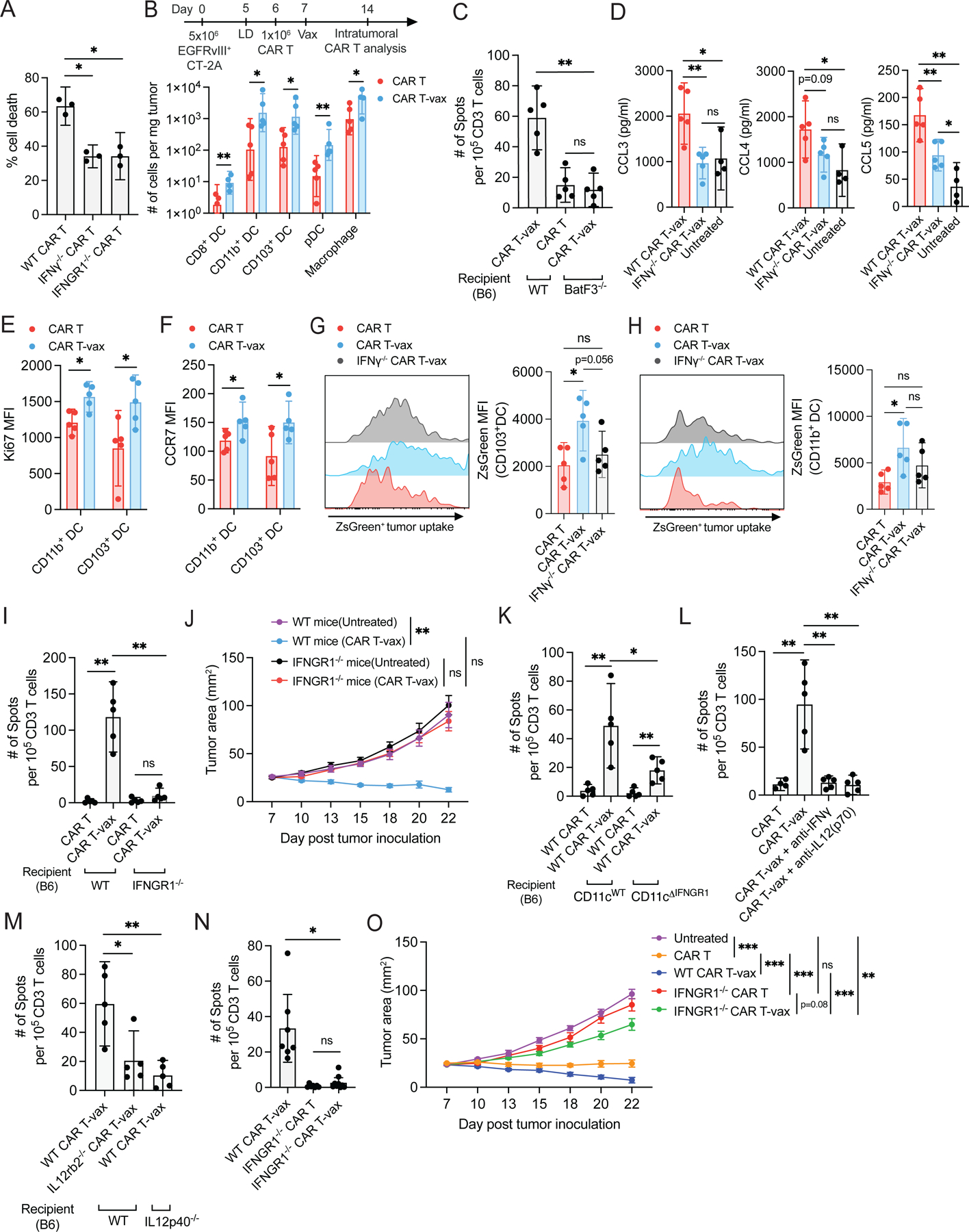
DCs regulate CAR T-cell-induced antigen spreading through enhanced tumor antigen acquisition and IFN-γ-IL-12 crosstalk. (A) EGFRvIII^+^ CT-2A cell killing by WT, IFN-γ^−/−^, or IFNGR1^−/−^ CAR T-cells *in vitro* (*n*=3). (B) Enumeration of tumor-infiltrating immune cells in mice (*n* = 4–5) receiving WT CAR T ± vax. See supplemental methods for phenotyping details. (C) IFN-γ ELISPOT. Tumor-bearing WT or Batf3^−/−^ mice (*n*=5) treated with WT CAR T ± vax. (D) Tumor-bearing mice were left untreated (n=4) or treated with WT or IFN-γ^−/−^ CAR T-vax (*n*=5). Shown are chemokine expression in tumors 7 days post treatment. (E-F) Ki67 (E) and CCR7(F) expression in intratumoral CD103^+^ DCs and CD11b^+^ DCs from mice (*n*=5) treated with WT CAR T ± vax. (G-H) Mice bearing ZsGreen^+^EGFRvIII^+^CT-2A tumors were treated with WT CAR T, WT CAR T-vax or IFN-γ^−/−^ CAR T-vax (*n*=5), shown are tumor antigen (ZsGreen) uptake by intratumoral CD103^+^ DCs (G) and CD11b^+^ DCs (H). (I-J) IFN-γ ELISPOT (I) and tumor growth (J) in mice (n = 5) treated with WT or IFNGR1^−/−^ CAR T ± vax. (K-N) IFN-γ ELISPOT. (K) WT vs. CD11c-specific IFNGR1 KO tumor-bearing mice (n=5) following WT CAR T ± vax. (L) Tumor-bearing mice (n=5) following WT CAR T or WT CAR T-vax + anti-IFN-γ or anti-IL12(p70). (M) Tumor-bearing WT mice (n=5) following WT or IL12rb2^−/−^ CAR T-vax therapy or in IL12p40^−/−^ mice following WT CAR T-vax. (N) Tumor-bearing WT mice (n=7) following WT CAR T-vax or IFNGR1^−/−^ CAR T ± vax. (O) Tumor growth in mice (n=5–7) left untreated or treated with WT or IFNGR1^−/−^ CAR T ± vax. All mice except those in G-H bear EGFRvIII^+^CT-2A tumors. Error bars are mean ± 95% CI, ***, p<0.001; **, p<0.01; *, p<0.05; ns, not significant by Student’s *t*-test for B and E-F, by one-way ANOVA with Tukey’s post-test for A, C-D, G-I and K-N, by two-way ANOVA with Tukey’s post-test for J and O.

Autocrine signaling from IFN-γ has been found to support the cytotoxicity of conventional T-cells^39^. To test if IFN-γ also promotes CAR T killing in a similar manner, we evaluated the cytotoxicity of IFN-γ^−/−^ and IFNGR1^−/−^ CAR T-cells against EGFRvIII^+^CT-2A cells *in vitro* and found that lack of IFN-γ or IFNGR1 expression by the CAR T-cells reduced cytotoxicity by ~50% (Figure 6A). Consistent with this finding, vaccine-boosted CAR-T with elevated IFN-γ expression also exhibited increased granzyme B levels in tumors (Figure S6A) and tumor cells exhibited increased signatures of immunogenic cell death, such as upregulated cell surface calreticulin expression (Figure S6B).

We next examined the DC and macrophage compartment of treated tumors, as tumor antigen released by CAR T-mediated tumor killing must be acquired by antigen presenting cells to drive T-cell priming. Vaccine boosting of CAR-T led to substantial increases in macrophages and multiple DC populations infiltrating treated tumors, including plasmacytoid DCs (pDCs), CD8^+^ DCs, CD103^+^ cDC1s (10-fold increase), and CD11b^+^ cDC2s (11-fold increase) (Figure 6B). Intratumoral macrophages also showed a shift in phenotype with upregulation of costimulatory receptors and a reduction in CD206^+^ macrophages (Figure S6C–E). However, AS induced by CAR T-vax treatment was greatly reduced in *Batf3*^−/−^ animals lacking cross-presenting DCs^40,41^ (Figure 6C), and hence we focused our attention on the DC compartment. DC recruitment to tumors relies on chemokines such as CCL3, CCL4 and CCL5^42,43^, and intratumoral expression of these chemokines was reduced when treating with IFN-γ^−/−^ CAR T-cells (Figure 6D). Ki67 expression was upregulated in CD11b^+^ and CD103^+^ DCs, suggesting a role for local expansion of intratumoral DCs in addition to recruitment from the circulation (Figure 6E). Using an EGFRvIII^+^CT-2A tumor line expressing ZsGreen as a traceable antigen, we found that vaccine boosting triggered DC activation as evidenced by upregulation of the lymph node homing marker CCR7, costimulatory receptors, and MHC-II (Figure 6F, S6F–K), and increased tumor antigen uptake by both cDC1 and cDC2 populations (Figure 6G–H). Consistent with the observed loss of AS with IFN-γ-deficient CAR T-cells, DC activation and tumor antigen uptake were lost if treatment was applied using IFN-γ^−/−^ CAR T-cells (Figure S6H–K). We also assayed for potential changes in the tumor vasculature following CAR T-vax treatment, but found it was not significantly affected (Figure S6L–N). Thus vaccine-boosting CAR T-cells amplified multiple prerequisite steps for antigen spreading.

Our *in vitro* analysis suggested a role for autocrine CAR T-cell-derived IFN-γ in sustaining CAR T cytotoxicity, but the target cells responding to IFN-γ *in vivo* remained unclear. We first tested if host cells were important responders, by transferring CAR T-cells into tumor-bearing WT or IFNGR1^−/−^ mice, followed by vaccine boosting. Endogenous T-cell priming and tumor control were completely lost in IFNGR1^−/−^ mice (Figure 6I–J). Next, we generated mice with specific deletion of IFNGR1 in CD11c^+^ DCs by crossing CD11c-cre and IFNGR-floxed animals to generate CD11c^ΔIFNGR1^ mice. As shown in Figure 6K, CAR T-vax treatment of tumor-bearing CD11c^ΔIFNGR1^ mice led to reduced but not fully ablated endogenous T-cell priming, suggesting that DCs are important responders but not the sole host cell population stimulated by IFN-γ. Activation of dendritic cells by T-cell-derived IFN-γ has been shown to trigger production of IL-12 by DCs, which in turn acts as positive feedback signal reinforcing T-cell IFN-γ expression and cytotoxic activity during checkpoint blockade immunotherapy^44^. Strikingly, antibody-mediated neutralization of IL-12 during CAR T-vax therapy or treatment of IL-12-deficient mice eliminated antigen spreading comparably to IFN-γ blockade (Figure 6L–M). The CAR T-cells themselves are important responders to IL-12, as therapy with IL-12Rb2^−/−^ CAR T-cells elicited nearly baseline endogenous T-cell priming in 4 of 5 animals (Figure 6M).

IL-12 drives sustained/elevated autocrine IFN-γ expression by T-cells. *In vivo*, vaccine-boosted IFNGR1-deficient CAR T-cells showed reduced production of IFN-γ, granzyme B and a trend toward reduced levels of TNF-α (Figure S7A–D). Blunted effector functions of IFNGR1-deficient CAR T-cells correlated with reduced induction of immunogenic cell death markers on tumor cells (Figure S7E), decreased tumor antigen uptake by intratumoral DCs (Figure S7F–G), and reduced tumor antigen acquisition by lymph node-resident CD8α^+^ cDC1 (Figure S7H); tumor antigen uptake by LN cDC2 was low and unaffected (Figure S7I). These changes in CAR-T function, tumor killing, and tumor antigen release correlated with complete loss of endogenous T-cell priming and tumor control when tumor-bearing animals were treated with CAR T-vax therapy using IFNGR1^−/−^ CAR T-cells (Figure 6N–O). Altogether, vaccine boosting enables CAR T-cells to sustain cytotoxicity in the TME and drive key events required for antigen spreading, dependent both on the ability of host DCs and the CAR T-cells themselves to respond IFN-γ.

### Robust IFN-γ production is essential for CAR T-vax therapy to control tumors with pre-existing antigen heterogeneity

**Figure. 7.**
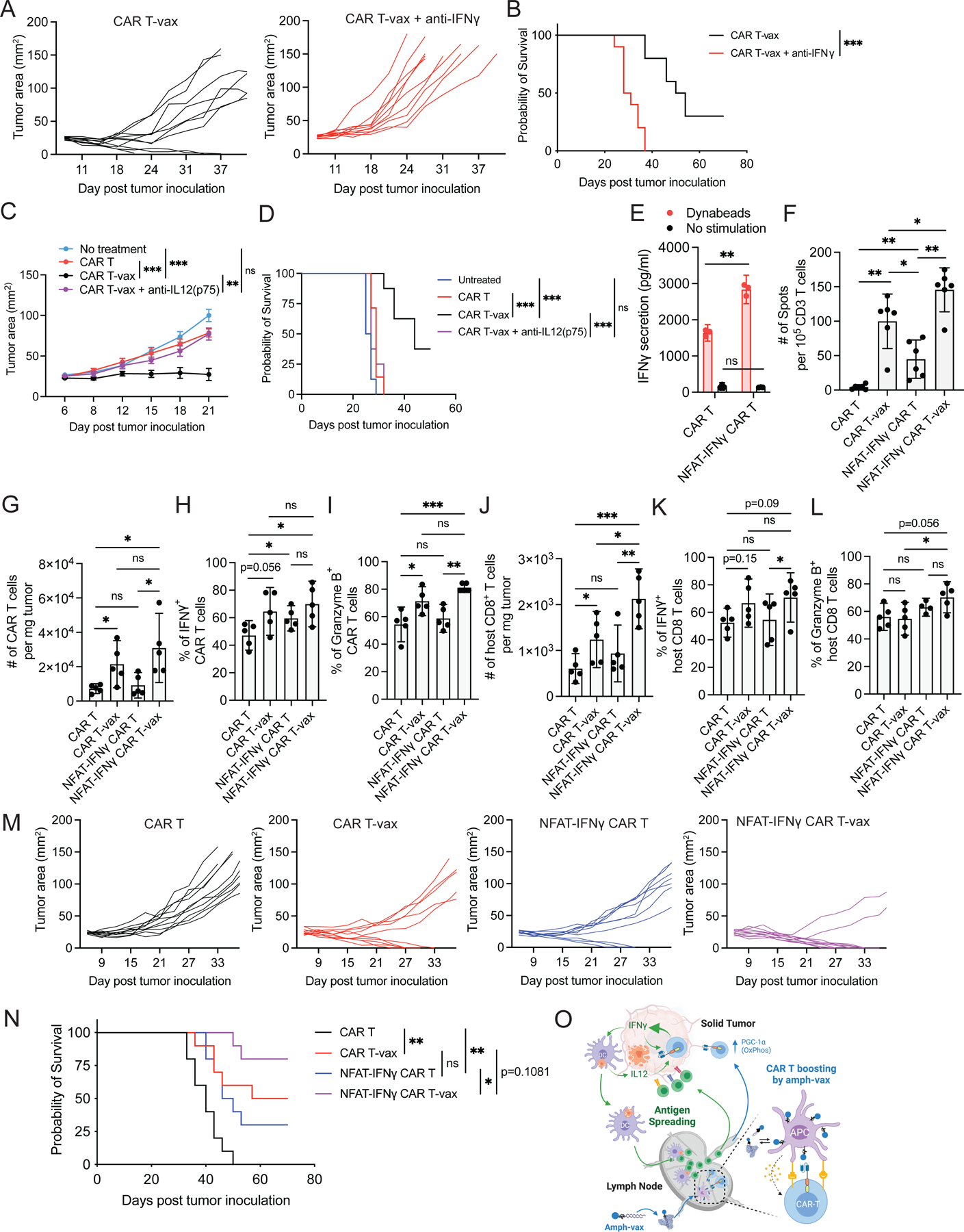
Engineering CAR T-cells for increased IFN-γ expression synergizes with vaccine boosting to enhance antigen spreading and rejection of solid tumors with pre-existing antigen heterogeneity. (A-D). Heterogenous CT-2A tumors were established in C57BL/6 mice. (A)Tumor growth and (B)survival of mice (n=10) after treatment with WT CAR T-vax therapy ± anti-IFN-γ. (C)Tumor growth and (D)survival of mice (n=8) left untreated, receiving WT CAR T, or WT CAR T-vax ± anti-IL12 (p75). (E) IFN-γ secretion from WT or NFAT-IFN-γ CAR T-cells ± anti-CD3/CD28 beads (*n*=3). (F) IFN-γ ELISPOT. EGFRvIII^+^ CT-2A tumor-bearing mice (n=6) treated with WT or NFAT-IFN-γ CAR T ± vax. (G-L) Mice bearing heterogenous CT-2A tumors (n=5) treated with WT or NFAT-IFN-γ CAR T ± vax therapy. Enumeration of CAR T (G) and endogenous CD8^+^ T cells (J) infiltrated into tumors as well as the expression of IFN-γ (H for CAR T, K for host CD8 T) and granzyme B (I for CAR T, L for host CD8 T). (M-N) Tumor growth (M) and overall survival (N) of mice bearing heterogenous CT-2A tumors (n=10) treated with WT or NFAT-IFN-γ CAR T ± vax. (O) Schematic overview of CAR T-vax therapy triggered antigen spreading. Created with BioRender.com. Heterogenous CT-2A tumors are EGFRvIII^+^:EGFRvIII^−^ cells mixed at 80:20 ratio. Error bars are mean ± 95% CI. ***, p<0.001; **, p<0.01; *, p<0.05; ns not significant by one-way ANOVA with Tukey’s post-test for E-L, by two-way ANOVA with Tukey’s post-test for C, and Log-rank (Mantel-Cox) test for B, D and N.

Based on our collective mechanistic findings regarding the importance of IFN-γ in AS, we finally assessed the role of IFN-γ in promoting control of antigenically heterogeneous tumors. Using mixed tumors comprising 80% EGFRvIII^+^ and 20% EGFRvIII^−^ tumor cells, CAR T-vax therapy in the presence of IFN-γ blockade led to loss of survival extension and elicited no complete responses (Figure 7A–B); similar results were obtained when IL-12 was blocked (Figure 7C–D). We hypothesized that enforced expression of IFN-γ might further enhance endogenous T-cell priming elicited by CAR T-vax therapy. To test this idea, we transduced CAR T-cells with retroviral constructs bearing an NFAT-driven IFN-γ expression cassette, to obtain elevated IFN-γ production following CAR activation^45^. We confirmed that NFAT-IFN-γ CAR T-cells produced nearly twice as much of IFN-γ as WT CAR T-cells upon stimulation *in vitro* (Figure 7E). Non-vaccine boosted NFAT-IFN-γ CAR T therapy elicited a significant level of endogenous T-cell priming, consistent with a critical role for sustained CAR T-cell IFN-γ in AS generally (Figure 7F). AS was further increased when NFAT-IFN-γ CAR T were used in combination with vaccine boosting, reaching 50% higher levels than treatment with WT CAR T-cells (Figure 7F). Vaccine boosting of NFAT-IFN-γ CAR T-cells led to slight trends toward increased CAR T-cell numbers in the tumor and increased IFN-γ and granzyme expression, but these did not reach statistical significance (Figure 7G–I). By contrast, endogenous T cell infiltration and granzyme expression were enhanced for NFAT-IFN-γ CAR T-vax therapy compared to CAR T-vax treatment, and IFN-γ showed a trend toward increased expression (Figure 7J–L). Vaccine-boosted WT CAR T-cells were able to reject 25–50% of 80:20 EGFRvIII^+^:EGFRvIII^−^ mixed tumors (Figure 7A–B, M–N). NFAT-IFN-γ CAR T-cells achieved similar complete response rates in the absence of vaccine boosting, and strikingly, this complete response rate increased to 80% when vaccine boosting was added to the treatment (Figure 7M–N). Importantly, vaccine boosting of NFAT-IFN-γ CAR T-cells was accompanied by mild elevations in systemic IFN-γ following the first vaccine boost, and only mild transient weight loss in animals that rapidly recovered after each vaccine boost (Figure S7J–K). Thus, strategies to enhance IFN-γ production and favorable CAR T-cell metabolism appear promising to increase the efficacy of CAR T-cell therapy against antigenically heterogenous solid tumors.

## Discussion

Antigenic heterogeneity and antigen loss play important roles in tumor escape from immune surveillance and resistance to CAR-T therapies^46–48^. The induction of antigen spreading by CAR-T therapy could address this challenge, but evidence for AS during ACT in humans remains limited. Preclinical studies using combination therapies or CAR T-cells transduced with one or more supporting genes have reported induction of AS, but mechanisms governing these responses remain poorly understood. Here we found that T-cells bearing second-generation CARs, which receive *in vivo* restimulation via a vaccine activating the CAR in lymph nodes, are capable of promoting robust host CD4^+^ and CD8^+^ T-cell responses against non-CAR-related tumor antigens. This endogenous T-cell response has significant consequences for the outcome of CAR T therapy: (1) long-term tumor regressions and complete responses are achieved against tumors that otherwise undergo antigen loss-based relapse; (2) control of antigenically heterogeneous tumors can be achieved; and (3) long term protection against tumor rechallenge is achieved.

Mechanistically, we found that enhanced production of IFN-γ by vaccine-boosted CAR T-cells was a major contributor to antigen spreading. In natural immune responses, IFN-γ promotes the activation of both innate and adaptive immunity^49^, maintenance of T-cell cytotoxicity and mobility^39^, polarization of T helper cells to Th1 cells^50^, reduction of Treg-mediated suppression^51^ and sensitization of tumors to T-cell-mediated cytotoxicity^50^. However, the role of IFN-γ in the function of CAR T-cells remains poorly defined. Recently, IFN-γ was shown to regulate the expression of cell adhesion molecules on solid tumor cells, but not leukemic cells, and subsequently enhance CAR T-cell cytotoxicity by stabilizing CAR T-tumor cell engagement^52^. Alizadeh et al. have also demonstrated that CAR T-cell-derived IFN-γ can promote recruitment of endogenous immune cells to tumors and shift the phenotype of intratumoral myeloid cells toward anti-tumor phenotypes^20^. Here, although both host T cells and CAR-T cells are IFN-γ producers in the TME, we found that IFN-γ production by CAR T-cells was most critical to enable an antigen spreading response. IFN-γ sustained high levels of cytotoxicity and effector cytokine expression in vaccine-boosted CAR T-cells in a cell-intrinsic manner. These enhanced CAR T-cell effector functions in turn correlated with increased expression of DC-recruiting chemokines in tumors, increased DC infiltration, tumor antigen uptake, and activation of intratumoral DCs. These effects of CAR T-derived IFN-γ were propagated via a positive feedback loop involving DC-derived IL-12. Such IFN-γ-IL-12 crosstalk has proven to underlie a number of successful immunotherapies, including checkpoint blockade therapy^44^ and CAR T-cell therapy in lymphoma^53^. Our data do not exclude potential contributions of other cytokines or immune cell types, such as tumor-resident macrophages, which might also play a role in the endogenous immune response^20^.

IFN-γ production is tightly regulated at both the transcriptional level by transcription factors (TFs)^54,55^ including CREB, AP-1, T-bet, NFAT, and at the post-transcriptional level by various miRNAs, ARE or GAPDH binding to its 3’UTR^56,57^. Although IFN-γ synthesis has been proposed to be predominantly associated with glycolysis due to its regulation by GAPDH^56^, both glycolysis and oxidative phosphorylation have been shown to control IFN-γ production in NK cells^58,59^, consistent with previous reports that elevated OXPHOS and mitochondria integrity was required to support IFN-γ production^58,60,61^. These findings align with our observation that genetic deletion of PGC-1α, a key transcription factor regulating OXPHOS, resulted in reduced expression of IFN-γ and a significant reduction in AS. OXPHOS is often an important feature of memory-like T-cells^62^, and enforced expression of PGC-1α endows T-cells with superior anti-tumor activity^63^. The extent to which other metabolic pathways and which gene(s), including GAPDH, are responsible for IFN-γ production by vaccine-boosted intratumoral CAR-T cells will require future investigation.

In summary, we have shown that vaccine boosting through the chimeric receptor triggers markedly enhanced CAR-T polyfunctionality and metabolic reprogramming (Figure 7O). Vaccine-boosted CAR-T cells trigger robust recruitment and activation of DCs in the tumor, which in turn secrete IL-12 that, together with the autocrine effect of IFN-γ, enhances CAR T-cell anti-tumor activity (Figure 7O), leading to pronounced endogenous T-cell priming and induction of enhanced effector programs in endogenous T-cells that infiltrate tumors. In our models, we find that such antigen spreading is critical for avoidance of antigen loss-mediated tumor escape and control of antigenically heterogenous tumors. As few solid tumors express target antigens on >90% of tumor cells, these findings provide guidance for engineering more effective CAR-T therapies. Notably, vaccines for CAR T-cells are already being explored clinically^64–66^, suggesting this approach can be readily translated to CAR-T cell clinical trials.

### Limitations of the Study

We elected to use a glioblastoma model (CT-2A) transduced to express the GBM mutant antigen EGFRvIII implanted in the flank for many of our studies, which could be mixed with parental EGFRvIII-CT-2A cells in distinct ratios to quantify the impact of antigen spreading. This provided a model system where antigen heterogeneity was well defined and allowed experimental throughput for mechanistic studies that would not be possible in an orthotopic GBM model, but does not model the orthotopic GBM microenvironment or natural EGFRvIII expression heterogeneity. We did however evaluate CAR T-vax therapy targeting endogenous tumor-associated antigens to confirm the key findings of antigen spreading and heterogenous tumor control in a model lacking artificially introduced antigens. We also focused our studies on syngeneic mouse models, as immunodeficient mouse hosts used for preclinical human CAR T-cell therapy lack proper lymphatic and lymph node formation, which is problematic for the vaccine boosting treatment.

## STAR Methods

### RESOURCE AVAILABILITY

#### Lead Contact

Further information and requests for resources and reagents should be directed to and will be fulfilled by the lead contact Darrell Irvine (djirvine@mit.edu).

#### Materials Availability

New plasmids from this paper are available from the lead contact upon request.

#### Data and Code Availability

- Bulk-RNA seq and single cell RNA-seq data have been deposited at GEO (GSE211938, GSE212453) and are publicly available as of the date of publication.
- Codes used to process and analyze single-cell RNA-seq data are available at github.com/duncanmorgan/CAR_AgSpreading or Zenodo (10.5281/zenodo.7939518).
- Any additional information required to reanalyze the data reported in this paper is available from the Lead Contact upon request.

### EXPERIMENTAL MODEL AND STUDY PARTICIPANT DETAILS

#### Cell line and Constructs

B16F10 and 293 phoenix cells were obtained from ATCC. B16F10-OVA cells were a gift from Dr. Glen Dranoff at the Dana Farber Cancer Institute. TRP1^−/−^ B16F10 cells were generated previously using CRIPSR ^70^. The mouse CT-2A glioma cell line was kindly provided by Dr. Thomas Seyfried from Boston College. mEGFRvIII-expressing CT-2A cells were generated by lentiviral transduction of CT-2A cells with a murine version of EGFRvIII and stably selected with puromycin. ZsGreen^+^ mEGFRvIII-CT-2A cells were generated by transducing mEGFRvIII-CT-2A cells with ZsGreen-expressing lentivirus and subsequent flow cytometry enrichment. mEGFRvIII-CT-2A-OVA cells were generated by transducing mEGFRvIII-CT-2A cells with PLKO-based lentivirus expressing Thy1.1-IRES-OVA (aa251–388). MHCII^+^ CT-2A cells were generated by transducing CT-2A cells with lentivirus expressing CIITA (Class II Major Histocompatibility Complex Transactivator).

#### Animals

Female mice (6–8 week old) were used for all studies. Wildtype female C57BL/6J mice (B6, CD45.2^+^), CD45.1^+^ congenic mice (B6.SJL-*Ptprc*^*a*^ *Pepc*^*b*^/BoyJ), Rag1^−/−^ (B6.129S7-*Rag1*^*tm1Mom*^/J, B6 background), IFN-γ^−/−^ (B6.129S7-*Ifng*^*tm1Ts*^/J, congenic with B6, backcrossed for at least 8 generations ), IFNGR1^−/−^ (B6.129S7-*Ifngr1*^*tm1Agt*^/J, B6 background), *Batf3*^*−/−*^ (B6.129S(C)-*Batf3*^*tm1Kmm*^/J, B6 background), PGC-1α-flox (B6N.129(FVB)-*Ppargc1a*^*tm2.1Brsp*^/J), LCK-cre (B6.Cg-Tg(Lck-cre)548Jxm/J, Hemizygous), IL12rb2^−/−^ (B6;129S1-*Il12rb2*^*tm1Jm*^/J, B6 background), IL12p40^−/−^ (B6.129S1-*Il12b*^*tm1Jm*^/J, congenic with B6, backcrossed for at least 9 generations), CD11c-cre (C57BL/6J-Tg(Itgax-cre,-EGFP)4097Ach/J, Hemizygous)，IFNGR1-flox (C57BL/6N-*Ifngr1*^*tm1.1Rds*^/J) mice and CD8α^−/−^ (B6.129S2-Cd8^atm1Mak^/J) mice were purchased from the Jackson Laboratory. To avoid neonatal lethality caused by whole body KO of PGC-1α, T cell-specific PGC-1α KO mice were created by crossing LCK-cre mice with PGC-1α-flox mice; cre^+^ F1 offspring have T cell-specific PGC-1α KO while the cre^−^ F1 offspring have a wildtype phenotype and were used as donor control T cells for Fig 3F. CD11c^ΔIFNGR1^ mice were generated by crossing CD11c-cre mice with IFNGR1-flox mice, cre^+^ F1 offspring are IFNGR1-deficient in CD11c^+^ cells while the cre^−^ F1 offspring have a wildtype phenotype and were used as control recipients in Fig. 6H. For all studies, 6–8 weeks old mice were used. All animal studies were carried out following an IACUC-approved protocol following local, state, and federal guidelines.

### METHOD DETAILS

#### Cloning and constructs

The murine EGFRvIII CAR (28z) and FITC/TA99 bispecific CAR (28z) were cloned into an MSCV retroviral vector as previously described^32^. The NFAT-IFN-γ cassette was constructed in a self-inactivating (SIN)-retroviral vector with 6xNFAT binding sites^71^ upstream of the minimal IL2 promoter driving murine IFN-γ expression.

#### Primary mouse T cell isolation and CAR T-cell production

For T cell activation, 6-well plates were pre-coated with 5 ml of anti-CD3 (0.5 μg/ml, Clone: 2C11) and anti-CD28 (5 μg/ml, Clone: 37.51) per well at 4°C for 18 hr. CD8^+^ T cells were isolated using a negative selection kit (Stem Cell Technology), and seeded onto pre-coated 6-well plates at 5 ×10^6^ cells/well in 5 ml of complete medium (RPMI + penicillin/streptomycin + 10% FBS + 1x NEAA + 1x Sodium pyruvate + 1x 2-mercaptoethanol + 1x ITS [Insulin-Transferrin-Selenium, Thermo Fisher]). Cells were cultured at 37°C for 48 hr without disturbance. Twenty-four hr before transduction, non-TC treated plates were coated with 15 μg/ml of retronectin (Clonetech). On day 2, cells were collected, counted and resuspended at 2×10^6^ cells/ml in complete medium supplemented with 20 μg/ml of polybrene and 40 IU/mL of mIL-2. Retronectin-coated plates were blocked with 0.05% FBS containing PBS for 30 min before use. 1 ml of virus supernatant was first added into each well of the blocked retronectin plate, then 1 mL of the above cell suspension was added and mixed well by gentle shaking to reach the working concentration of polybrene at 10 μg/ml and mIL-2 at 20 IU/ml. Spin infection was carried out at 2000×*g* for 120 min at 32°C. Plates were then carefully transferred to an incubator and maintained overnight. On day 3, plates were briefly centrifuged at 1,000×*g* for 1 min, and virus-containing supernatants were carefully removed. 3 mL of fresh complete medium containing 20IU/ml of mIL-2 were then added into each well. Cells were passaged 1:2 every 12 hr with fresh complete medium containing 20IU/mL of mIL-2. Transduction efficiency was evaluated by surface staining of a c-Myc tag included in the CAR construct^32^ using an anti-Myc antibody (Cell signaling, Clone:9B11) ~30 hr after transduction. If needed, CAR T-cells on day 3, after flow cytometry analysis of virus transduction, could be frozen down and stored for assays at a later time. For in vivo experiments, CAR T-cells were used on day 4. For *in vitro* experiments, CAR T-cells were cultured till day 5.

#### Virus production and transduction evaluation

For optimal retrovirus production, 293 phoenix cells were cultured till 80% confluence, then split at 1:2 for further expansion. 24 hr later, 5.6×10^6^ cells were seeded in a 10 cm dish and cultured for 16 hr till the confluency reached 70%. 30 min – 1 hr before transfection, each 10 cm dish was replenished with 10 ml pre-warmed medium. Transfection was carried out using the calcium phosphate method following the manufacturer’s protocol (Clonetech). Briefly, for each transfection, 18 μg of plasmid (16.2 μg of CAR plasmid plus 1.8 μg of Eco packaging plasmid) was added to 610 μl of ddH_2_O, followed by addition of 87 μl of 2 M CaCl_2_. 700 μl of 2x HBS was then added in a dropwise manner with gentle vortexing. After a 10 min incubation at 25°C, the transfection mixture was gently added to phoenix cells. After 30 min incubation at 37°C, the plate was checked for the formation of fine particles, as a sign of successful transfection. The next day, old medium was removed and replenished with 8 ml of pre-warmed medium without disturbing the cells. Virus-containing supernatant was collected 36 hr later and passed through a 0.45 um filter to remove cell debris, designated as the “24hr” batch. Dishes were refilled with 10ml of fresh medium and cultured for another 24 hr to collect viruses again, designated as the “48hr” batch, this process can be repeated for another two days to collect a “72hr” batch and “96hr” batch. All virus supernatant was aliquoted and stored at −80°C. Virus transduction rate was evaluated in a 12-well format by mixing 0.5 million activated T cells with 0.5ml of viruses from each batch. Plate coating, spin infection and FACS analysis of CAR expression were carried out as described above. In the majority of experiments, the “48hr” and “72hr” batches yielded viruses that transduced T cells at 90–95% efficiency, the “24hr” and “96hr” batch viruses led to >80% transduction. Only viruses with >90% transduction rate were used for animal studies.

#### Amphiphile-ligand production and vaccination

DSPE-PEG-FITC was purchased from Avanti. Amph-pepvIII was produced as previously described^72^. Briefly, pepvIII peptides (LEEKKGNYVVTDHC) were dissolved in dimethylformamide at 10 mg/mL and mixed with 2.5 equivalents of 1,2-distearoyl-*sn*-glycero-3-phosphoethanolamine-N-[maleimide(polyethylene glycol)-2000] (Laysan Bio, Inc), 1 equivalent of tris(2-carboxyethyl)phosphine hydrochloride (Sigma), and a catalytic amount (~10ul) of triethylamine. The mixture was agitated at 25°C for 24 hr. Unconjugated peptides were removed using HPLC. Amph-pepvIII concentration was determined using nanodrop. The resulting products were lyophilized, re-dissolved in PBS and stored at −20°C. For vaccination, unless otherwise stated, mice received weekly s.c injection of 10 μg peptide equivalent of amph-pepvIII mixed with 25 μg of Cyclic-di-GMP (CDG, Invivogen) in 100 μl 1x PBS, administered 50 μl to each side at the tail base. To compare the effect of adjuvants on vaccination, 1.24 nmol lipo-CpG^72^ or 10μg R848 (TLR7/8 agonist, Resiquimod [Invivogen]) was used per mouse.

#### ELISPOT

To evaluate epitope spreading, the spleen was harvested from individual mice for total T cell isolation using a CD3^+^ T cell isolation kit (Stem Cell Technology). For most experiments, CAR T-cells were prepared using T cells isolated from CD45.1^+^ mice, transferred into tumor-bearing CD45.2^+^ recipients, enabling magnetic depletion of adoptively transferred CAR T-cells during endogenous T cell isolation using negative selection. For this purpose, anti-CD45.1 antibody (Clone A20, Stem Cell Technology) were added to whole splenocytes at 1ug/ml together with the T cell isolation cocktail. The day before T cell isolation, 2×10^6^ tumor cells (CT-2A, MHCII^+^CT-2A or B16F10 cells) were seeded in a T75 flask in the presence of 100 IU of murine IFN-γ [PeproTech] and subjected to 120Gy of irradiation the next morning. Tumor cells were then trypsinized into single cell suspension using TrypLE Express (Gibco) to avoid removal of surface proteins and washed twice with 1x PBS to remove residual IFN-γ. 4×10^5^ CD3^+^ T cells were mixed with 25,000 irradiated tumor cells in 200 µL complete medium and seeded in a 96-well ELISPOT plate (BD) that was pre-coated with IFN-γ capture antibody (BD IFN-γ ELISPOT kit). Plates were wrapped in foil and cultured for 24hr in 37°C incubator, then developed according to the manufacturer’s protocol. Plates were scanned using a CTL-ImmunoSpot Plate Reader, and data were analyzed using CTL ImmunoSpot Software.

#### CAR T functionality assay

The functionality of WT, IFN-γ^−/−^, IFNGR1^−/−^ or NFAT-IFNγ CAR T-cells was assessed by co-coculturing with EGFRvIII-CT2A cells in 96-well flat-bottom plates. Unless otherwise stated, 1×10^5^ CAR T-cells were mixed with 1×10^4^ target cells in a total volume of 200 μl complete medium containing 20IU/ml of mIL-2. After 6 hr co-culture, cells were resuspended by vigorous pipetting, transferred to a U-bottom plate, and pelleted at 2,000×*g* for 5 min. The supernatant was saved for ELISA following the manufacturer’s protocol (Mouse IFN-γ Duo set, R&D systems). Cells were stained with anti-CD45 and anti-CD8α for 20 min on ice and resuspended in flow cytometry buffer with 1x SYTOX Red (Thermo Fisher) for flow analysis. Dead tumor cells were gated as CD8^−^ CD45^−^ SYTOX RED^+^ population. IFN-γ ELISAs were performed following the manufacturer’s protocol.

#### P15E antigen and Env protein detection

Env protein expression on CT-2A cell surface was monitored using flow cytometry and staining with 1E4.2.1 anti-Env antibody as previously described^67^ (Wittrup lab). The presentation of Env antigen p15E on CT-2A cells were assessed by co-culturing IFN-γ-treated CT-2A cells with a 58^−/−^ T cell hybridoma cell line expressing a p15E-specific TCR 7PPG-2 (Birnbaum lab) and monitoring T cell activation using mouse IL-2 ELISA (Invitrogen) as previously described^32,68^. A 58^−/−^hybridoma cells expressing an irrelevant 2C TCR (Birnbaum lab) were included as negative control. TC-1 cells and MC38 cells were included as negative control and positive control of ENV/p15E expression, respectively.

#### Secondary transplantation study

To evaluate the qualitative anti-tumor activity of CAR T-cells, 10 million CD45.1^+^ donor CAR T-cells were i.v. infused to lymphodepleted CD45.2^+^ recipients (500cGy sublethal irradiation) followed 24hr later by a single dose of amph-pepVIII vaccination or mock vaccination with PBS. Seven days later, mice were euthanized, and spleens were harvested and combined for each group for total T cell isolation using a modified pan-T cell negative selection protocol. Briefly, total splenocytes were stained with a pan-T cell isolation cocktail (Stem Cell Technology) plus 1:500 dilution of biotinylated anti-CD45.2 antibody (Clone 104, 0.5mg/ml, Stem Cell Technology). Negative selection was performed following the same downstream procedures as listed in the manufacture’s protocol to obtain untouched vaccine-boosted CD45.1 CAR T-cells. Immediately after isolation, ~8×10^6^ CD45.1 T cells from either mock or vaccine-treated groups were adoptively transferred to secondary recipients bearing ~25 mm^2^ EGFRvIII-CT-2A tumors that had been lymphodepleted the day before, followed by periodic monitoring of tumor growth and animal survival. Note: throughout this study, the retroviral transduction efficiency and subsequent CAR+ T cells was constantly >90%, therefore, the total number of transferred CAR+ CD45.1 T cells are ~7×10^6^.

#### Luminex assay

EGFRvIII-CT-2A tumor-bearing C57BL/6 mice received lymphodepletion followed by adoptive transfer of either WT or IFN-γ^−/−^ CAR T-cells plus a single dose of vaccination. Mice were euthanized and tumors isolated at day 7 post vaccination. Tumors were weighted, cut using a razor blade into small pieces and dounced to generate tumor homogenate in tissue protein extraction buffer (T-PERTM, Thermo Fisher Scientific, cat. no. 78510) in the presence of 1% proteinase and phosphatase inhibitors (Thermo Fisher Scientific, cat. no. 78442). The lysates were incubated at 4°C for 30 min with slow rotation followed by top-speed centrifugation to remove debris. The supernatants were transferred to a clean tube and stored at −80°C. Part of the samples were subjected to Luminex analysis using a Mouse Cytokine 32-Plex panel analysis at Eve Technology.

#### Tumor sectioning and vasculature staining

C57BL/6 mice bearing EGFRvIII^+^CT-2A tumors received lymphodepletion (LD), followed by no treatment, or were treated with WT CAR T or IFNγ^−/−^ CAR T in the presence or absence of vaccination as in Fig 1C. Seven days post vaccination, mice were euthanized 5 minutes after intravenous injection with 0.2 mg Hoechst 33342 (Thermofisher) and 0.2 mg Dextran Tetramethylrhodamine 70,000 MW (Thermofisher). Tumors were harvested and fixed with 4% PFA at 4 °C for 18 h. Next, isolated tumors were washed in PBS and embedded in 3% (wt/vol) low-melting agarose at 37 °C. The agarose was allowed to solidify on ice for 15 min before sectioning on a vibratome (Leica VT1000S). 150-μm tissue sections were incubated with Fc Receptor Blocker (Innovex Bioscience) for 30 minutes and then blocked with 2% bovine serum albumin in PBS for 1 h at room temperature. Tumor vessel staining with primary antibodies (1:100) was performed overnight at 4 °C in blocking buffer using Alexa Fluor^®^ 647 anti-mouse CD31 Antibody (BioLegend, #102516). After three washes with PBS, the sections were mounted onto glass slides using mounting media (ProLong Diamond Antifade Mountant, Thermo Fisher Scientific). Images were acquired using a Leica SP8 laser-scanning confocal microscope with a 25× objective. Image processing was performed with Fiji ^69^ and Imaris v10. The surface tracing algorithm was used to trace and mask the anti-CD31 channel. Total vessel diameter and vessel volume was calculated using the Filament tracing algorithm on the masked anti-CD31 channel. Hoechst area were calculated using the Analyze particles function in Fiji as % of tumor area perfused.

#### Bulk RNA-sequencing for CAR T characterization

EGFRvIII-CT-2A tumor-bearing CD45.2^+^ mice were treated with CD45.1^+^ CAR T-cells and mock (PBS) or amph-pepVIII vaccination. 7 days later, mice were euthanized to harvest spleens and tumors. Total splenic T cells were isolated using the pan-T cell isolation kit and stained with anti-CD8α, anti-CD4, anti-CD45.1 and 7AAD for flow sorting. 5×10^4^ CD45.1^+^ CAR T-cells were directly sorted into Trizol. For intratumoral CAR T isolation, tumors were cut into 1–2 mm^2^ pieces using razor blades, placed in 1.5ml or 5ml tubes (depending on tumor size) and digested (2 mg/ml Collagenase IV [Worthington], 0.1mg/ml of DNAse I [Sigma], and 10% of TrypLE [Thermo Fisher] in 1xRPMI) for 20 min on a rotator at 37°C. Digested tumors were then mushed through a 70um cell strainer using a blunt non-rubber end of the a 1ml syringe plunger, washed 1x with 1xRPMI. Intratumoral T cells were enriched using mouse CD4/CD8 (TIL) MicroBeads (Miltenyi), stained, and sorted into Trizol as above. The total number of sorted CD45.1^+^ CAR T-cells from tumors ranged from 6×10^3^ to 5×10^4^ per sample. Total RNA was isolated using the RNeasy Micro kit (Qiagen). Samples were submitted to the BioMicro center at MIT for library construction and sequencing. Bulk RNA-sequencing data was analyzed with the help from the bioinformatics core at the Koch Institute. Briefly, paired-end RNA-seq data was used to quantify transcripts from the mm10 mouse assembly with the Ensembl version 100 annotation using Salmon version 1.2.1^73^. Gene level summaries for were prepared using tximport version 1.16.0^74^. running under R version 4.0.0 (https://www.R-project.org). Differential expression analysis was performed using DESeq2 version 1.28.1^75,76^ and differentially expressed genes were defined as those having an absolute apeglm^77^ log2 fold change greater than 1 and an adjusted p-value less than 0.05. Data parsing and some visualizations were carried out using Tibco Spotfire Analyst 7.6.1. Mouse genes were mapped to human orthologs using Mouse Genome Informatics (http://www.informatics.jax.org/) orthology report. Preranked GSEA^78^ was run using javaGSEA version 4.0.3 for gene sets from MSigDB version 7.1^79^. Preranked GSEA for custom mouse gene sets was run with javaGSEA version 4.1.0

#### Seq-Well Single cell RNA-sequencing to profile AS in intratumoral T cells

Tumors were digested and tumor-infiltrating lymphocytes enriched as described above for bulk RNA-seq. Enriched TILs from individual mice were first labeled with Total-seq A anti-mouse hashing antibodies (BioLegend) and washed 2x in flow cytometry buffer. Samples from the same group were then combined and stained with the same surface staining antibody cocktail. Endogenous CD45.2^+^ CD4 and CD8 T cells were sorted collectively into 1x RPMI +10%FBS, 2–5×10^4^ total T cells were obtained for each group. Cells were pelleted at 1000×g for 5min, resuspended in 1xRPMI at 20,000 cells per 200μl and then processed for scRNA-seq using the Seq-Well platform with second strand chemistry, as previously described^80^. Whole transcriptome libraries were barcoded and amplified using the Nextera XT kit (Illumina) and were sequenced on a Novaseq 6000 (Illumina). Hashtag oligo libraries were amplified as described previously^81^ and were sequenced on a Nextseq 550.

#### Processing of single cell hashing data

Cell hashing data was aligned to HTO barcodes using CITE-seq-Count v1.4.2 (https://zenodo.org/badge/latestdoi/99617772). To establish thresholds for positivity for each HTO barcode, we first performed centered log-ratio normalization of the HTO matrix and then performed k-medoids clustering with k=5 (one for each HTO). This produced consistently five clusters, each dominated by one of the 5 barcodes. For each cluster, we first identified the HTO barcode that was dominant in that cluster. We then considered the threshold to be the lowest value for that HTO barcode among the cells classified in that cluster. To account for the scenario in which this value was substantially lower than the rest of the values in the cluster, we used Grubbs’ test to determine whether this threshold was statistically an outlier relative to the rest of the cluster. If the lower bound was determined to be an outlier at p=0.05, it was removed from the cluster, and the next lowest value was used as the new threshold. This procedure was iteratively applied until the lowest value in the cluster was no longer considered an outlier at p=0.05. Cells were then determined to be “positive” or “negative” for each HTO barcode based on these thresholds. HTO thresholds were examined and manually adjusted if necessary. Cells that were positive for multiple HTOs (doublets) or were negative for all HTOs were excluded from downstream analysis. To account for differences in sequencing depth between samples, these steps were performed separately for each Seq-Well array that was processed.

#### scRNA-seq data processing and visualization

Raw read processing of scRNA-seq reads was performed as previously described^82^. Briefly, reads were aligned to the mm10 reference genome and collapsed by cell barcode and unique molecular identifier (UMI). Then, cells with less than 500 unique genes detected and genes detected in fewer than 5 cells were filtered out, and the data for each cell was log-normalized to account for library size. Genes with log-mean expression values greater than 0.1 and a dispersion of greater than 1 were selected as variable genes, and the ScaleData function in Seurat was used to regress out the number of UMI and percentage of mitochondrial genes in each cell. Principal components analysis was performed. The number of principal components used for visualization was determined by examination of the elbow plot, and two-dimensional embeddings were generated using uniform manifold approximation and projection (UMAP). Clusters were determined using Louvain clustering, as implemented in the FindClusters function in Seurat, and clusters that contained activated T cells were selected for further analysis. These cells were reprocessed with the same processing and clustering steps described above. DEG analysis was performed for each cluster and between indicated cell populations using the FindMarkers function.

#### qPCR to validate differentially expressed genes

Splenic CD45.1^+^ CAR T-cells isolated from CAR T only or CAR T-vax treated mice 7 days post the first vaccine. Briefly, total T cells were first enriched using pan T cell isolation kit followed by staining of CD8, Myc and CD45.1 surface markers. CD45.1^+^ Myc^+^ cells were FACS-sorted into Trizol. The total number of sorted CD45.1^+^ CAR T-cells from spleens range from 4×10^4^ – 6×10^4^ per sample. Total RNA was isolated using the RNeasy Micro kit (Qiagen) and subjected to cDNA synthesis using iScript Reverse Transcription Supermix in 20ul reaction. qPCR primers were designed and qPCR reactions as carried out previously described ^83^. Actin was used as the internal control, and genes with CT value lower than 32 were considered as detectable and the corresponding sample was included for statistically analysis.

#### Paired single-cell TCR sequencing and analysis

Paired TCR sequencing and read alignment was performed as previously described^84^. Briefly, whole transcriptome amplification product from each single-cell library was enriched for TCR transcripts using biotinylated *Tcrb* and *Tcra* probes and magnetic streptavidin beads. The enrichment product was further amplified using V-region primers and Nextera sequencing handles, and the resulting libraries were sequenced on an Illumina Novaseq 6000. Processing of reads was performed using the Immcantation software suite^85,86^. Briefly, reads were aggregated by cell barcode and UMI, and UMI with under 5 reads were discarded. ClusterSets.py was used to divide sequences for each UMI into sets of similar sequences. Only sets of sequences that comprised greater than 90% of the sequences obtained for that UMI were considered further. Consensus sequences for each UMI were determined using the BuildConsensus.py function. Consensus sequences were then mapped against TCRV and TCRJ IMGT references sequences with IgBlast. Sequences for which a CDR3 sequence could not be unambiguously determined were discarded. UMI for consensus sequences were corrected using a directional UMI collapse, as implemented in UMI-tools^87^. TCR sequences were then mapped to single cell transcriptomes by matching cell barcodes. If multiple *Tcra* or *Tcrb* sequences were detected for a single cell barcode, then the corresponding sequence with the highest number of UMI and raw reads was retained. TCR data for p15E tetramer-sorted CD8+ T cells was obtained from Grace et al^68^. Using this data, we defined high-confidence p15E-specific Tcrb and Tcra CDR3 amino acid sequences as sequences that were detected in more than one cell and for which greater than 80% of total sequences recovered were in the tetramer-positive fraction. Using this set of sequences as a reference, we defined likely p15E-specific clonotypes in our sequencing of TIL from CAR T and CAR T-Vax treated mice as clonotypes that utilized either one of these Tcrb or Tcra amino acid sequences or utilized the Tcra motif “DYSNNRLT”, which was strongly implicated in the recognition of the p15E epitope by Grace et al. To define a cytotoxicity score for each CD8+ T cell in our single-cell sequencing data, we utilized the AddModuleScore function in Seurat using the following genes as a signature: Gzma, Gzmb, Gzmc, Gzmd, Gzme, Gzmf, Gzmg, Gzmk, Gzmm. Single T cells for which neither a Tcrb or Tcra sequence were recovered were excluded from this analysis.

#### Phenotyping of immune cells in peripheral blood, lymph nodes, and tumors

Peripheral blood (PB) was collected via retro-orbital bleeding. 50–100 μl PB (lymphodepleted mice) was processed in ACK lysis buffer twice (3–5min the 1^st^ time till all RBCs were lysed followed by centrifugation at 1000×g for 5min, decant, resuspend in 200ul ACK and spin again), immediately after spin the 2^nd^ time, instead of decanting, RBC debris from each well was carefully removed by vacuuming in a circular motion without touching the center of the pellet. Lymph nodes (LNs) were placed in a 5ml flow cytometry tube with a 70 µM cell strainer cap and smashed through with the rubber end of a 1ml syringe plunger with frequent addition of flow cytometry buffer. Dissociated LN cells were pelleted and transferred to a 96 well U-bottom plate for further analysis. EGFRvIII-CT-2A tumors from mice receiving CAR T or CAR T-vax therapy were surgically removed and weighed and dissociated into single cell suspension using enzyme digestion as described above. Single cell suspensions were obtained by passing tumors through a 70 µM cell strainer with a 1 ml syringe plunger. Cells were pelleted and resuspended with 100 μl of FACS buffer per 100 mg tumor. For immunophenotyping analysis, PBMCs or lymph node cell suspensions were pelleted in a 96 well U-bottom plate and stained with desired antibody cocktails at a 1:200 dilution for CD4^+^ T cells, CD8^+^ T cells, Tregs (FoxP3^+^), B cells (B220^+^), CD103^+^ cDC1 (CD24^+^CD11c^+^F4/80^−^CD103^+^), CD11b^+^ cDC2 (CD24^+^CD11c^+^F4/80^−^CD11b^+^), pDCs (CD24^+^CD11c^+^F4/80^−^CD317^+^), M1 (CD11b^+^F4/80^+^CD206^−^) and M2 (CD11b^+^F4/80^+^CD206^+^) macrophages.

For intracellular cytokine staining (ICS) analysis, 50–75 μl of the above tumor cell suspension was pelleted in 96 well U-bottom plates and directly resuspended in RPMI1640 with 10% FBS plus 1x Golgi plug and 1x cell stimulation cocktail (Thermo Fisher) for 6 hr at 37°C. Cells were then pelleted at 1000×g for 5 min and washed once with PBS, then stained with live/dead aqua for 15 min in the dark at 25°C. Cells were pelleted again, surface stained with desired antibody cocktail for ~20 min on ice followed by 1x wash with flow cytometry buffer. Cells were resuspended in 75 ml of BD Fix/Perm and kept at 4°C for 15 min, then washed once by directly adding 200 μl 1x Perm/Wash. The pellet was resuspended in 50 μl of cytokine antibody cocktail (IFN-γ at 1:100, TNF-α at 1:100, and granzyme B at 1:100) pre-diluted in 1x Perm/Wash buffer, 30 min on ice, then washed once with 1x Perm/Wash buffer and resuspended in 1x flow cytometry buffer for analysis immediately or kept at 4°C for FACS analysis the next day. For FoxP3 or PGC-1α staining, 50–75μl of the above cell suspension was pelleted and processed using a FoxP3 staining kit (Thermo Fisher) according to the manufacturer’s instructions.

For tetramer staining, PBMCs or tumor suspensions were stained with 50 µl of SIINFEKL-Tetramer (PE conjugate) plus Fc block at a 1:50 dilution for 30 min at room temperature in the dark, followed by mixing with a pre-made 50 μl cocktail of the remaining surface antibodies (1:50 dilution), 20min on ice kept from light. Then cells were washed twice for flow analysis.

#### CAR T-vax therapy in solid tumor models

In the EGFRvIII-CT-2A mouse glioblastoma model, unless otherwise stated, 5×10^6^ EGFRvIII-CT-2A cells were injected into the right flank of recipient mice in 50 μl saline and allowed to establish palpable tumors ~25 mm^2^ in size at day 6. Lymphodepletion was carried out using 500 cGy sublethal irradiation, mice were then randomly allocated into each group. 10×10^6^ CAR T-cells from mice with the desired background were i.v. infused via the tail vein into recipient mice followed by weekly s.c. immunization with amph-pepvIII vaccine (10 μg amph-pepvIII, 25 μg CDG in 100μl PBS) or PBS alone. For consistency, Rag1^−/−^ mice were also subjected to the same lymphodepletion preconditioning. For experiments involving cytokine blockade, unless otherwise stated, anti-IFN-γ (BioXcell) was administered i.p. at 200 μg per mouse every three days, anti-TNF-α (BioXcell) was administered i.p. at 300 μg per mouse every two days, anti-IL12(p75) was administered i.p. at 1mg per mouse for the initial dose followed by 500 μg per mouse every three days.

For the mixed tumor studies, each mouse was inoculated in the right flank with 5×10^6^ EGFRvIII-CT-2A cells and WT CT-2A cells mixed at pre-defined ratios (100:0, 90:10, 80:20, 50:50, 25:75, 0:100). 5 days later, when the tumors reached ~25mm^2^, mice were subjected to lymphodepletion and adoptive transfer of 10×10^6^ EGFRvIII CAR T-cells, followed 24hr later with weekly vaccination.

In the B16F10-based mouse melanoma model, B16F10-OVA tumors were established by s.c injection of 1×10^6^ Ova^+^B16F10 cells into the right flank of C57BL/6 recipient mice in 50 μl saline. For the mixed B16F10 tumor studies, each mouse was inoculated in the right flank with 4 ×10^5^ WT B16F10 cells and Trp1^−/−^ B16F10 cells^70^ mixed at pre-defined ratios (80:20). Mice received lymphodepletion preconditioning with 500 cGy sublethal irradiation at day 5, and the i.v. infusion of PBS, 10×10^6^ CD45.1^+^ FITC-CAR T, FITC/TRP1 bispecific CAR T-cells on day 6, followed with or without two weekly amph-FITC immunizations (10nmol amph-FITC, 25 μg CDG in 100μl PBS).

#### NFAT-IFNγ CAR-T vax toxicity analysis

Serum was collected 24 hr before and after the 1^st^ and 2^nd^ vaccination of tumor-bearing animals. Serum cytokine levels were quantified using Legendplex beads following the manufacturer’s protocol. Vaccine and CAR- T therapy-induced body weight (BW) fluctuations in each group were calculated with the following equation: [BW (Day x) / BW (Day 0)] / [BWcontrol (Day x) / BWcontrol (Day 0)].

### QUANTIFICATION AND STATISTICAL ANALYSIS

Statistical analyses were performed using GraphPad Prism 8. All values and error bars are shown as mean ± 95% CI (confidence interval). Animal survival was analyzed using Log-rank (Mantel-Cox) test. All pair-wise comparisons were analyzed by student’s t-test. Multi-group comparisons was carried out using one-way ANOVA with Tukey’s multiple comparisons test. Experiments that involved repeated measures over a time course, such as tumor growth, were analyzed using a RM (repeated measures) two-way ANOVA based on a general linear model (GLM). The RM design included factors for time, treatment and their interaction. Tukey’s multiple comparisons test was carried out for the main treatment effect. P-values are adjusted to account for multiple comparisons in both one-way ANOVA, and RM two-way ANOVA. For all animal experiments, 6–8-week-old female C57BL/6 were used. At this age, mice have developed a mature immune system, thus ideal for evaluating immunomodulating therapies. We determined the size of samples for experiments involving either quantitative or qualitative data as previously reported^88^. Based on our previous experience with the animal models and as reported by others^27,70,89^, we consider the CAR T-vax therapy as significant if it increases the survival of animals up to 100% within 4 weeks, and we need >=5 animals per group to achieve this goal with 95% confidence interval and at 80% power.

## Supplementary Material

1 Figure S1. Delineation of factors contributing to CAR T-vax therapy induced antigen spreading, related to Figure 1 (A) Schematic of the development of anti-EGFRvIII CAR and amph-pepvIII vaccine.
(B) Kinetics of the recovery of T cell and DC populations post sublethal irradiation (500 cGy) in C57BL/6 mice. Day 0 denotes the baseline level prior to irradiation. Arrow indicates day of irradiation. Shown is one representative of at least three independent experiments.
(C) Impact of tumor location and site of vaccination on the magnitude of CAR T-vax induced antigen spreading. C57BL/6 mice bearing EGFRvIII^+^CT-2A tumors received lymphodepletion (LD) followed by treatment with control (untransduced) T-cells or CAR T-vax following the same timeline as in Fig 1C. IFN-γ ELISPOT was assayed for splenic T cells isolated on day 21 and stimulated with irradiated EGFRvIII-negative CT-2A cells. Shown are representative ELISPOT well images and quantitative ELISPOT data from one representative of two independent experiments.
(D) Impact of adjuvants on eliciting CAR T-vax induced antigen spreading. EGFRvIII^+^CT-2A tumor-bearing C57BL/6 mice received lymphodepletion (LD) and subsequent treatment with CAR-T in the absence or presence of amph-pepvIII vaccine formulated with different adjuvants administered following the same timeline as in Fig. 1C. Shown is IFN-γ ELISPOT monitoring endogenous T-cell priming across various conditions at day 21 as in (C).
(E) Schematic of CAR T-vax therapy using a combination of amph-FITC vaccine and FITC/TA99 bispecific CAR T-cells.
(F) Tumor antigen specificity of endogenous TILs in mice receiving CAR T vs CAR T-vax therapy. C57BL/6 mice bearing OVA-expressing EGFRvIII^+^CT-2A tumors received lymphodepletion and CAR T transfer followed by two weekly vaccinations. Upper panel, experimental timeline. Lower panel, representative flow cytometry plots showing SIINFEKL-tetramer staining of endogenous CD8^+^ T cells within tumors isolated from mice treated with CAR T or CAR T-vax therapy on day 21.
Throughout, *n*=5 animals/group. In Panel C-D, H, data shown are mean ± 95% CI. **, p<0.01; *, p<0.05; ns, not significant by one-way ANOVA with Tukey’s post-test. In panel F, data shown are mean ± 95% CI. *, p<0.05; ns, not significant by Student’s *t*-test.

2 Figure S2. Single cell phenotyping and differential gene expression analysis, related to Figure 2 (A) Dot plot showing differential expression of selected genes in different T cell subtypes as a result of vaccine boosting of CAR T-cells in Fig 2.
(B) Volcano plot showing differential gene expression in intratumoral CD8^+^ CTLs between CAR T-vax vs. CAR-T alone group on day 7 in Fig 2.
(C) Volcano plot showing differential gene expression in intratumoral CD8^+^ CTLs between CAR T-vax and CAR-T alone group on day 14 in Fig 2.
(D) Volcano plot showing differential gene expression in intratumoral CD4^+^ Th cells between CAR T-vax and CAR-T alone group on day 7 in Fig 2.
(E) Volcano plot showing differential gene expression in intratumoral CD4^+^ Th cells between CAR T-vax and CAR-T alone group on day 14 in Fig 2.
(F) UMAP of T cells with detectable TCR alpha, beta or both chains. TCR data was extracted from the scRNA-seq data of Fig 2.
(G) Stacked charts showing proportions of T cells with detectable TCR alpha, beta or both chains on day 7 and day 14, respectively.

3 Figure S3. Characterization of the phenotype and polyfunctionality of intratumoral endogenous T cells and their contributions to long-term tumor control, related to Figure 2 and Figure 3 (A) CD45.2^+^ C57BL/6 mice bearing EGFRvIII^+^CT-2A tumors were treated by CD45.1^+^ EGFRvIII-CAR T ± vax as in Fig 2B. On day 7 post therapy, tumor-infiltrating endogenous CD8^+^ T cells were analyzed for checkpoint marker (PD1, Tim3) expression by flow cytometry. Shown is one representative of two independent experiments.
(B) Cytokine polyfunctionality in PD1^+^TIM3^−^ and PD1^+^TIM3^+^ tumor-infiltrating endogenous CD45.2^+^ CD8^+^ T cells from mice in Fig. S3A. Tumors were dissociated into single cell suspensions on day 7 post therapy and cultured in the presence of 1x cell stimulation cocktail and Golgi plug for 6 hours followed by intracellular cytokine staining and flow cytometry analysis.
(C) Granzyme B expression in PD1^+^TIM3^−^ and PD1^+^TIM3^+^ tumor-infiltrating endogenous CD45.2^+^ CD8^+^ T cells from mice in Fig. S3A and analyzed via intracellular staining as in Fig. S3B.
(D) Histogram showing retroviral Env expression on CT-2A cells, MC38 cells and TC-1 cells. Cells were stained with anti-Env primary antibody followed by anti-IgG isotype secondary antibody. MC38 and TC-1 cells were included as positive and negative controls^67^, respectively.
(E) mouse IL-2 ELISA showing p15E antigen expression in CT-2A cells as detected by T cells. CT-2A cells, MC38 cells and TC-1 cells were pre-treated with IFN-γ and co-cultured with 58 T-cell hybridoma expressing an irrelevant 2C TCR or a p15E-reactive 7PPG2 TCR at 1:1 E:T ratio for 24hr. The supernatant was collected for ELISA.
(F) CDR3 sequences and gene usage for top-ranked p15E-specific T cell clones based on the CDR3 sequences and consensus motifs reported in Grace et al ^68^. The consensus motifs were underlined in CDR3α or CDR3β sequences.
(G) Long-term growth of tumors in WT (*n*=10 animals/group) or Rag1^−/−^ mice (*n*=5 animals/group) in Fig 3H. Only the EGFRvIII^+^ CT-2A and EGFRvIII^−^ CT-2A 100:0 ratio group is shown here.
(H-I) C57BL/6 mice bearing mixed (80% WT + 20% TRP1^−/−^) B16F10 tumors received lymphodepletion (LD) and were left untreated or treated with FTIC/TA99 CAR T-vax therapy as in Fig. 1H. Shown is IFN-γ ELISPOT monitoring endogenous T-cell priming across various conditions following restimulation with irradiated IFN-γ-pretreated Trp1^−/−^ B16F10 cells (H) and tumor growth (I).
In panel A-C, H, *n*=4–5 animals/group, data shown are mean ± 95% CI. *, p<0.05; ns, not significant by Student’s *t*-test. In panel D-E, n=4 biol. replicates/group, data shown are mean ± 95% CI.***, p<0.001; ns, not significant by one-way ANOVA with Tukey’s post-test. In panel J, *n*=5 animals/group, ***, p<0.001; by two-way ANOVA with Tukey’s post-test.

4 Figure S4. Characterization of the phenotype and polyfunctionality of CAR T-cells and the impact of IFN-γ blockade on antigen spreading, related to Figure 4 (A) CD45.2 EGFRvIII^+^CT-2A tumor-bearing mice (n=3–7 animals/group) were treated with CD45.1 EGFRvIII CAR T-cells ± vaccine boosting. Seven days later, splenic CAR T-cells were purified by FACS and processed for quantitative real-time PCR. Gene expression in individual samples from both groups were normalized to the average gene expression in T cells from the CAR T-treated group. Shown are the fold change in the expression of representative genes.
(B) CD45.2^+^ C57BL/6 mice bearing EGFRvIII^+^CT-2A tumors were treated by CD45.1^+^ EGFRvIII-CAR T ± vax as in Fig. 5A. On day 7 post therapy, tumor-infiltrating CAR T-cells were analyzed for checkpoint marker (PD1, Tim3) expression by flow cytometry. Shown is one representative of two independent experiments.
(C-D) Cytokine polyfunctionality in PD1^+^TIM3^−^ (C) and PD1^+^TIM3^+^ (D) tumor-infiltrating CD45.1^+^ CAR T-cells from mice in Fig. S4B. Tumors were dissociated into single cell suspensions on day 7 post therapy and cultured in the presence of 1x cell stimulation cocktail and Golgi plug for 6 hours followed by intracellular cytokine staining and flow cytometry analysis.
(E-F) C57BL/6 mice bearing EGFRvIII^+^CT-2A tumors received lymphodepletion (LD) and were treated with CAR T, or CAR T-vax ± anti-IFNγ. Priming of endogenous CD8 (E) or CD4 (F) T cells in each condition was assessed by IFN-γ ELISPOT as in Fig. 1C.
Throughout, *n*=5 animals/group, error bars are mean ± 95% CI, **p<0.01, *p<0.05, n.s not significant by Student’s t-test for A-D, or by one-way ANOVA with Tukey’s post-test for E-F.

5 Figure S5. Impact of IFN-γ blockade on the functionality and clonality of intratumoral endogenous T cells during CAR T-vax treatment at day 14, related to Figure 2 (A) UMAP of endogenous T cells obtained from the day 14 tumors in CAR-T- and CAR T-vax-treated mice in Fig. 2 as well as a group of mice receiving CAR T-vax + anti-IFNγ treatment. T cells were randomly down-sampled to show an even number of points from each treatment condition. T cells are colored by the treatment group.
(B) Curated clusters based on signature gene expression of day 14 T cells in Fig. S5A. Th, T helper cells. Treg, regulatory T cell. CTL, cytotoxic lymphocyte.
(C) Stacked charts showing proportions of different clusters within day 14 T cells under each treatment condition in Fig. S5A.
(D) Volcano plot showing differential gene expression in day 14 CD8^+^ CTLs between CAR T-vax and CAR T-vax + anti IFNγ group in Fig. S5A.
(E) Volcano plot showing differential gene expression in day 14 CD4^+^ Th cells between CAR T-vax and CAR T-vax + anti IFNγ group in Fig.S5A.
(F) Cytotoxicity score of endogenous retroviral antigen p15E-specific TILs and TILs of unknown specificity on day 14 from CAR T, CAR T-vax and CAR T-vax + IFNγ groups.
Data shown are mean ± 95% CI. ****, p<0.0001; *, p<0.05 by two-sided Wilcoxon rank-sum test. See methods for the definition and calculation of the cytotoxicity score.

6 Figure S6. Characterization of intratumoral CAR T, tumor cell, and DC responses to therapy, related to Figure 6 (A) Flow cytometry analysis showing intracellular granzyme B expression in CAR T-cells from mice in Fig. 6B.
(B) Tumors isolated from each group in Fig. 6B were dissociated into single cell suspensions for flow cytometry analysis of surface calreticulin expression on tumor cells in response to CAR T vs. CAR T-vax therapy.
(C-E) Intratumoral macrophage phenotypes are shifted by CAR T-vax therapy. C57BL/6 mice bearing EGFRvIII^+^CT-2A tumors (*n* = 5 animals/group) were treated by WT CAR T ± vax as in Fig 6B, and intratumoral macrophages were analyzed for CD40 (C), CD80/86 (D) levels as well as the percentage of suppressive M2 (CD11b^+^F4/80^+^CD206^+^) macrophages (E) by flow cytometry on day 14 as shown in the timeline. See methods for surface markers used to define M2 macrophages.
(F) Representative histogram showing surface CCR7 expression on intratumoral CD103^+^ DCs from mice in Fig. 6F.
(G) Representative histogram showing surface CCR7 expression on intratumoral CD11b^+^ DCs from mice in Fig. 6F.
(H-I) Flow cytometry analysis showing CD80/CD86 expression in intratumoral CD103^+^ DCs (H) or CD11b^+^ DCs (I) from mice in Fig. 6G–H.
(J-K) Flow cytometry analysis showing MHCII expression in intratumoral CD103^+^ DCs (J) or CD11b^+^ DCs (K) from mice in Fig. 6G–H.
(L-N) CAR T-vax therapy using WT or IFN-γ KO CAR T cells does not lead to substantial changes in the tumor vasculature. Total vessel diameter (L) and vessel volume (M) was calculated using the Filament tracing algorithm on the masked anti-CD31 channel. Hoechst area were calculated using the Analyze particles function in Fiji^69^ as % of tumor area perfused (N).
Throughout, *n*=5 animals/group. Shown are representative flow histograms and geom. mean fluorescence intensities. Data shown are mean ± 95% CI. ****, p<0.0001; ***, p<0.001, **, p<0.01, *, p<0.05 by Student’s *t*-test for A-E, and by one-way ANOVA with Tukey’s post-test for H-N.

7 Figure S7. Impact of CAR T-intrinsic IFN-γ signaling on CAR T polyfunctionality, subsequent tumor killing, stimulation of DC antigen uptake and systemic toxicity in response to vaccine boosting, related to Figure 2 (A-H) CD45.1^+^ C57BL/6 mice bearing EGFRvIII^+^CT-2A tumors received lymphodepletion (LD) and were treated with CD45.2^+^ WT or IFNGR1^−/−^CAR T-vax as shown in the timeline (A). Tumors isolated from each group at day 7 post CAR T-vax therapy were dissociated into single cell suspensions for flow cytometry analysis.
(B) IFN-γ expression in CD45.2^+^ CAR T-cells from WT or IFNGR1^−/−^ CAR T-vax treated group.
(C)TNF-α expression in CD45.2^+^ CAR T-cells from WT or IFNGR1^−/−^ CAR T-vax treated group.
(D) Granzyme B expression in CD45.2^+^ CAR T-cells from WT or IFNGR1^−/−^ CAR T-vax treated group.
(D) Flow cytometry analysis showing surface expression of calreticulin on tumor cells.
(E) Flow cytometry analysis showing tumor antigen uptake by intratumoral CD45.2^+^ CD103^+^DCs. ZsGreen was used as a surrogate antigen in this experiment.
(F) Flow cytometry analysis showing tumor antigen uptake by intratumoral CD45.2^+^ CD11b^+^ DCs. ZsGreen was used as a surrogate antigen in this experiment.
(G) Flow cytometry analysis showing tumor antigen uptake by LN-resident CD45.2^+^ CD8^+^ DCs. ZsGreen was used as a surrogate antigen in this experiment.
(H) Flow cytometry analysis showing tumor antigen uptake by LN-resident CD45.2^+^ CD11b^+^ DCs. ZsGreen was used as a surrogate antigen in this experiment.
(J-K) C57BL/6 mice bearing mixed CT-2A tumor (80% EGFRvIII^+^ CT-2A + 20% WT CT2A cells) were treated as in Fig. 1C with vaccine only, CAR T or NFAT-IFNγ CAR T in the presence or absence of vaccination. Shown are IFN-γ levels in serum (J) from day 6 (before 1^st^ vax), day 8 (24hr post 1^st^ vax) and day 14 (day 6 post 1^st^ vax) and day 15 (24hr post 2^nd^ vax) and animal body weight change (K).
Throughout, a representative histogram from each treatment group and the summary data are shown. *n*=5 animals/group, data shown are mean ± 95% CI. **, p<0.01; *, p<0.05; ns, not significant by Student’s *t*-test for B-I, and by two-way ANOVA with Turkey’s multiple comparisons test for J.

8 Supplemental Table 1
Differential gene expression for the single cell clusters, related to Figure 2.

9 Supplemental Table 2
List of genes differentially expressed in CD8^+^ and CD4^+^ TILs at day 7 and day 14 between CAR T and CAR T-vax treatment groups, related to Figure 2.

10 Supplemental Table 3
List of T cell clones with predicted p15E-antigen specificity in T cells, related to Figure 2.

11 Supplemental Table 4
List of genes differentially expressed in splenic CAR T-cells treated with or without vaccination, related to Figure 4.

12 Supplemental Table 5
List of genes differentially expressed in CD8^+^ and CD4^+^ TILs at day 14 from mice receiving CAR T-vax with or without IFNγ blockade, related to Figure 2.

## Figures and Tables

**Table:** KEY RESOURCES TABLE

REAGENT or RESOURCE	SOURCE	IDENTIFIER
**Antibodies**		
Anti-mouse CD3 (17A2) Alex488	BioLegend	100220; RRID:AB_1732057
Anti-mouse CD8a (53–6.7) BUV395	BD Biosciences	563786; RRID:AB_2732919
Anti-mouse CD8a (53–6.7) BV421	BioLegend	100738; RRID:AB_11204079
Anti-mouse CD4 (RM4–5) FITC	BioLegend	100510; RRID:AB_312713
Anti-mouse CD25 (PC61) APC-Cy7	BioLegend	102026; RRID:AB_830745
Anti-mouse B220 (RA3–6B2) PE-cy7	BioLegend	103222; RRID: AB_313005
Anti-mouse PD-1 (29F.1A12) BV421	BioLegend	135218; RRID:AB_2561447
Anti-mouse TIM3 (RMT3–23) APC	BioLegend	119706; RRID:AB_2561656
Anti-mouse CD45 (30-F11) Percp-cy5.5	BioLegend	103132 RRID: AB_893340
Anti-mouse CD45.1 (A20) BV421	BioLegend	110732 BRID: AB_2562563
Anti-mouse CD45.2 (104) BUV737	BD Biosciences	612778; RRID:AB_2870107
Anti-mouse CD317 (927) Alex488	BioLegend	127012 RRID: AB_1953287
Anti-mouse CD11c (N418) FITC	BioLegend	117306; RRID:AB_313775
Anti-mouse CD11b (M1/70) APC-Cy7	BioLegend	101226; RRID:AB_830642
Anti-mouse CD24 (M1/69) BV711	BioLegend	563450; RRID:AB_2738213
Anti-mouse MHC II (M5/114.15.2) BV605	BioLegend	107639; RRID:AB_2565894
Anti-mouse F4/80 (T45–2342) BUV395	BD Biosciences	565614; RRID:AB_2739304
Anti-mouse CD86 (GL-1) PE-Dazzle 594	BioLegend	105042; RRID:AB_2566409
Anti-mouse CD103 (2E7) PE	BioLegend	121406; RRID:AB_1133989
Anti-mouse CD45.1 (A20) APC	BioLegend	110714; RRID:AB_313503
Anti-mouse IFN-γ (XMG1.2) PE	BioLegend	505808; RRID:AB_315402
Anti-mouse TNF-α (MP6-XT22) APC	BioLegend	506308; AB_315429
Anti-mouse Granzyme B (QA16A02) APC	BioLegend	372204; RRID:AB_2687028
Anti-mouse FoxP3 (150D) PE	BioLegend	320007 AB_492981
Anti-mouse PGC-1a (D-5) PE	Santa Cruz	sc-518025 PE
Anti-mouse Ki67 (11F6) BV421	BioLegend	151208 RRID: AB_2629748
Anti-mouse CD206 PE	BioLegend	141706 RRID: AB_10895754
1E4.2.1 anti-Env antibody	Wittrup lab at MIT	N/A
Anti-mouse IFN-γ (XMG1.2)	BioXCell	BE0055; RRID:AB_1107694
Anti-mouse TNF-α (XT3.11)	BioXCell	BE0058; RRID:AB_1107764
Anti-mouse CD3ε (2C11)	BioXCell	BE0001–1 BRID:AB_1107634
Anti-mouse CD28 (37.51)	BioXCell	BE0015–1 BRID:AB_1107624
Anti-CD45.1 (A20)	Stem cell Tech	60117BT
Anti-CD45.2 (104)	Stem cell Tech	60118BT
**Bacterial and virus strains**		
5-alpha Competent *E. coli*	New England Biolabs	C2987U
**Chemicals, peptides and recombinant proteins**		
1,2-distearoyl-sn-glycero-3-phosphoethanolamine-N-[maleimide (polyethylene glycol)-2000]	Layson Bio	100220
DSPE-PEG-FITC	Avanti	810120
Cyclic-di-GMP	invivogen	tlrl-nacdg
Resiquimod	invivogen	tlrl-r848
GolgiPlug^™^ Protein Transport Inhibitor (containing Brefeldin A)	BD Biosciences	BDB555029
Cell Stimulation Cocktail	eBioscience	00–4970-93
Protease Inhibitor Cocktail	Roche	5892970001
Recombinant murine IL-2	Biolegend	575408
Recombinant murine IFN-γ	Peprotech	315–05
DNase I	Sigma Aldrich	10104159001
Collagenase IV	Worthington	LS004188
CalPhos^™^ Mammalian Transfection Kit	Takara	631312
Sytox Red	Thermo Fisher	S34859
Retronectin	Takara	T100B
TRIzol^™^ Reagent	Thermo Fisher	15596018
iTAg Tetramer/PE – H-2 Kb OVA (SIINFEKL)	MBL international	TB-5001–1
**Critical commercial assays**		
NucleoSpin^®^ Plasmid	Takara	740588.250
TrypLE^™^ Express Enzyme	Thermo Fisher	12605036
Gibco ACK Lysing Buffer	Thermo Fisher	A10492–01
CellTrace Violet	Thermo Fisher	C34557
LIVE/DEAD^™^ Fixable Aqua Dead Cell Stain Kit, for 405 nm excitation	Thermo Fisher	L34966
FITC Annexin V Apoptosis Detection Kit	BD Biosciences	556547
Fixation/Permeabilization Solution Kit	BD Biosciences	554714
Foxp3 / Transcription Factor Staining Buffer Set	eBioscience	00–5523-00
Mouse CD45 microbeads	Miltenyi Biotec	130–052-301
EasySep^™^ Mouse CD8+ T Cell Isolation Kit	Stemcell Technologies	19853
Mouse IFN-γ ELISA kit	R&D systems	DY485
Mouse IL-2 ELISA kit	Invitrogen	88–7024
Mouse IFN-γ ELISPOT Kit	BD Biosciences	551083
RNeasy Micro Kit	Qiagen	74004
T-PERTM	Thermo Fisher	78510
Proteinase and phosphatase inhibitors	Thermo Fisher	78442
iScript^™^ Reverse Transcription Supermix	Biorad	1708841
LEGENDplex^™^ assays	BioLegend	740621
**Deposited data**		
Bulk RNA-seq data	GEO	GSE211938
sc RNA-seq data	GEO	GSE212453
**Experimental Models: Cell Lines**		
B16F10 cells	ATCC	CRL-6475; RRID:CVCL_0159
CT-2A cells	T. Seyfried Lab at Boston college	N/A
MHCII^+^ CT-2A cells	Generated in the Irvine lab	N/A
mEGFRvIII-CT-2A cells	Generated in the Irvine lab	N/A
ZsGreen^+^ mEGFRvIII-CT-2A cells	Generated in the Irvine lab	N/A
mEGFRvIII-CT-2A-OVA cells	Generated in the Irvine lab	N/A
293 phoenix cells	ATCC	CRL-3214
B16F10-OVA cells	G. Dranoff Lab at DFCI	N/A
2C TCR-58^−/−^ T cell hybridoma cells	Birnbaum lab at MIT	N/A
7PPG2 TCR-58^−/−^ T cell hybridoma cells	Birnbaum lab at MIT	N/A
MC38 cells	Wittrup lab at MIT	N/A
TC-1 cells	ATCC	CRL-2493
TRP1^−/−^ B16F10 cells	Generated in the Irvine lab	N/A
**Experimental Models: Organism/Strains**		
C57BL/6J mice, CD45.2^+^	Jackson Laboratory	000624; RRID:IMSR_JAX:000624
C57BL/6J mice, CD45.1^+^	Jackson Laboratory	002014; RRID:IMSR_JAX:002014
Rag1^−/−^ (B6.129S7-*Rag1*^*tm1Mom*^/J)	Jackson Laboratory	013755; RRID:IMSR_JAX:013755
IFNGR1^−/−^ (B6.129S7-*Ifngr1*^*tm1Agt*^/J)	Jackson Laboratory	003288; RRID:IMSR_JAX:00328
Batf3^−/−^ (B6.129S(C)-*Batf3*^*tm1Kmm*^/J)	Jackson Laboratory	013755; RRID:IMSR_JAX:013755
CD11c-cre (C57BL/6J-Tg(Itgax-cre,-EGFP)^4097Ach^/J )	Jackson Laboratory	007567; RRID:IMSR_JAX:007567
IFNGR1-flox (C57BL/6N-*Ifngr1*^*tm1.1Rds*^/J)	Jackson Laboratory	025394; RRID:IMSR_JAX:025394
PGC-1α-flox (B6N.129(FVB)-*Ppargc1a*^*tm2.1Brsp*^/J)	Jackson Laboratory	009666 RRID:IMSR_JAX:009666
LCK-cre (B6.Cg-Tg(Lck-cre)^548Jxm^/J)	Jackson Laboratory	003802 RRID:IMSR_JAX:003802
IL12rb2^−/−^ (B6;129S1-*Il12rb2*^*tm1Jm*^/J)	Jackson Laboratory	003248 RRID:IMSR_JAX:003248
IL12p40^−/−^ (B6.129S1-*Il12b*^*tm1Jm*^/J	Jackson Laboratory	002693 RRID:IMSR_JAX:002693
IFNγ ^−/−^ (B6.129S7-Ifng^tm1Ts^/J)	Jackson Laboratory	002287; RRID:IMSR_JAX:002287
CD8α ^−/−^ (B6.129S2-Cd8^atm1Mak^/J)	Jackson Laboratory	002665 RRID:IMSR_JAX:002665
**Oligonucleotides**		
Ccl4 qPCR primers For: CCAAGCCAGCTGTGGTATTCC Rev: GAGCTGCTCAGTTCAACTCC	Sigma-Aldrich	N/A
Ccl5 qPCR primers For: GCTGCTTTGCCTACCTCTCC Rev: TCGAGTGACAAACACGACTGC	Sigma-Aldrich	N/A
Itgb1 qPCR primers For: ATGCCAAATCTTGCGGAGAAT Rev: TTTGCTGCGATTGGTGACATT	Sigma-Aldrich	N/A
Itga4 qPCR primers For: GATGCTGTTGTTGTACTTCGGG Rev: ACCACTGAGGCATTAGAGAGC	Sigma-Aldrich	N/A
Cx3cr1 qPCR primers For: CCCATCTGCTCAGGACCTC Rev: ATGGTTCCAAAGGCCACAATG	Sigma-Aldrich	N/A

## Highlights

Vaccine boosting enhances CAR T cell metabolism and polyfunctionality

Vaccine-boosted CAR T therapy elicits robust and potent antigen spreading

Antigen spreading supports CAR T therapy to treat antigenically heterogeneous tumors

CAR T-derived IFNγ and DC-derived IL12 are critical for sustaining antigen spreading

## References

[1] Irvine DJ, Maus MV, Mooney DJ, and Wong WW (2022). The future of engineered immune cell therapies. Science 378, 853–858. 10.1126/science.abq6990. 36423279 PMC9919886

[2] Labanieh L, and Mackall CL (2022). CAR immune cells: design principles, resistance and the next generation. Nature 614, 635–648. 10.1038/s41586-023-05707-3. 36813894

[3] Wang M, Munoz J, Goy A, Locke FL, Jacobson CA, Hill BT, Timmerman JM, Holmes H, Jaglowski S, Flinn IW, (2020). KTE-X19 CAR T-Cell Therapy in Relapsed or Refractory Mantle-Cell Lymphoma. New Engl J Med 382, 1331–1342. 10.1056/nejmoa1914347. 32242358 PMC7731441

[4] Maude SL, Laetsch TW, Buechner J, Rives S, Boyer M, Bittencourt H, Bader P, Verneris MR, Stefanski HE, Myers GD, (2018). Tisagenlecleucel in Children and Young Adults with B-Cell Lymphoblastic Leukemia. New Engl J Medicine 378, 439–448. 10.1056/nejmoa1709866. PMC5996391 29385370

[5] Abramson JS, Palomba ML, Gordon LI, Lunning MA, Wang M, Arnason J, Mehta A, Purev E, Maloney DG, Andreadis C, (2020). Lisocabtagene maraleucel for patients with relapsed or refractory large B-cell lymphomas (TRANSCEND NHL 001): a multicentre seamless design study. Lancet 396, 839–852. 10.1016/s0140-6736(20)31366-0. 32888407

[6] Rafiq S, Hackett CS, and Brentjens RJ (2020). Engineering strategies to overcome the current roadblocks in CAR T cell therapy. Nat Rev Clin Oncol 17, 147–167. 10.1038/s41571-019-0297-y. 31848460 PMC7223338

[7] Roselli E, Faramand R, and Davila ML (2021). Insight into next-generation CAR therapeutics: designing CAR T cells to improve clinical outcomes. J Clin Invest 131, e142030. 10.1172/jci142030. 33463538 PMC7810492

[8] Hou AJ, Chen LC, and Chen YY (2021). Navigating CAR-T cells through the solid-tumour microenvironment. Nat Rev Drug Discov 20, 531–550. 10.1038/s41573-021-00189-2. 33972771

[9] O’Rourke DM, Nasrallah MP, Desai A, Melenhorst JJ, Mansfield K, Morrissette JJD, Martinez-Lage M, Brem S, Maloney E, Shen A, (2017). A single dose of peripherally infused EGFRvIII-directed CAR T cells mediates antigen loss and induces adaptive resistance in patients with recurrent glioblastoma. Sci Transl Med 9, eaaa0984. 10.1126/scitranslmed.aaa0984. 28724573 PMC5762203

[10] Shah NN, and Fry TJ (2019). Mechanisms of resistance to CAR T cell therapy. Nature Reviews Clinical Oncology, 1–14. 10.1038/s41571-019-0184-6. PMC8214555 30837712

[11] Landsberg J, Kohlmeyer J, Renn M, Bald T, Rogava M, Cron M, Fatho M, Lennerz V, Wölfel T, Hölzel M, (2012). Melanomas resist T-cell therapy through inflammation-induced reversible dedifferentiation. Nature 490, 412–416. 10.1038/nature11538. 23051752

[12] Gulley JL, Madan RA, Pachynski R, Mulders P, Sheikh NA, Trager J, and Drake CG (2017). Role of Antigen Spread and Distinctive Characteristics of Immunotherapy in Cancer Treatment. Jnci J National Cancer Inst 109, djw261. 10.1093/jnci/djw261. PMC5441294 28376158

[13] Kvistborg P, Philips D, Kelderman S, Hageman L, Ottensmeier C, Joseph-Pietras D, Welters MJP, Burg S van der, Kapiteijn, E., Michielin, O., (2014). Anti–CTLA-4 therapy broadens the melanoma-reactive CD8+ T cell response. Sci Transl Med 6, 254ra128. 10.1126/scitranslmed.3008918. 25232180

[14] Brossart P (2020). The role of antigen-spreading in the efficacy of immunotherapies. Clin Cancer Res, clincanres.0305.2020. 10.1158/1078-0432.ccr-20-0305. 32357962

[15] Awad MM, Govindan R, Balogh KN, Spigel DR, Garon EB, Bushway ME, Poran A, Sheen JH, Kohler V, Esaulova E, (2022). Personalized neoantigen vaccine NEO-PV-01 with chemotherapy and anti-PD-1 as first-line treatment for non-squamous non-small cell lung cancer. Cancer Cell 40, 1010–1026.e11. 10.1016/j.ccell.2022.08.003. 36027916

[16] Beatty GL, Haas AR, Maus MV, Torigian DA, Soulen MC, Plesa G, Chew A, Zhao Y, Levine BL, Albelda SM, (2014). Mesothelin-Specific Chimeric Antigen Receptor mRNA-Engineered T Cells Induce Antitumor Activity in Solid Malignancies. Cancer Immunol Res 2, 112–120. 10.1158/2326-6066.cir-13-0170. 24579088 PMC3932715

[17] Kim RH, Plesa G, Gladney W, Kulikovskaya I, Levine BL, Lacey SF, June CH, Melenhorst JJ, and Beatty GL (2017). Effect of chimeric antigen receptor (CAR) T cells on clonal expansion of endogenous non-CAR T cells in patients (pts) with advanced solid cancer. J Clin Oncol 35, 3011–3011. 10.1200/jco.2017.35.15_suppl.3011.

[18] Hegde M, Joseph SK, Pashankar F, DeRenzo C, Sanber K, Navai S, Byrd TT, Hicks J, Xu ML, Gerken C, (2020). Tumor response and endogenous immune reactivity after administration of HER2 CAR T cells in a child with metastatic rhabdomyosarcoma. Nat Commun 11, 3549. 10.1038/s41467-020-17175-8. 32669548 PMC7363864

[19] Hegde M, Joseph SK, Pashankar F, DeRenzo C, Sanber K, Navai S, Byrd TT, Hicks J, Xu ML, Gerken C, (2020). Tumor response and endogenous immune reactivity after administration of HER2 CAR T cells in a child with metastatic rhabdomyosarcoma. Nat Commun 11, 3549. 10.1038/s41467-020-17175-8. 32669548 PMC7363864

[20] Alizadeh D, Wong RA, Gholamin S, Maker M, Aftabizadeh M, Yang X, Pecoraro JR, Jeppson JD, Wang D, Aguilar B, (2021). IFNγ is Critical for CAR T Cell–mediated Myeloid Activation and Induction of Endogenous Immunity. Cancer Discov 11, 2248–2265. 10.1158/2159-8290.cd-20-1661. 33837065 PMC8561746

[21] Klampatsa A, Leibowitz MS, Sun J, Liousia M, Arguiri E, and Albelda SM (2020). Analysis and Augmentation of the Immunologic Bystander Effects of CAR T Cell Therapy in a Syngeneic Mouse Cancer Model. Mol Ther Oncolytics 18, 360–371. 10.1016/j.omto.2020.07.005. 32802940 PMC7417672

[22] Lai J, Mardiana S, House IG, Sek K, Henderson MA, Giuffrida L, Chen AXY, Todd KL, Petley EV, Chan JD, (2020). Adoptive cellular therapy with T cells expressing the dendritic cell growth factor Flt3L drives epitope spreading and antitumor immunity. Nat Immunol, 1–13. 10.1038/s41590-020-0676-7. 32424363

[23] Kuhn NF, Lopez AV, Li X, Cai W, Daniyan AF, and Brentjens RJ (2020). CD103+ cDC1 and endogenous CD8+ T cells are necessary for improved CD40L-overexpressing CAR T cell antitumor function. Nat Commun 11, 6171. 10.1038/s41467-020-19833-3. 33268774 PMC7710757

[24] Kueberuwa G, Kalaitsidou M, Cheadle E, Hawkins RE, and Gilham DE (2018). CD19 CAR T Cells Expressing IL-12 Eradicate Lymphoma in Fully Lymphoreplete Mice through Induction of Host Immunity. Molecular Therapy: Oncolytics 8, 41–51. 10.1016/j.omto.2017.12.003. 29367945 PMC5772011

[25] Etxeberria I, Bolaños E, Quetglas JI, Gros A, Villanueva A, Palomero J, Sánchez-Paulete AR, Piulats JM, Matias-Guiu X, Olivera I, (2019). Intratumor Adoptive Transfer of IL-12 mRNA Transiently Engineered Antitumor CD8+ T Cells. Cancer Cell 36, 613–629.e7. 10.1016/j.ccell.2019.10.006. 31761658

[26] Chmielewski M, and Abken H (2017). CAR T Cells Releasing IL-18 Convert to T-Bethigh FoxO1low Effectors that Exhibit Augmented Activity against Advanced Solid Tumors. Cell Reports 21, 3205–3219. 10.1016/j.celrep.2017.11.063. 29241547

[27] Adachi K, Kano Y, Nagai T, Okuyama N, Sakoda Y, and Tamada K (2018). IL-7 and CCL19 expression in CAR-T cells improves immune cell infiltration and CAR-T cell survival in the tumor. Nature Biotechnology 36, 346–351. 10.1038/nbt.4086. 29505028

[28] Park AK, Fong Y, Kim S-I, Yang J, Murad JP, Lu J, Jeang B, Chang W-C, Chen NG, Thomas SH, (2020). Effective combination immunotherapy using oncolytic viruses to deliver CAR targets to solid tumors. Sci Transl Med 12. 10.1126/scitranslmed.aaz1863. PMC9126033 32878978

[29] Walsh SR, Simovic B, Chen L, Bastin D, Nguyen A, Stephenson K, Mandur TS, Bramson JL, Lichty BD, and Wan Y (2019). Endogenous T cells prevent tumor immune escape following adoptive T cell therapy. J Clin Invest 129, 5400–5410. 10.1172/jci126199. 31682239 PMC6877330

[30] Zhang L, Morgan RA, Beane JD, Zheng Z, Dudley ME, Kassim SH, Nahvi AV, Ngo LT, Sherry RM, Phan GQ, (2015). Tumor-Infiltrating Lymphocytes Genetically Engineered with an Inducible Gene Encoding Interleukin-12 for the Immunotherapy of Metastatic Melanoma. Clin Cancer Res 21, 2278–2288. 10.1158/1078-0432.ccr-14-2085. 25695689 PMC4433819

[31] Kerkar SP, Muranski P, Kaiser A, Boni A, Sanchez-Perez L, Yu Z, Palmer DC, Reger RN, Borman ZA, Zhang L, (2010). Tumor-Specific CD8+ T Cells Expressing Interleukin-12 Eradicate Established Cancers in Lymphodepleted Hosts. Cancer Res 70, 6725–6734. 10.1158/0008-5472.can-10-0735. 20647327 PMC2935308

[32] Ma L, Dichwalkar T, Chang JYH, Cossette B, Garafola D, Zhang AQ, Fichter M, Wang C, Liang S, Silva M, (2019). Enhanced CAR–T cell activity against solid tumors by vaccine boosting through the chimeric receptor. Science 365, 162–168. 10.1126/science.aav8692. 31296767 PMC6800571

[33] Liu H, Moynihan KD, Zheng Y, Szeto GL, Li AV, Huang B, Egeren DSV, Park C, and Irvine DJ (2014). Structure-based programming of lymph-node targeting in molecular vaccines. Nature 507, 519–522. 10.1038/nature12978. 24531764 PMC4069155

[34] Szabo PA, Levitin HM, Miron M, Snyder ME, Senda T, Yuan J, Cheng YL, Bush EC, Dogra P, Thapa P, (2019). Single-cell transcriptomics of human T cells reveals tissue and activation signatures in health and disease. Nat Commun 10, 4706. 10.1038/s41467-019-12464-3. 31624246 PMC6797728

[35] Tibbitt CA, Stark JM, Martens L, Ma J, Mold JE, Deswarte K, Oliynyk G, Feng X, Lambrecht BN, Bleser PD, (2019). Single-Cell RNA Sequencing of the T Helper Cell Response to House Dust Mites Defines a Distinct Gene Expression Signature in Airway Th2 Cells. Immunity 51, 169–184.e5. 10.1016/j.immuni.2019.05.014. 31231035

[36] LeBleu VS, O’Connell JT, Herrera KNG, Wikman H, Pantel K, Haigis MC, Carvalho F.M. de, Damascena A, Chinen LTD, Rocha RM, (2014). PGC-1α mediates mitochondrial biogenesis and oxidative phosphorylation in cancer cells to promote metastasis. Nat Cell Biol 16, 992–1003. 10.1038/ncb3039. 25241037 PMC4369153

[37] Fernandez-Marcos PJ, and Auwerx J (2011). Regulation of PGC-1α, a nodal regulator of mitochondrial biogenesis. Am J Clin Nutrition 93, 884S–890S. 10.3945/ajcn.110.001917. 21289221 PMC3057551

[38] Vardhana SA, Hwee MA, Berisa M, Wells DK, Yost KE, King B, Smith M, Herrera PS, Chang HY, Satpathy AT, (2020). Impaired mitochondrial oxidative phosphorylation limits the self-renewal of T cells exposed to persistent antigen. Nat Immunol 21, 1022–1033. 10.1038/s41590-020-0725-2. 32661364 PMC7442749

[39] Bhat P, Leggatt G, Waterhouse N, and Frazer IH (2017). Interferon-γ derived from cytotoxic lymphocytes directly enhances their motility and cytotoxicity. Cell Death Dis 8, e2836–e2836. 10.1038/cddis.2017.67. 28569770 PMC5520949

[40] Böttcher JP, and Sousa CR e (2018). The Role of Type 1 Conventional Dendritic Cells in Cancer Immunity. Trends Cancer 4, 784–792. 10.1016/j.trecan.2018.09.001. 30352680 PMC6207145

[41] Murphy TL, and Murphy KM (2022). Dendritic cells in cancer immunology. Cell Mol Immunol 19, 3–13. 10.1038/s41423-021-00741-5. 34480145 PMC8752832

[42] Böttcher JP, Bonavita E, Chakravarty P, Blees H, Cabeza-Cabrerizo M, Sammicheli S, Rogers NC, Sahai E, Zelenay S, and Sousa CR e (2018). NK Cells Stimulate Recruitment of cDC1 into the Tumor Microenvironment Promoting Cancer Immune Control. Cell 172, 1022–1037.e14. 10.1016/j.cell.2018.01.004. 29429633 PMC5847168

[43] Vilgelm AE, and Richmond A (2019). Chemokines Modulate Immune Surveillance in Tumorigenesis, Metastasis, and Response to Immunotherapy. Front Immunol 10, 333. 10.3389/fimmu.2019.00333. 30873179 PMC6400988

[44] Garris CS, Arlauckas SP, Kohler RH, Trefny MP, Garren S, Piot C, Engblom C, Pfirschke C, Siwicki M, Gungabeesoon J, (2018). Successful Anti-PD-1 Cancer Immunotherapy Requires T Cell-Dendritic Cell Crosstalk Involving the Cytokines IFN-γ and IL-12. Immunity 49, 1148–1161.e7. 10.1016/j.immuni.2018.09.024. 30552023 PMC6301092

[45] Zhang L, Kerkar SP, Yu Z, Zheng Z, Yang S, Restifo NP, Rosenberg SA, and Morgan RA (2011). Improving Adoptive T Cell Therapy by Targeting and Controlling IL-12 Expression to the Tumor Environment. Mol Ther 19, 751–759. 10.1038/mt.2010.313. 21285960 PMC3070103

[46] Majzner RG, and Mackall CL (2018). Tumor Antigen Escape from CAR T-cell Therapy. Cancer Discov 8, 1219–1226. 10.1158/2159-8290.cd-18-0442. 30135176

[47] Guedan S, Calderon H Jr., A.D.P., and Maus MV (2019). Engineering and Design of Chimeric Antigen Receptors. Molecular Therapy - Methods & Clinical Development 12, 145–156. 10.1016/j.omtm.2018.12.009. 30666307 PMC6330382

[48] Lim WA, and June CH (2017). The Principles of Engineering Immune Cells to Treat Cancer. Cell 168, 724–740. 10.1016/j.cell.2017.01.016. 28187291 PMC5553442

[49] Schroder K, Hertzog PJ, Ravasi T, and Hume DA (2004). Interferon-γ: an overview of signals, mechanisms and functions. J Leukocyte Biol 75, 163–189. 10.1189/jlb.0603252. 14525967

[50] Castro F, Cardoso AP, Gonçalves RM, Serre K, and Oliveira MJ (2018). Interferon-Gamma at the Crossroads of Tumor Immune Surveillance or Evasion. Front Immunol 9, 847. 10.3389/fimmu.2018.00847. 29780381 PMC5945880

[51] Overacre-Delgoffe AE, Chikina M, Dadey RE, Yano H, Brunazzi EA, Shayan G, Horne W, Moskovitz JM, Kolls JK, Sander C, (2017). Interferon-γ Drives Treg Fragility to Promote Anti-tumor Immunity. Cell 169, 1130–1141.e11. 10.1016/j.cell.2017.05.005. 28552348 PMC5509332

[52] Larson RC, Kann MC, Bailey SR, Haradhvala NJ, Llopis PM, Bouffard AA, Scarfó I, Leick MB, Grauwet K, Berger TR, (2022). CAR T cell killing requires the IFNγR pathway in solid but not liquid tumours. Nature 604, 563–570. 10.1038/s41586-022-04585-5. 35418687

[53] Boulch M, Cazaux M, Loe-Mie Y, Thibaut R, Corre B, Lemaître F, Grandjean CL, Garcia Z, and Bousso P (2021). A cross-talk between CAR T cell subsets and the tumor microenvironment is essential for sustained cytotoxic activity. Sci Immunol 6. 10.1126/sciimmunol.abd4344. 33771887

[54] Xu T, Keller A, and Martinez GJ (2019). NFAT1 and NFAT2 Differentially Regulate CTL Differentiation Upon Acute Viral Infection. Front Immunol 10, 184. 10.3389/fimmu.2019.00184. 30828328 PMC6384247

[55] Samten B, Townsend JC, Weis SE, Bhoumik A, Klucar P, Shams H, and Barnes PF (2008). CREB, ATF, and AP-1 Transcription Factors Regulate IFN-γ Secretion by Human T Cells in Response to Mycobacterial Antigen. J Immunol 181, 2056–2064. 10.4049/jimmunol.181.3.2056. 18641343 PMC2587306

[56] Chang C-H, Curtis JD, Maggi LB, Faubert B, Villarino AV, O’Sullivan D, Huang SC-C, van der Windt GJW, Blagih J, Qiu J, (2013). Posttranscriptional Control of T Cell Effector Function by Aerobic Glycolysis. Cell 153, 1239–1251. 10.1016/j.cell.2013.05.016. 23746840 PMC3804311

[57] Savan R (2014). Post-Transcriptional Regulation of Interferons and Their Signaling Pathways. J Interf Cytokine Res 34, 318–329. 10.1089/jir.2013.0117. PMC4015472 24702117

[58] Keating SE, Zaiatz-Bittencourt V, Loftus RM, Keane C, Brennan K, Finlay DK, and Gardiner CM (2016). Metabolic Reprogramming Supports IFN-γ Production by CD56bright NK Cells. J Immunol 196, 2552–2560. 10.4049/jimmunol.1501783. 26873994

[59] Donnelly RP, Loftus RM, Keating SE, Liou KT, Biron CA, Gardiner CM, and Finlay DK (2014). mTORC1-Dependent Metabolic Reprogramming Is a Prerequisite for NK Cell Effector Function. J Immunol 193, 4477–4484. 10.4049/jimmunol.1401558. 25261477 PMC4201970

[60] Gerbec ZJ, Hashemi E, Nanbakhsh A, Holzhauer S, Yang C, Mei A, Tsaih S-W, Lemke A, Flister MJ, Riese MJ, (2020). Conditional Deletion of PGC-1α Results in Energetic and Functional Defects in NK Cells. Iscience 23, 101454. 10.1016/j.isci.2020.101454. 32858341 PMC7474003

[61] Lisci M, Barton PR, Randzavola LO, Ma CY, Marchingo JM, Cantrell DA, Paupe V, Prudent J, Stinchcombe JC, and Griffiths GM (2021). Mitochondrial translation is required for sustained killing by cytotoxic T cells. Science 374, eabe9977. 10.1126/science.abe9977. 34648346

[62] Windt GJW, and Pearce EL (2012). Metabolic switching and fuel choice during T-cell differentiation and memory development. Immunol Rev 249, 27–42. 10.1111/j.1600-065x.2012.01150.x. 22889213 PMC3645891

[63] Scharping NE, Menk AV, Moreci RS, Whetstone RD, Dadey RE, Watkins SC, Ferris RL, and Delgoffe GM (2016). The Tumor Microenvironment Represses T Cell Mitochondrial Biogenesis to Drive Intratumoral T Cell Metabolic Insufficiency and Dysfunction. Immunity 45, 374–388. 10.1016/j.immuni.2016.07.009. 27496732 PMC5207350

[64] Mackensen A, Haanen JBAG, Koenecke C, Alsdorf W, Wagner-Drouet E, Heudobler D, Borchmann P, Bokemeyer C, Klobuch S, Smit E, (2022). LBA38 BNT211–01: A phase I trial to evaluate safety and efficacy of CLDN6 CAR T cells and CLDN6-encoding mRNA vaccine-mediated in vivo expansion in patients with CLDN6-positive advanced solid tumours. Ann Oncol 33, S1404–S1405. 10.1016/j.annonc.2022.08.035.

[65] Haanen J, Mackensen A, Koenecke C, Alsdorf W, Desuki A, Wagner-Drouet E, Heudobler D, Borchmann P, Wiegert E, Schulz C, (2021). LBA1 BNT211: A phase I/II trial to evaluate safety and efficacy of CLDN6 CAR-T cells and CARVac-mediated in vivo expansion in patients with CLDN6+ advanced solid tumors. Ann Oncol 32, S1392. 10.1016/j.annonc.2021.10.216.

[66] Snook AE (2020). Companion vaccines for CAR T-cell therapy: applying basic immunology to enhance therapeutic efficacy. Future Med Chem 12, 1359–1362. 10.4155/fmc-2020-0081. 32597219

[67] Kang BH, Momin N, Moynihan KD, Silva M, Li Y, Irvine DJ, and Wittrup KD (2021). Immunotherapy-induced antibodies to endogenous retroviral envelope glycoprotein confer tumor protection in mice. Plos One 16, e0248903. 10.1371/journal.pone.0248903. 33857179 PMC8049297

[68] Grace BE, Backlund CM, Morgan DM, Kang BH, Singh NK, Huisman BD, Rappazzo CG, Moynihan KD, Maiorino L, Dobson CS, (2022). Identification of Highly Cross-Reactive Mimotopes for a Public T Cell Response in Murine Melanoma. Front Immunol 13, 886683. 10.3389/fimmu.2022.886683. 35812387 PMC9260506

[69] Schindelin J, Arganda-Carreras I, Frise E, Kaynig V, Longair M, Pietzsch T, Preibisch S, Rueden C, Saalfeld S, Schmid B, (2012). Fiji: an open-source platform for biological-image analysis. Nat Methods 9, 676–682. 10.1038/nmeth.2019. 22743772 PMC3855844

[70] Moynihan KD, Opel CF, Szeto GL, Tzeng A, Zhu EF, Engreitz JM, Williams RT, Rakhra K, Zhang MH, Rothschilds AM, (2016). Eradication of large established tumors in mice by combination immunotherapy that engages innate and adaptive immune responses. Nature Medicine 22, 1402–1410. 10.1038/nm.4200. PMC5209798 27775706

[71] Clipstone NA, and Crabtree GR (1992). Identification of calcineurin as a key signalling enzyme in T-lymphocyte activation. Nature 357, 695–697. 10.1038/357695a0. 1377362

[72] Liu H, Moynihan KD, Zheng Y, Szeto GL, Li AV, Huang B, Egeren DSV, Park C, and Irvine DJ (2014). Structure-based programming of lymph-node targeting in molecular vaccines. Nature 507, 519–522. 10.1038/nature12978. 24531764 PMC4069155

[73] Patro R, Duggal G, Love MI, Irizarry RA, and Kingsford C (2017). Salmon provides fast and bias-aware quantification of transcript expression. Nat Methods 14, 417–419. 10.1038/nmeth.4197. 28263959 PMC5600148

[74] Soneson C, Love MI, and Robinson MD (2016). Differential analyses for RNA-seq: transcript-level estimates improve gene-level inferences. F1000research 4, 1521. 10.12688/f1000research.7563.2. PMC4712774 26925227

[75] Love MI, Huber W, and Anders S (2014). Moderated estimation of fold change and dispersion for RNA-seq data with DESeq2. Genome Biol 15, 550. 10.1186/s13059-014-0550-8. 25516281 PMC4302049

[76] Anders S, and Huber W (2010). Differential expression analysis for sequence count data. Genome Biol 11, R106–R106. 10.1186/gb-2010-11-10-r106. 20979621 PMC3218662

[77] Zhu A, Ibrahim JG, and Love MI (2019). Heavy-tailed prior distributions for sequence count data: removing the noise and preserving large differences. Bioinformatics 35, 2084–2092. 10.1093/bioinformatics/bty895. 30395178 PMC6581436

[78] Mootha VK, Lindgren CM, Eriksson K-F, Subramanian A, Sihag S, Lehar J, Puigserver P, Carlsson E, Ridderstråle M, Laurila E, (2003). PGC-1α-responsive genes involved in oxidative phosphorylation are coordinately downregulated in human diabetes. Nat Genet 34, 267–273. 10.1038/ng1180. 12808457

[79] Subramanian A, Tamayo P, Mootha VK, Mukherjee S, Ebert BL, Gillette MA, Paulovich A, Pomeroy SL, Golub TR, Lander ES, (2005). Gene set enrichment analysis: A knowledge-based approach for interpreting genome-wide expression profiles. Proc National Acad Sci 102, 15545–15550. 10.1073/pnas.0506580102. PMC1239896 16199517

[80] Hughes TK, Wadsworth MH, Gierahn TM, Do T, Weiss D, Andrade PR, Ma F, Silva B.J. de A., Shao S, Tsoi LC, (2020). Second-Strand Synthesis-Based Massively Parallel scRNA-Seq Reveals Cellular States and Molecular Features of Human Inflammatory Skin Pathologies. Immunity 53, 878–894.e7. 10.1016/j.immuni.2020.09.015. 33053333 PMC7562821

[81] Stoeckius M, Zheng S, Houck-Loomis B, Hao S, Yeung BZ, Mauck WM, Smibert P, and Satija R (2018). Cell Hashing with barcoded antibodies enables multiplexing and doublet detection for single cell genomics. Genome Biol 19, 224. 10.1186/s13059-018-1603-1. 30567574 PMC6300015

[82] Macosko EZ, Basu A, Satija R, Nemesh J, Shekhar K, Goldman M, Tirosh I, Bialas AR, Kamitaki N, Martersteck EM, (2015). Highly Parallel Genome-wide Expression Profiling of Individual Cells Using Nanoliter Droplets. Cell 161, 1202–1214. 10.1016/j.cell.2015.05.002. 26000488 PMC4481139

[83] Ma L, Shan Y, Bai R, Xue L, Eide CA, Ou J, Zhu LJ, Hutchinson L, Cerny J, Khoury HJ, (2014). A therapeutically targetable mechanism of BCR-ABL-independent imatinib resistance in chronic myeloid leukemia. Science translational medicine 6, 252ra121–252ra121. 10.1126/scitranslmed.3009073. PMC4162097 25186176

[84] Tu AA, Gierahn TM, Monian B, Morgan DM, Mehta NK, Ruiter B, Shreffler WG, Shalek AK, and Love JC (2019). TCR sequencing paired with massively parallel 3′ RNA-seq reveals clonotypic T cell signatures. Nat Immunol 20, 1692–1699. 10.1038/s41590-019-0544-5. 31745340 PMC7528220

[85] Gupta NT, Heiden JAV, Uduman M, Gadala-Maria D, Yaari G, and Kleinstein SH (2015). Change-O: a toolkit for analyzing large-scale B cell immunoglobulin repertoire sequencing data. Bioinformatics 31, 3356–3358. 10.1093/bioinformatics/btv359. 26069265 PMC4793929

[86] Heiden JAV, Yaari G, Uduman M, Stern JNH, O’Connor KC, Hafler DA, Vigneault F, and Kleinstein SH (2014). pRESTO: a toolkit for processing high-throughput sequencing raw reads of lymphocyte receptor repertoires. Bioinformatics 30, 1930–1932. 10.1093/bioinformatics/btu138. 24618469 PMC4071206

[87] Smith T, Heger A, and Sudbery I (2017). UMI-tools: modeling sequencing errors in Unique Molecular Identifiers to improve quantification accuracy. Genome Res 27, 491–499. 10.1101/gr.209601.116. 28100584 PMC5340976

[88] Charan J, and Kantharia ND (2013). How to calculate sample size in animal studies? J Pharmacol Pharmacother 4, 303–306. 10.4103/0976-500x.119726. 24250214 PMC3826013

[89] Davila ML, Kloss CC, Gunset G, and Sadelain M (2013). CD19 CAR-Targeted T Cells Induce Long-Term Remission and B Cell Aplasia in an Immunocompetent Mouse Model of B Cell Acute Lymphoblastic Leukemia. PLOS ONE 8, e61338–14. 10.1371/journal.pone.0061338. 23585892 PMC3621858

