# Supplementary figures and images for: Vaccine-boosted CAR T crosstalk with host immunity to reject tumors with antigen heterogeneity
Source: Cell. Author manuscript; Available in PMC 2023 Jul 27. (PMC10372881; doi:10.1016/j.cell.2023.06.002)

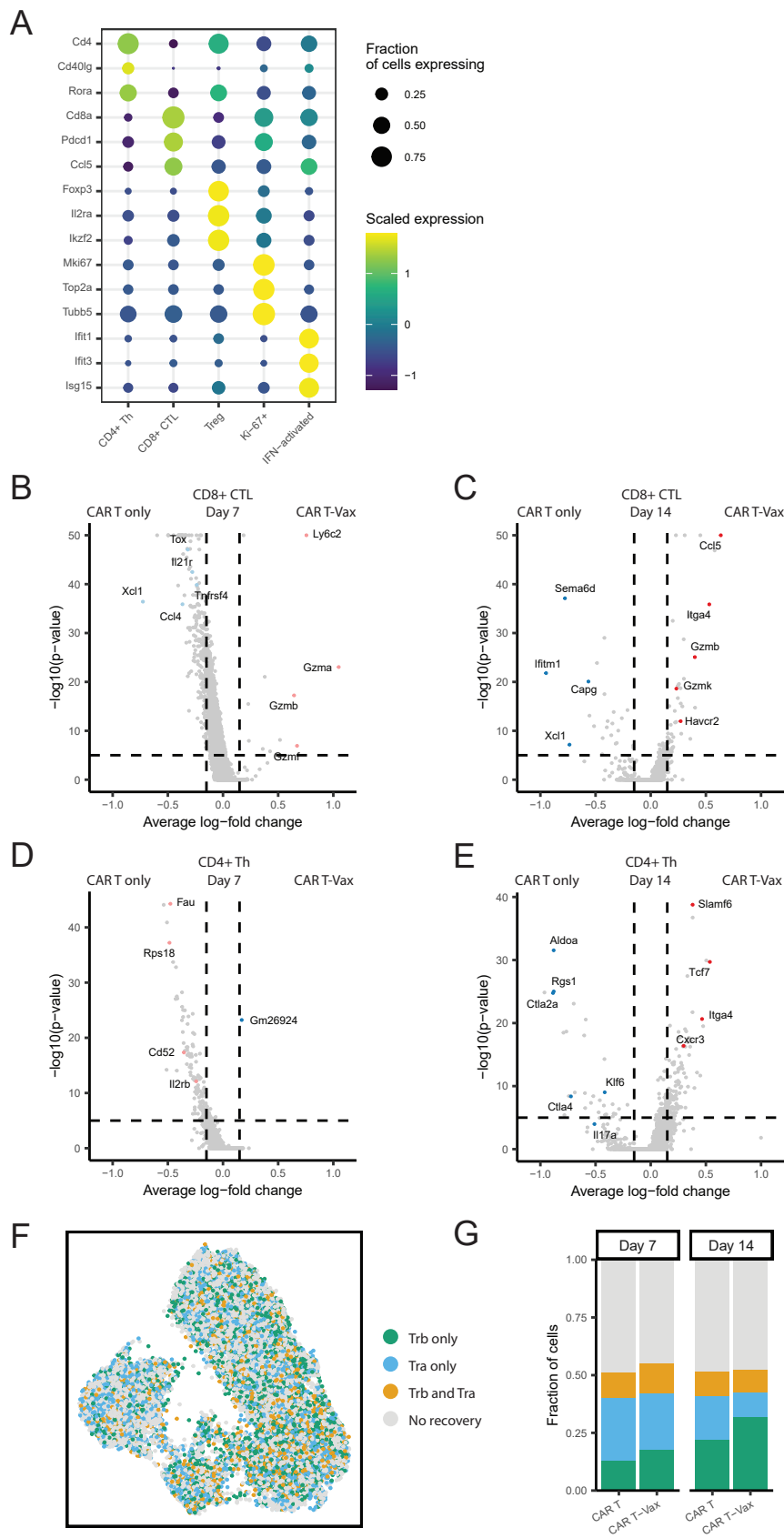

Supplement: 1 — (A) Schematic of the development of anti-EGFRvIII CAR and amph-pepvIII vaccine. (B) Kinetics of the recovery of T cell and DC populations post sublethal irradiation (500 cGy) in C57BL/6 mice. Day 0 denotes the baseline level prior to irradiation. Arrow indicates day of irradiation. Shown is one representative of at least three independent experiments. (C) Impact of tumor location and site of vaccination on the magnitude of CAR T-vax induced antigen spreading. C57BL/6 mice bearing EGFRvIII+CT-2A tumors received lymphodepletion (LD) followed by treatment with control (untransduced) T-cells or CAR T-vax following the same timeline as in Fig 1C. IFN-γ ELISPOT was assayed for splenic T cells isolated on day 21 and stimulated with irradiated EGFRvIII-negative CT-2A cells. Shown are representative ELISPOT well images and quantitative ELISPOT data from one representative of two independent experiments. (D) Impact of adjuvants on eliciting CAR T-vax induced antigen spreading. EGFRvIII+CT-2A tumor-bearing C57BL/6 mice received lymphodepletion (LD) and subsequent treatment with CAR-T in the absence or presence of amph-pepvIII vaccine formulated with different adjuvants administered following the same timeline as in Fig. 1C. Shown is IFN-γ ELISPOT monitoring endogenous T-cell priming across various conditions at day 21 as in (C). (E) Schematic of CAR T-vax therapy using a combination of amph-FITC vaccine and FITC/TA99 bispecific CAR T-cells. (F) Tumor antigen specificity of endogenous TILs in mice receiving CAR T vs CAR T-vax therapy. C57BL/6 mice bearing OVA-expressing EGFRvIII+CT-2A tumors received lymphodepletion and CAR T transfer followed by two weekly vaccinations. Upper panel, experimental timeline. Lower panel, representative flow cytometry plots showing SIINFEKL-tetramer staining of endogenous CD8+ T cells within tumors isolated from mice treated with CAR T or CAR T-vax therapy on day 21. Throughout, n=5 animals/group. In Panel C-D, H, data shown are mean ± 95% CI. **, [file NIHMS1908849-supplement-1.pdf]

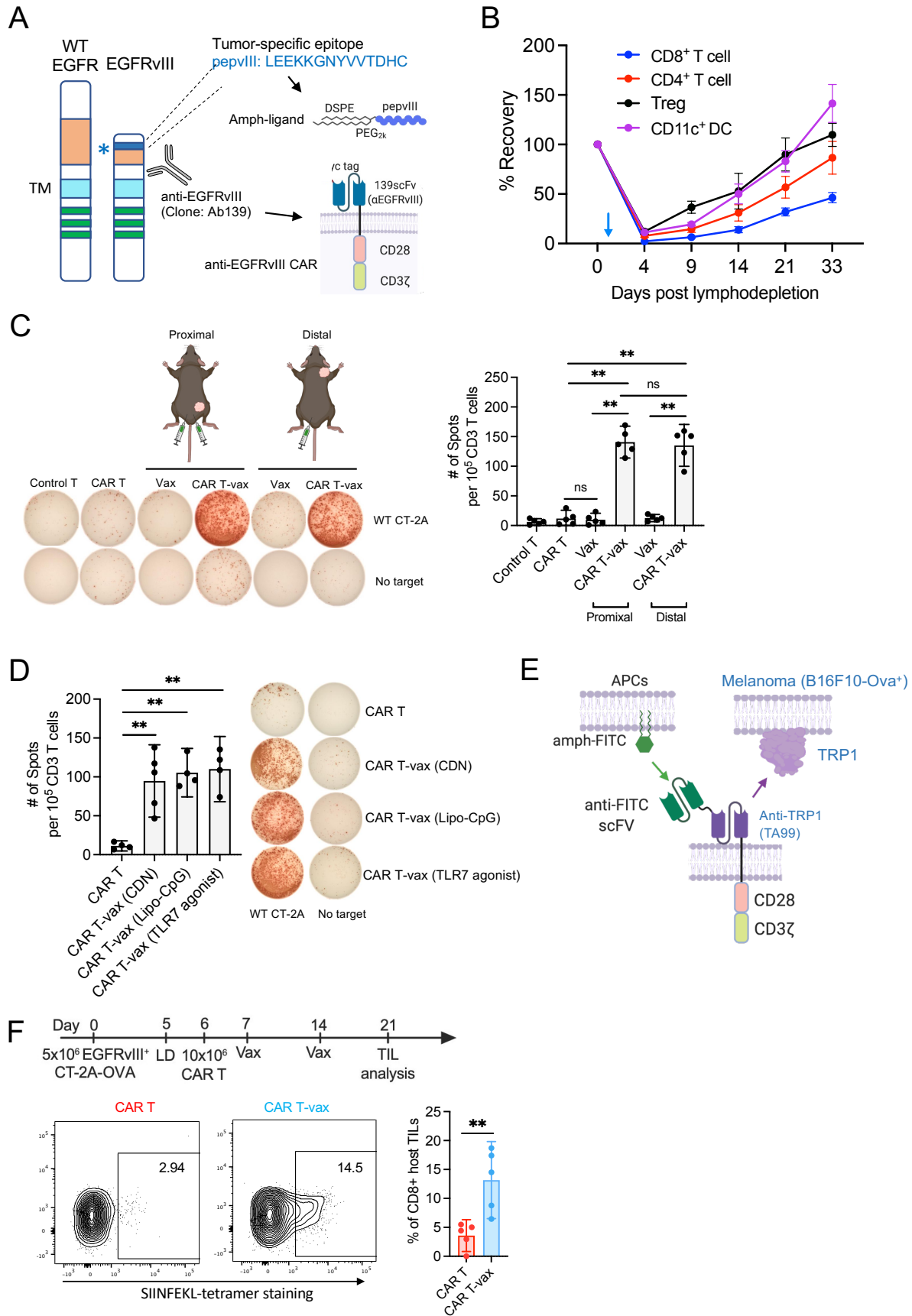

Supplement: 2 — (A) Dot plot showing differential expression of selected genes in different T cell subtypes as a result of vaccine boosting of CAR T-cells in Fig 2. (B) Volcano plot showing differential gene expression in intratumoral CD8+ CTLs between CAR T-vax vs. CAR-T alone group on day 7 in Fig 2. (C) Volcano plot showing differential gene expression in intratumoral CD8+ CTLs between CAR T-vax and CAR-T alone group on day 14 in Fig 2. (D) Volcano plot showing differential gene expression in intratumoral CD4+ Th cells between CAR T-vax and CAR-T alone group on day 7 in Fig 2. (E) Volcano plot showing differential gene expression in intratumoral CD4+ Th cells between CAR T-vax and CAR-T alone group on day 14 in Fig 2. (F) UMAP of T cells with detectable TCR alpha, beta or both chains. TCR data was extracted from the scRNA-seq data of Fig 2. (G) Stacked charts showing proportions of T cells with detectable TCR alpha, beta or both chains on day 7 and day 14, respectively. [file NIHMS1908849-supplement-2.pdf]

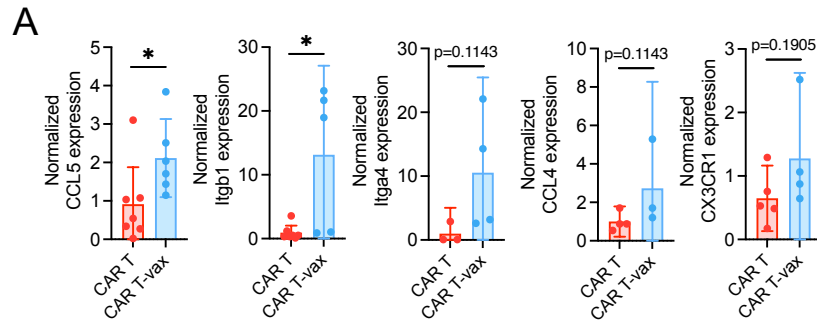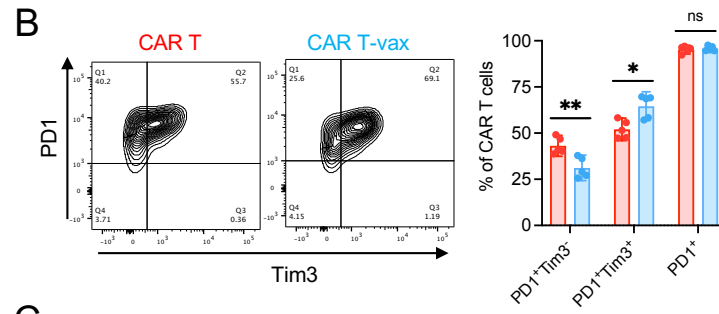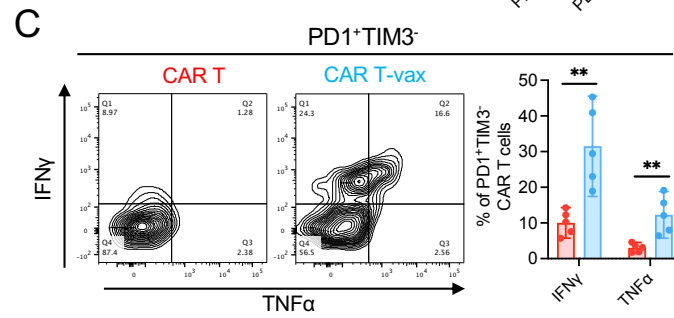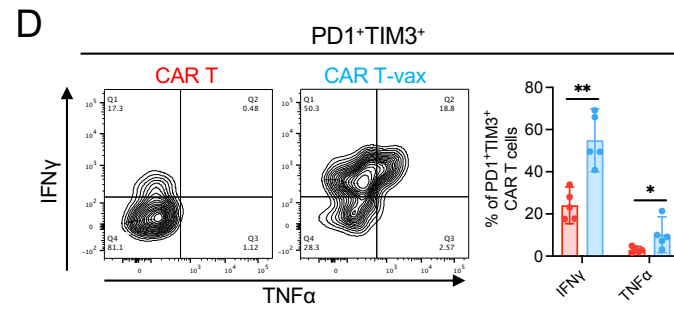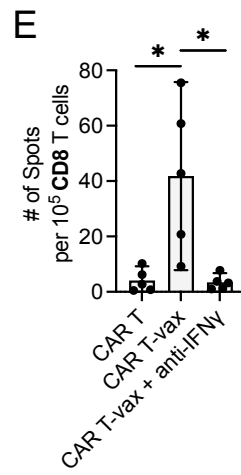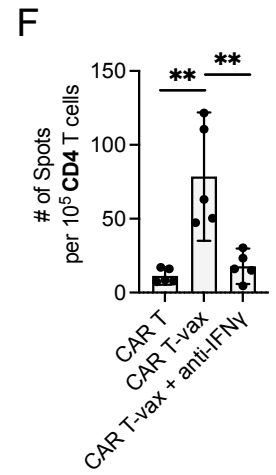

Supplement: 3 — (A) CD45.2+ C57BL/6 mice bearing EGFRvIII+CT-2A tumors were treated by CD45.1+ EGFRvIII-CAR T ± vax as in Fig 2B. On day 7 post therapy, tumor-infiltrating endogenous CD8+ T cells were analyzed for checkpoint marker (PD1, Tim3) expression by flow cytometry. Shown is one representative of two independent experiments. (B) Cytokine polyfunctionality in PD1+TIM3− and PD1+TIM3+ tumor-infiltrating endogenous CD45.2+ CD8+ T cells from mice in Fig. S3A. Tumors were dissociated into single cell suspensions on day 7 post therapy and cultured in the presence of 1x cell stimulation cocktail and Golgi plug for 6 hours followed by intracellular cytokine staining and flow cytometry analysis. (C) Granzyme B expression in PD1+TIM3− and PD1+TIM3+ tumor-infiltrating endogenous CD45.2+ CD8+ T cells from mice in Fig. S3A and analyzed via intracellular staining as in Fig. S3B. (D) Histogram showing retroviral Env expression on CT-2A cells, MC38 cells and TC-1 cells. Cells were stained with anti-Env primary antibody followed by anti-IgG isotype secondary antibody. MC38 and TC-1 cells were included as positive and negative controls67, respectively. (E) mouse IL-2 ELISA showing p15E antigen expression in CT-2A cells as detected by T cells. CT-2A cells, MC38 cells and TC-1 cells were pre-treated with IFN-γ and co-cultured with 58 T-cell hybridoma expressing an irrelevant 2C TCR or a p15E-reactive 7PPG2 TCR at 1:1 E:T ratio for 24hr. The supernatant was collected for ELISA. (F) CDR3 sequences and gene usage for top-ranked p15E-specific T cell clones based on the CDR3 sequences and consensus motifs reported in Grace et al 68. The consensus motifs were underlined in CDR3α or CDR3β sequences. (G) Long-term growth of tumors in WT (n=10 animals/group) or Rag1−/− mice (n=5 animals/group) in Fig 3H. Only the EGFRvIII+ CT-2A and EGFRvIII− CT-2A 100:0 ratio group is shown here. (H-I) C57BL/6 mice bearing mixed (80% WT + 20% TRP1−/−) B16F10 tumors received lymphodepletion (LD) and were left untreated o [file NIHMS1908849-supplement-3.pdf]

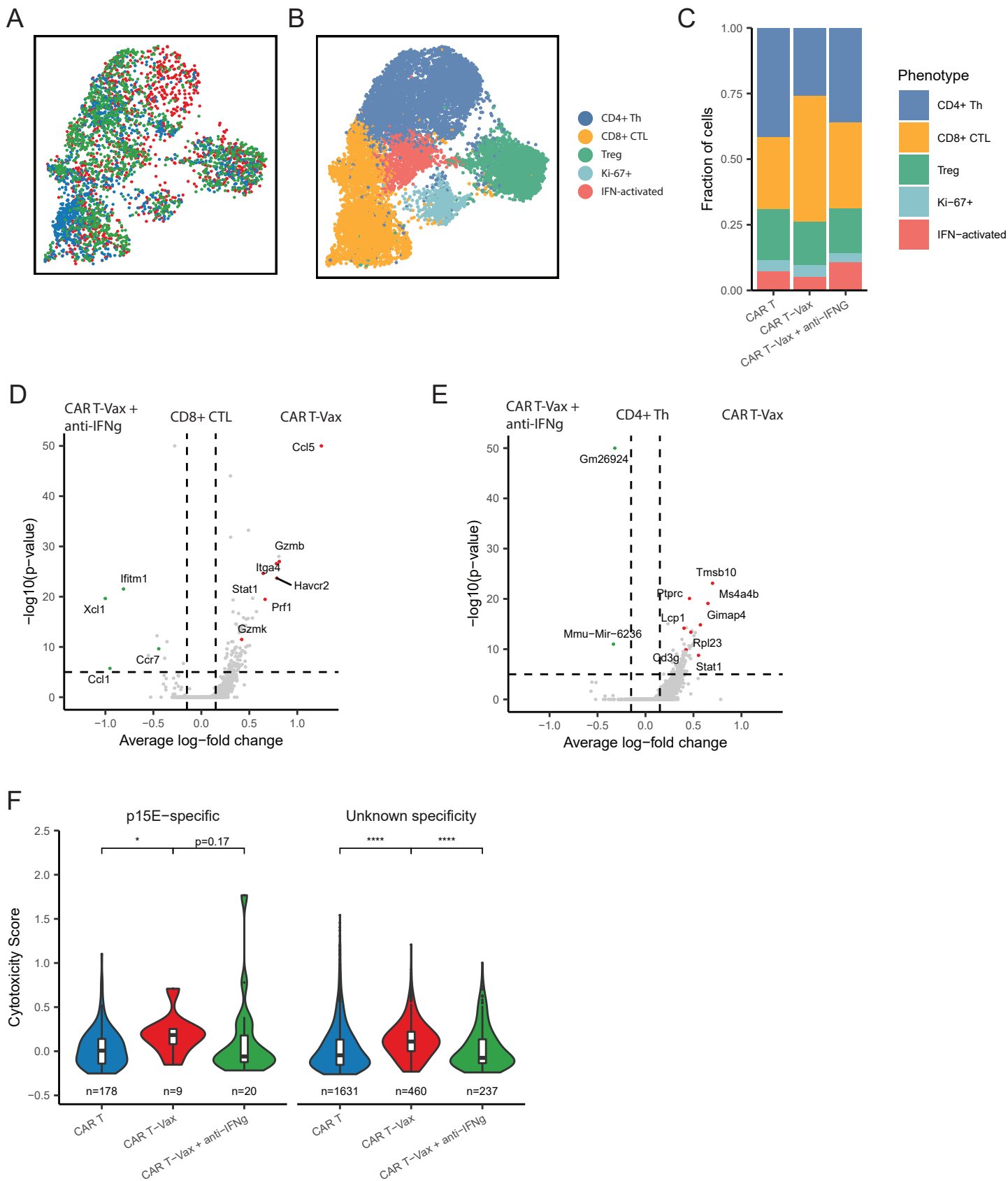

Supplement: 4 — (A) CD45.2 EGFRvIII+CT-2A tumor-bearing mice (n=3–7 animals/group) were treated with CD45.1 EGFRvIII CAR T-cells ± vaccine boosting. Seven days later, splenic CAR T-cells were purified by FACS and processed for quantitative real-time PCR. Gene expression in individual samples from both groups were normalized to the average gene expression in T cells from the CAR T-treated group. Shown are the fold change in the expression of representative genes. (B) CD45.2+ C57BL/6 mice bearing EGFRvIII+CT-2A tumors were treated by CD45.1+ EGFRvIII-CAR T ± vax as in Fig. 5A. On day 7 post therapy, tumor-infiltrating CAR T-cells were analyzed for checkpoint marker (PD1, Tim3) expression by flow cytometry. Shown is one representative of two independent experiments. (C-D) Cytokine polyfunctionality in PD1+TIM3− (C) and PD1+TIM3+ (D) tumor-infiltrating CD45.1+ CAR T-cells from mice in Fig. S4B. Tumors were dissociated into single cell suspensions on day 7 post therapy and cultured in the presence of 1x cell stimulation cocktail and Golgi plug for 6 hours followed by intracellular cytokine staining and flow cytometry analysis. (E-F) C57BL/6 mice bearing EGFRvIII+CT-2A tumors received lymphodepletion (LD) and were treated with CAR T, or CAR T-vax ± anti-IFNγ. Priming of endogenous CD8 (E) or CD4 (F) T cells in each condition was assessed by IFN-γ ELISPOT as in Fig. 1C. Throughout, n=5 animals/group, error bars are mean ± 95% CI, **p<0.01, *p<0.05, n.s not significant by Student’s t-test for A-D, or by one-way ANOVA with Tukey’s post-test for E-F. [file NIHMS1908849-supplement-4.pdf]

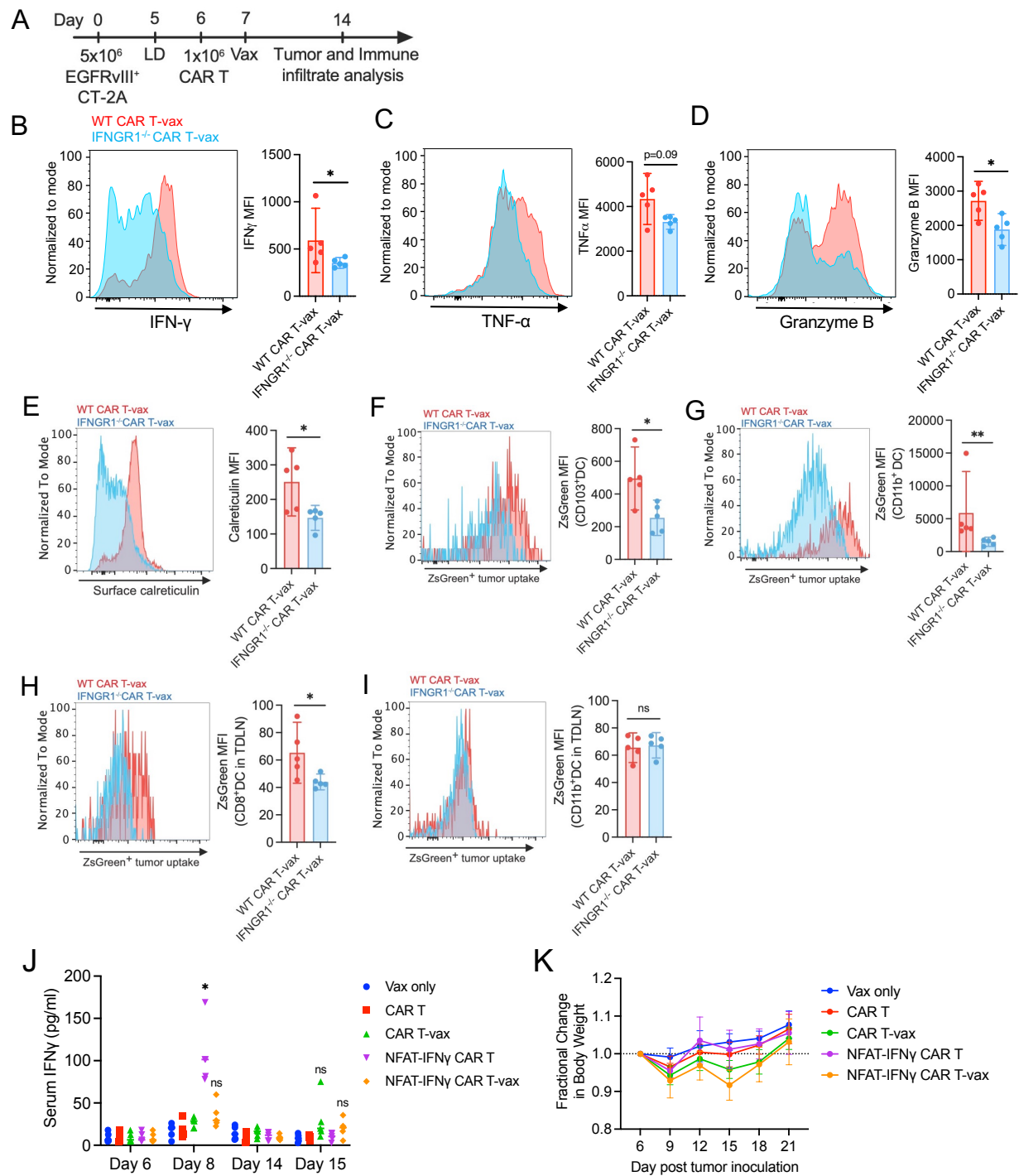

Supplement: 5 — (A) UMAP of endogenous T cells obtained from the day 14 tumors in CAR-T- and CAR T-vax-treated mice in Fig. 2 as well as a group of mice receiving CAR T-vax + anti-IFNγ treatment. T cells were randomly down-sampled to show an even number of points from each treatment condition. T cells are colored by the treatment group. (B) Curated clusters based on signature gene expression of day 14 T cells in Fig. S5A. Th, T helper cells. Treg, regulatory T cell. CTL, cytotoxic lymphocyte. (C) Stacked charts showing proportions of different clusters within day 14 T cells under each treatment condition in Fig. S5A. (D) Volcano plot showing differential gene expression in day 14 CD8+ CTLs between CAR T-vax and CAR T-vax + anti IFNγ group in Fig. S5A. (E) Volcano plot showing differential gene expression in day 14 CD4+ Th cells between CAR T-vax and CAR T-vax + anti IFNγ group in Fig.S5A. (F) Cytotoxicity score of endogenous retroviral antigen p15E-specific TILs and TILs of unknown specificity on day 14 from CAR T, CAR T-vax and CAR T-vax + IFNγ groups. Data shown are mean ± 95% CI. ****, p<0.0001; *, p<0.05 by two-sided Wilcoxon rank-sum test. See methods for the definition and calculation of the cytotoxicity score. [file NIHMS1908849-supplement-5.pdf]

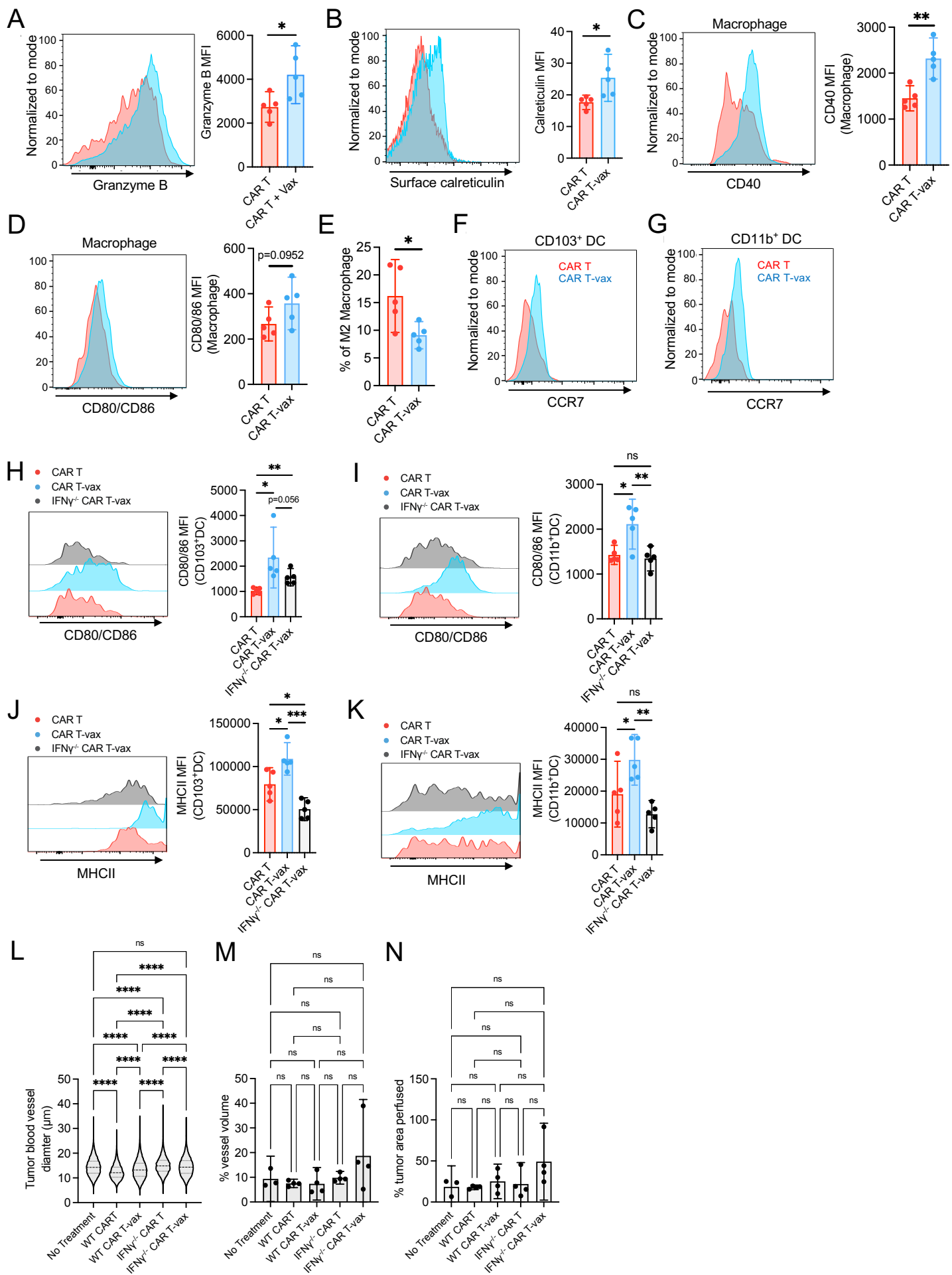

Supplement: 7 — (A-H) CD45.1+ C57BL/6 mice bearing EGFRvIII+CT-2A tumors received lymphodepletion (LD) and were treated with CD45.2+ WT or IFNGR1−/−CAR T-vax as shown in the timeline (A). Tumors isolated from each group at day 7 post CAR T-vax therapy were dissociated into single cell suspensions for flow cytometry analysis. (B) IFN-γ expression in CD45.2+ CAR T-cells from WT or IFNGR1−/− CAR T-vax treated group. (C)TNF-α expression in CD45.2+ CAR T-cells from WT or IFNGR1−/− CAR T-vax treated group. (D) Granzyme B expression in CD45.2+ CAR T-cells from WT or IFNGR1−/− CAR T-vax treated group. (D) Flow cytometry analysis showing surface expression of calreticulin on tumor cells. (E) Flow cytometry analysis showing tumor antigen uptake by intratumoral CD45.2+ CD103+DCs. ZsGreen was used as a surrogate antigen in this experiment. (F) Flow cytometry analysis showing tumor antigen uptake by intratumoral CD45.2+ CD11b+ DCs. ZsGreen was used as a surrogate antigen in this experiment. (G) Flow cytometry analysis showing tumor antigen uptake by LN-resident CD45.2+ CD8+ DCs. ZsGreen was used as a surrogate antigen in this experiment. (H) Flow cytometry analysis showing tumor antigen uptake by LN-resident CD45.2+ CD11b+ DCs. ZsGreen was used as a surrogate antigen in this experiment. (J-K) C57BL/6 mice bearing mixed CT-2A tumor (80% EGFRvIII+ CT-2A + 20% WT CT2A cells) were treated as in Fig. 1C with vaccine only, CAR T or NFAT-IFNγ CAR T in the presence or absence of vaccination. Shown are IFN-γ levels in serum (J) from day 6 (before 1st vax), day 8 (24hr post 1st vax) and day 14 (day 6 post 1st vax) and day 15 (24hr post 2nd vax) and animal body weight change (K). Throughout, a representative histogram from each treatment group and the summary data are shown. n=5 animals/group, data shown are mean ± 95% CI. **, p<0.01; *, p<0.05; ns, not significant by Student’s t-test for B-I, and by two-way ANOVA with Turkey’s multiple comparisons test for J. [file NIHMS1908849-supplement-7.pdf]
