# Supplementary material for: Vaccine-boosted CAR T crosstalk with host immunity to reject tumors with antigen heterogeneity
Source: Cell. Author manuscript; Available in PMC 2023 Jul 27. (PMC10372881; doi:10.1016/j.cell.2023.06.002)

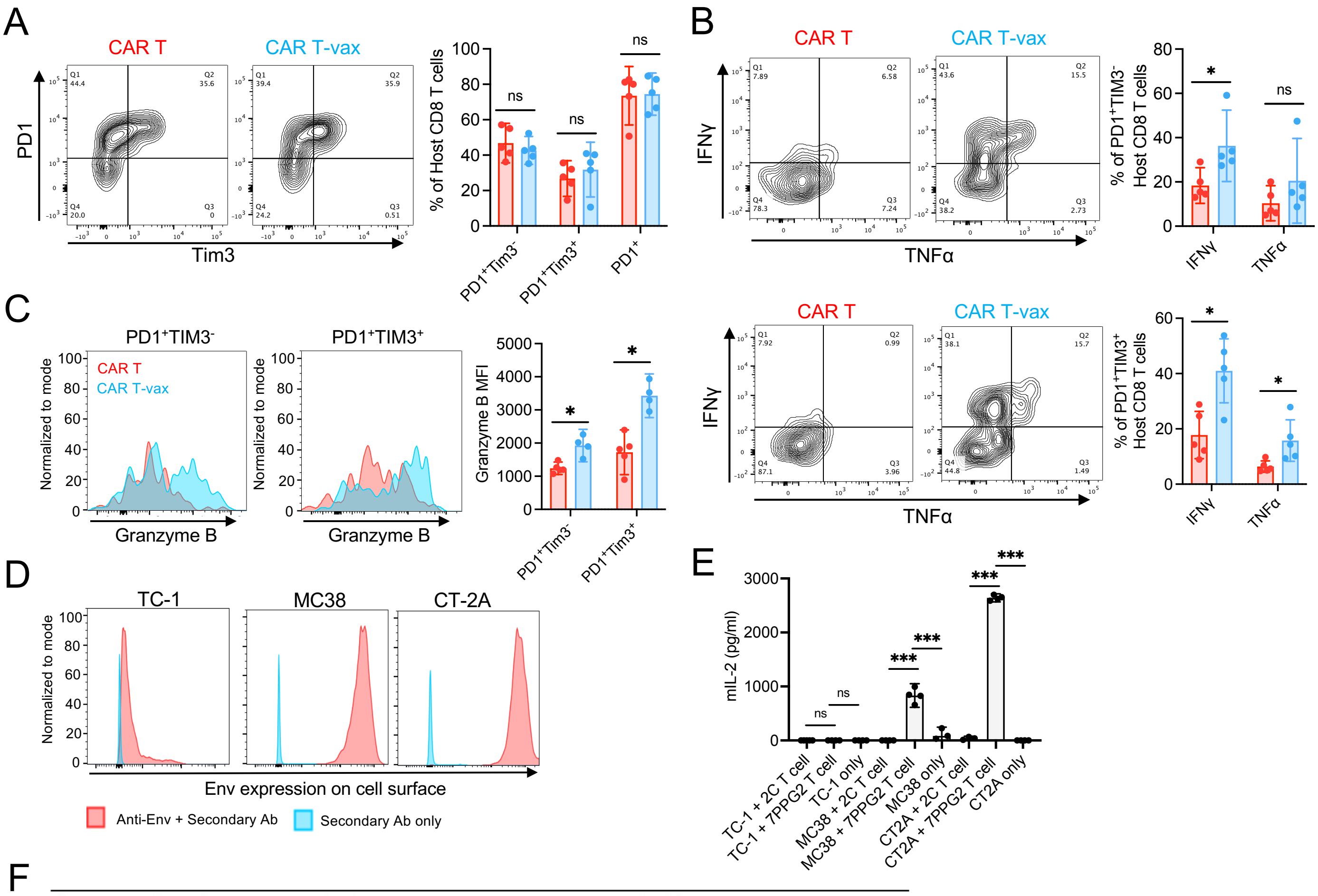

F

Clone Name	Clone size	TRAV	CDR3α	TRAJ	TRBV	CDR3β	TRBJ
7PEQ27	66	TRAV7-2	CAASDYSNNRLT	TRAJ7	TRBV16	CASSLELGGPEQYF	TRBJ2-7
7LEQ27	52	TRAV3D-3	CAVTPDYSNNRLT	TRAJ7	TRBV16	CASSLELGGLEQYF	TRBJ2-7
7QTG27	42	TRAV8D-2	CATDYSNNRLT	TRAJ7	TRBV12-1	CASSQTGGPSYEQYF	TRBJ2-7
7LEV24	41	TRAV6-6	CALLPADYSNNRLT	TRAJ7	TRBV16	CASSLEVGQGYSQNTLYF	TRBJ2-4
7REQ27	31	TRAV7-2	CAGSDYSNNRLT	TRAJ7	TRBV16	CASSLELGGREQYF	TRBJ2-7
27LEG27	27	TRAV6-4	CALANTNTGKLTF	TRAJ27	TRBV16	CASSLELGGLEQYF	TRBJ2-7
7LRG15	15	TRAV13D-1	CALGDYSNNRLT	TRAJ7	TRBV3	CASSLRGGDQAPLF	TRBJ1-5
7QEG11	14	TRAV5D-4	CAAKDYSNNRLT	TRAJ7	TRBV2	CASSQEGANTEVFF	TRBJ1-1
7QTG25	13	TRAV9N-3	CAVFPDYSNNRLT	TRAJ7	TRBV2	CASSQTGGGDTQYI	TRBJ2-5
N7LEQ27	11	TRAV7-2	CAANDYSNNRLT	TRAJ7	TRBV16	CASSLELGGLEQYF	TRBJ2-7

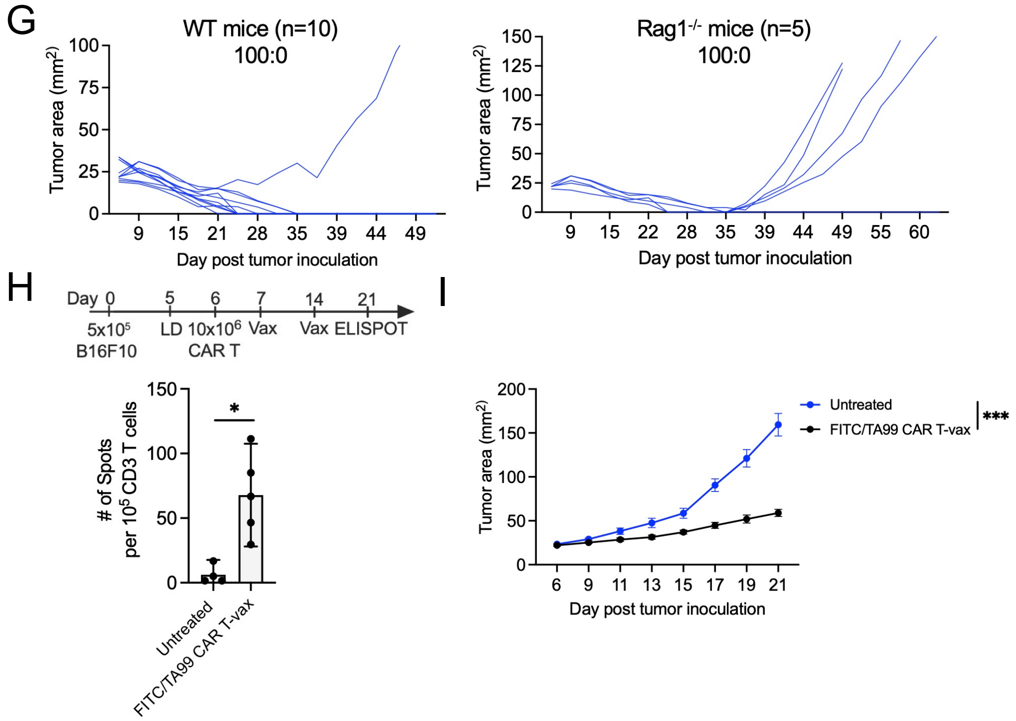

Supplement: 6 — (A) Flow cytometry analysis showing intracellular granzyme B expression in CAR T-cells from mice in Fig. 6B. (B) Tumors isolated from each group in Fig. 6B were dissociated into single cell suspensions for flow cytometry analysis of surface calreticulin expression on tumor cells in response to CAR T vs. CAR T-vax therapy. (C-E) Intratumoral macrophage phenotypes are shifted by CAR T-vax therapy. C57BL/6 mice bearing EGFRvIII+CT-2A tumors (n = 5 animals/group) were treated by WT CAR T ± vax as in Fig 6B, and intratumoral macrophages were analyzed for CD40 (C), CD80/86 (D) levels as well as the percentage of suppressive M2 (CD11b+F4/80+CD206+) macrophages (E) by flow cytometry on day 14 as shown in the timeline. See methods for surface markers used to define M2 macrophages. (F) Representative histogram showing surface CCR7 expression on intratumoral CD103+ DCs from mice in Fig. 6F. (G) Representative histogram showing surface CCR7 expression on intratumoral CD11b+ DCs from mice in Fig. 6F. (H-I) Flow cytometry analysis showing CD80/CD86 expression in intratumoral CD103+ DCs (H) or CD11b+ DCs (I) from mice in Fig. 6G–H. (J-K) Flow cytometry analysis showing MHCII expression in intratumoral CD103+ DCs (J) or CD11b+ DCs (K) from mice in Fig. 6G–H. (L-N) CAR T-vax therapy using WT or IFN-γ KO CAR T cells does not lead to substantial changes in the tumor vasculature. Total vessel diameter (L) and vessel volume (M) was calculated using the Filament tracing algorithm on the masked anti-CD31 channel. Hoechst area were calculated using the Analyze particles function in Fiji69 as % of tumor area perfused (N). Throughout, n=5 animals/group. Shown are representative flow histograms and geom. mean fluorescence intensities. Data shown are mean ± 95% CI. ****, p<0.0001; ***, p<0.001, **, p<0.01, *, p<0.05 by Student’s t-test for A-E, and by one-way ANOVA with Tukey’s post-test for H-N. [file NIHMS1908849-supplement-6.pdf]
